# Supplementary material for: Diagnostic routes and time intervals for patients with colorectal cancer in 10 international jurisdictions; findings from a cross-sectional study from the International Cancer Benchmarking Partnership (ICBP)
Source: BMJ Open. 2018 Nov 27;8(11):e023870. doi: 10.1136/bmjopen-2018-023870 (PMC6278806; doi:10.1136/bmjopen-2018-023870)
Supplement: Supplementary file 1 [file bmjopen-2018-023870supp001.pdf]

Supplementary file 1: ICBPM4 Rules for missing, incomplete, multiple response and out of range data

1. <u>Oversampling/Participation in local screening trials</u>
   a) To handle oversampling in Ontario, include only the first 360 consecutive CRC patients;
   b) In jurisdictions with no national screen program: exclude patients participated in local screen trials.

2. <u>Language/Participation in study/Presence of cancer</u>
   Exclude patients who checked "No, I don't understand the language" or "I don't want to participate in this study" or "I don't have cancer".

3. <u>Survey responders</u>
   a) Exclude Patient/PCP/Specialist survey from the analysis, if it was not written by Patient/PCP/Specialist (example: a medical oncologist completed a PCP survey);
   b) In the case of duplicates, include only the first survey (example: 2 specialists completed surveys for the same patient).

4. <u>Gender</u>
   Exclude patients with unknown Gender.

5. <u>Age</u>
   a) Exclude patients with unknown age;
   b) Exclude patients younger 40 years;
   c) Use registry data, if Age is reported by both patient and registry.

6. <u>No cancer or Previous cancer in the same organ</u>
   a) Exclude patients with no cancer based on registry data;
   b) Exclude patients with previous cancer in the same organ based on data from registry or free-text for Presentation in the patient survey.

7. <u>Date of consent</u>
   Exclude patients with date of consent which is unknown, before 01.01.2013 or in the future.

8. <u>Multiple responses to Dates</u>
   If multiple responses were given to the dates (of first symptom; screening; first presentation to primary care; referral; diagnosis; treatment start), then use the earliest date.

9. <u>Order of Dates</u>
   The dates must be in the following order –
   a) First symptom; first presentation to Primary Care; referral; diagnosis; treatment start.
   b) Screening; diagnosis; treatment start.

   If not, check for mistakes.

10. <u>Date of first symptom</u>
    Date of first symptom is defined as date of first symptom from patient data.

11. <u>Date of first presentation</u>
    Date of first presentation to Primary Care is defined as (in the order of declining priority):
    a) date of first presentation to Primary Care from PCP data;
    b) date of first presentation to Primary Care and A&E from PCP data;
    c) date of first presentation to Primary Care from patient data.

12. <u>Date of referral</u><br>Date of referral is defined as date of referral from PCP data.
13. <u>Date of screening</u><br>Date of screening is defined as (in the order of declining priority):<br>a) date of screening from registry;<br>b) date of screening from patient data.
14. <u>Date of diagnosis</u><br>*Definition*<br>a) If Registry reports both date of histological confirmation and date of confirming investigation, then use date of histological confirmation.<br>b) Date of diagnosis (based on patient data, PCP data, specialist data, registry data) is defined as (in the order of declining priority):<br>- date of diagnosis from registry;<br>- date of histological confirmation (from specialist data, PCP data);<br>- date of biopsy (from specialist data, PCP data);<br>- date of confirming investigation (from specialist data, PCP data);<br>- date of first hospital admission (from specialist data, PCP data);<br>- date of MDT confirmation (from specialist data, PCP data);<br>- date patient was told (from specialist data, PCP data);<br>- other date of diagnosis (from specialist data, PCP data, patient data);<br><br>Choose a Date from a lower level of hierarchy, if the Date from a higher level is after the Date of consent or more than 9 months (=271 days) before the Date of consent.<br><br>*Exclusion criteria*<br>a) Unknown date of diagnosis;<br>b) Date of diagnosis is after the date of consent;<br>c) Date of diagnosis is more than 9 months before the Date of consent.
15. <u>Date of treatment start</u><br>a) Date of treatment start from patient data is defined as the earliest of the treatment dates for Surgery, Chemo, Radio and Other;<br>b) Date of treatment start (based on registry data, specialist data, patient data) is defined as (in the order of declining priority):<br>- date of treatment start from registry data,<br>- date of treatment start from specialist data,<br>- date of treatment start from patient data,<br>- anticipated date of treatment from patient data.
16. <u>Imputation of missing day in the date</u><br>Imputation rules for missing day (given month and year are known):<br>a) Set missing day to ‘16’;<br>b) Consider adjacent dates in a backwards order (from “Treatment” to “First symptom”). For each pair of such adjacent dates: If dates are not in a logical order (e.g. “Treatment” is before “Diagnosis”), but month and year are the same in both dates, and the day was imputed to ‘16’ in one of the dates:<br>- Recode the day imputed earlier to ‘16’ to the day from the adjacent date.
17. <u>Considering time</u><br>If patient gave multiple answers to the “How long did you have symptoms before contacting a doctor?” question, then use the option with the shortest time interval.
18. <u>Delay arranging appointment</u><br>If patient gave multiple answers to the “How long did it take to get an appointment with PCP?” question, then

use the option with the shortest time interval.

19. Duration of symptoms

If PCP gave multiple answers to the "Duration of symptoms" question, then use the option with the shortest time interval.

20. Definition of Presentation

A. Define Presentation within a Data Source (Patient, PCP)

1. Review the free-text for Presentation (Patient, PCP) and re-code, if possible.
2. If PCP reports 'VisitPCP and AE' or 'VisitPCP' as Presentation and no symptoms, then check Patient's records. If Patient reports 'Screening' and no symptoms, then re-code Presentation for this case as 'Screening'.
3. If PCP reports 'Screening' as Presentation and at least one symptom (or "Duration of Symptoms"), then re-code Presentation to 'Other non-screen-detected'-option.
4. If PCP reports 'Other' as Presentation and at least one symptom (or "Duration of Symptoms"), then re-code Presentation to 'Other non-screen-detected'-option.
5. If Patient reports 'Screening' as Presentation and at least one symptom (or date of first symptom), then re-code Presentation to 'Other non-screen-detected'- option.
6. If Patient reports 'Other' as Presentation and at least one symptom (or date of first symptom or "Considering time" or "Delay arranging appointment", then re-code Presentation to 'Other non-screen-detected'-option.
7. In the case of multiple Presentation responses (Patient, PCP sources) - use a single option (in the order of declining priority):

a) 'VisitPCP and AE',
b) 'VisitPCP´, 'AE´ (if both 'VisitPCP´ and 'AE´ are given, then re-code as 'VisitPCP and AE'),
c) 'Other non-screen-detected',
d) 'Screening',
e) 'Investigation for another problem' ,
f) 'Other"

B. Define Presentation from Alternative Data

If Presentation hasn't been reported in either of data sources, then define it as (in the order of declining priority):

1. 'Other non-screen-detected ', if PCP reports at least one symptom (or "Duration of symptoms");
2. 'Other non-screen-detected ', if Patient reports at least one symptom (or date of first symptom);
3. 'Other non-screen-detected ', if Patient reports "Considering time" or "Delay arranging appointment" and no screening date;
4. 'Screening', if Patient reports screening date and no symptoms and no date of first symptom;
5. 'Other non-screen-detected ', if jurisdiction=England, Age <58 or >76 years.

C. Define Presentation from Data Source Hierarchy

1. In Wales, England, Scotland, N Ireland and Manitoba: if Registry reports 'Screening' – use Presentation data from Registry data.

2. In Wales, England, Scotland, N Ireland and Manitoba: if Registry reports 'No Screening' – use Presentation data from (in the order of declining priority):

   a) PCP data;
   b) Patient data;

   If PCP (or Patient, in the case of PCP data is not available) reports 'Screening', then code Presentation as 'Other non-screen-detected'. If information from PCP and Patient datasets is missing, then code Presentation as 'Other non-screen-detected'.

3. In Wales, England, Scotland, N Ireland and Manitoba: if screening status from Registry is missing – use Presentation data from (in the order of declining priority):

a) PCP data;
b) Patient data;

4. For Denmark, Norway, Ontario and Victoria – use Presentation data from (in the order of declining priority):
   a) PCP data;
   b) Patient data.

5. In Sweden – use Presentation data from Patient data.

21. Patient interval
The Patient interval for non-screen-detected patients is defined as (in the order of declining priority):
a) "Date of first presentation to Primary Care" minus "Date of first symptom";
b) If the interval in (a) is unknown or negative: Calculate the interval as the low boundary of "Considering time" plus the low boundary of "Delay arranging appointment";
c) If the interval in (a) is unknown or negative and the interval in (b) is unknown: Calculate the interval as the low boundary of "Duration of symptoms interval".

22. Primary Care interval
The Primary Care interval for non-screen-detected is defined as "Date of referral" minus "Date of first presentation to Primary Care".

23. Diagnostic interval
a) The Diagnostic interval for non-screen-detected is defined as "Date of diagnosis" minus "Date of first presentation to Primary Care";
b) The Diagnostic interval for screen-detected patients is defined as "Date of diagnosis" minus "Date of screening".

24. Treatment interval
The Treatment interval is defined as "Date of treatment start" minus "Date of diagnosis".

25. Total interval
a) The Total interval for non-screen-detected patients is defined as "Date of treatment start" minus "Date of first symptom";
b) The Total interval for screen-detected patients is defined as "Date of treatment start" minus "Date of screening".

26. Range of Time intervals
The time intervals (Patient, Primary Care, Diagnosis, Treatment, Total) must be in range 0-1 year.

If > 1 year: set the interval to 365 days
If negative: set the interval to 0.

For each jurisdiction calculate the number of imputations due to:
a) unknown day in a date (given known month and year);
b) very large(>1 year) interval;
c) negative interval.

27. Type of treatment
If patient ticked both "Yes" and "No" as answers to the "Type of treatment (Surgery, Chemotherapy, Radiotherapy)" questions, then choose "Yes" answer.

28. Health state
If patient gave multiple answers to the "Health state" question, then use the option with a better health condition.

29. Comorbidity
    a) If patient ticked both "Yes" and "No" as answers to the "Comorbidity (Heart disease, Stroke, Lung disease, Diabetes)" questions, then choose "Yes" answer;
    b) If both patient and PCP report "Comorbidity", then use the PCP Data.

30. Ethnicity
    a) If patient didn't report "Ethnicity", then use the information from (in the order of declining priority):
       - "Ethnicity_Other_Details";
       - "Other main language spoken at home";
       - "The main language spoken at home" (only for Victoria);
       - "The main language spoken at home is the chief one for this jurisdiction"="Yes" given "Main language spoken at home is other than the main one for this jurisdiction"="No";
    b) Consider Ethnicity as unknown, if answers to the "Ethnicity" question are multiple and belong to different categories ('white', 'Asian', 'black', 'other').

31. Education

If patient gave multiple answers to the "Education" question, then use the option with a higher level of education.

32. Smoking Current
    a) If patient ticked both "Yes" and "No" as answers to the "Smoking Current" question, then use "Yes" answer;
    b) If patient hasn't ticked neither "Yes" nor "No, then consider this case as Unknown.

33. Smoking Number

If patient reports "SmokingNumber" as text, then re-code using following rules:

a) Where there is a number smoked /day – accept number;
b) Where a range has been given – take the upper value;
c) Where patient has put 10+ or 20+ - capture this as 11 or 21;
d) Where number of cigarettes smoked in the past and currently being smoked are provided - average the numbers;
e) Non entries code as "." ;
f) Non-smokers (eg, "nil", "N/A") are coded as "0".

34. Smoked ever
    a) If patient ticked both "Yes" and "No" as answers to the "Smoking ever" question, then use "Yes" answer;
    b) If patient hasn't ticked neither "Yes" nor "No": consider it as "Yes", if patient is a current smoker ("Smoking_Current="Yes"") or has specified a number of cigarettes ("SmokingNumber">0). Otherwise consider this case as Unknown.
    c) If patient has ticked "No": recode it to "Yes", if patient is a current smoker ("Smoking_Current="Yes").

35. Nature of referral
    a) Review free-text for " Nature of referral" (PCP Data) and re-code, if possible;
    b) In the case of multiple responses, use a single option as (in the order of declining priority):
       - "Referral for immediate admission";
       - "Urgent referral";
       - "Less urgent referral";
       - "General referral" ;
       - "No referral";
       - "Other".

36. Stage-TNM<br>a) If specialist gave multiple responses to the “Stage_TNM” question, then use the highest category;<br>b) If registry gave multiple responses to the “Stage_TNM”, then use a single option (in the order of declining priority):<br>- stage at time of diagnosis<br>- stage at surgery<br>- stage at oncology<br>c) If “Stage_TNM” is reported by both the specialist and registry, then use the registry data;<br>d) If “Stage_TNM” is unknown or “not able to stage”, then use “Stage_Duke”.
37. Stage Dukes<br>a) If specialist gave multiple responses to the “Stage_Dukes’” question, then use the highest category;<br>b) If “Stage_Dukes” is reported by both the specialist and registry, then use the registry data.
