# Supplementary material for: Diagnostic routes and time intervals for patients with colorectal cancer in 10 international jurisdictions; findings from a cross-sectional study from the International Cancer Benchmarking Partnership (ICBP)
Source: BMJ Open. 2018 Nov 27;8(11):e023870. doi: 10.1136/bmjopen-2018-023870 (PMC6278806; doi:10.1136/bmjopen-2018-023870)
Supplement: Supplementary file 5 [file bmjopen-2018-023870supp005.pdf]

Supplementary File 5 – Ethical approvals, recruitment practices, ICBP M4 working group and ARG

Section 1 – Ethical and other approvals obtained in each Module 4 participating jurisdiction

	Date of Ethics Approval	Approvals obtained	Reference
**Victoria**	4 September 2012	Cancer Council Victoria Human Research Ethics Committee	HREC 1125
**Manitoba**	7 March 2013<br><br>15 April 2013	Health Research Ethics Board, University of Manitoba<br><br>Research Resource Ethics Committee, CancerCare Manitoba	HS15227 (H2012:105)<br><br>RRIC#28-2012
**Ontario**	7 November 2013<br>28 January 2014	University of Toronto Research Ethics Board	27881
**Denmark**	6 August 2013<br><br>19 June 2013	The Danish Data Protection Agency<br><br>According to Danish law and the Central Denmark Region Committees on Health Research Ethics, approval by the National Committee on Health Research Ethics was not required as no biomedical intervention was performed.	2013-41-2030<br><br>1-10-72-20-13
**Sweden**	23 October 2013	Ethics Review Board, Uppsala	2013/306
**Norway**	04 April 2013	Regional committees for medical and health research ethics	2013/136/REK nord
**Wales**	16 November 2012	NRES Committee East Midlands – Derby 2, local R&D for each health board	11/EM/0420
**Scotland**	16 November 2012	NRES Committee East Midlands – Derby 2,<br>R&D for each health board,<br>Privacy Advisory Committee,<br>CHI Advisory Group	11/EM/0420
**N Ireland**	1 June 2012	ORECNI Ethical approval, local governance for each health Trust	12/NI/0053
**England**	16 November 2012	NRES Committee East Midlands – Derby 2<br>R&D for each Clinical Research Network	11/EM/0420

Section 2 – Local recruitment practice in each Module 4 participating jurisdiction

	**Recruitment practice variation**
**Victoria**	The relevant healthcare professional confirmed eligibility prior to questionnaire mail-out to patients. Additional patients were recruited (above the required 200 symptomatic CRC patients) to meet the needs of a local study.
**Manitoba**	The data from cancer treatment specialists was not available.
**Ontario**	Additional patients were recruited (above the required 200 symptomatic CRC patients) to meet the needs of a local study.
**Denmark**	The cancer treatment specialist data were completed using clinical databases instead of through a survey.
**Sweden**	Only patients answered the survey – no primary care or cancer treatment specialist data available.
**Norway**	Some patients received and completed their surveys up to 9 months post diagnosis; their data were included (although flagged for subsequent analysis of any resulting sampling bias).
**Wales**	No variation.
**Scotland**	No variation.
**N Ireland**	The cancer treatment specialist data were collected directly from registries instead of through a survey. Some screen-detected cancer patients were excluded in the identification process.
**England**	No variation.

Section 3 – ICBP Module 4 Working Group

Alina Zalounina Falborg, Research Unit for General Practice, Department of Public Health, Aarhus University, Bartholins Allé 2, 8000 Aarhus C, Denmark

Andriana Barisic, Department of Prevention and Cancer Control, Cancer Care Ontario, 620 University Avenue, Toronto, Ontario, M5G 2L7, Canada

Anna Gavin, Northern Ireland Cancer Registry, Centre for Public Health, Queen's University Belfast, Mulhouse Building, Mulhouse Road, Belfast, BT12 6DP, United Kingdom

Anne Kari Knudsen, European Palliative Care Research Centre (PRC), Department of Oncology, Oslo University Hospital and Institute of Clinical Medicine, University of Oslo, Oslo, N-0424 Oslo, Norway

Breann Hawryluk, Department of Patient Navigation, Cancer Care Manitoba, 675 McDermot Street, Winnipeg, Manitoba, MB R3E 0V9, Canada

Chantelle Anandan, Centre for Population Health Sciences, University of Edinburgh, Doorway 1, Medical Quad Teviot Place, Edinburgh, EH8 9DX, United Kingdom

Conan Donnelly, Centre for Public Health, Queen's University Belfast, Mulhouse Building, Mulhouse Road, Belfast, BT12 6DP, United Kingdom

David H Brewster, Scottish Cancer Registry, Information Services Division, NHS National Services Scotland, Gyle Square, 1 South Gyle Crescent, Edinburgh, EH12 9EB, United Kingdom; Centre for Population Health Sciences, University of Edinburgh, Teviot Place, Edinburgh, EH8 9DX, United Kingdom

David Weller, Centre for Population Health Sciences, University of Edinburgh, Doorway 1, Medical Quad Teviot Place, Edinburgh, EH8 9DX, United Kingdom

Donna Turner, Population Oncology, Cancer Care Manitoba, 675 McDermot Street, Winnipeg, Manitoba, MB R3E 0V9, Canada

Elizabeth Harland, Department of Epidemiology and Cancer Registry, CancerCare Manitoba, 675 McDermot Street, Winnipeg, MB R3E 0V9, Manitoba

Eva Grunfeld, Knowledge Translation Research Network Health Services Research Program, Ontario Institute for Cancer Research; Professor and Vice Chair Research Department of Family and Community Medicine, University of Toronto, 500 University Avenue, Toronto, Ontario, M5G 1V7, Canada

Evangelia Ourania Fourkala, Gynaecological Cancer Research Centre, Women's Cancer, Institute for Women's Health, University College London, London, WC1E 6BT, United Kingdom

Henry Jensen, Research Unit for General Practice, Department of Public Health, Aarhus University, Bartholins Allé 2, 8000 Aarhus C, Denmark

Irene Reguilon, International Cancer Benchmarking Partnership, Cancer Research UK, London, EC1V 4AD, United Kingdom

Jackie Boylan, Centre for Public Health, Queen's University Belfast, Mulhouse Building, Mulhouse Road, Belfast, BT12 6DP, United Kingdom

Jacqueline Kelly, Northern Ireland Cancer Registry, Centre for Public Health, Queen’s University Belfast, Mulhouse Building, Mulhouse Road, Belfast, BT12 6DP, United Kingdom

Jatinderpal Kalsi, Gynaecological Cancer Research Centre, Women's Cancer, Institute for Women's Health, University College London, London, WC1E 6BT, United Kingdom

John Butler, The Royal Marsden, Fulham Road, London, SW3 6JJ, United Kingdom

Kerry Moore, Centre for Public Health, Queen's University Belfast, Mulhouse Building, Mulhouse Road, Belfast, BT12 6DP, United Kingdom

Maria Rejmyr Davis, Southern Sweden Regional Cancer Center, Medicon Village, Scheelevägen 8, building 404, 223 81 Lund, Sweden

Martin Malmberg, Department of Oncology, Lund University Hospital, SE-221 85 Lund, Sweden

Mats Lambe, Regional Cancer Center Uppsala and Department of Medical Epidemiology and Biostatics, Karolinska Institutet, SE-171 77 Stockholm, Sweden

Oliver Bucher, Department of Epidemiology and Cancer Registry, CancerCare Manitoba, 675 McDermot Street, Winnipeg, MB R3E 0V9, Manitoba

Peter Vedsted, Research Unit for General Practice, Department of Public Health, Aarhus University, Bartholins Allé 2, 8000 Aarhus C, Denmark

Rebecca-Jane Law, North Wales Centre for Primary Care Research, Bangor University, Cambrian House 2, Wrexham Technology Park, Wrexham, LL13 7YP, United Kingdom

Rebecca Bergin, Centre for Behavioural Research in Cancer, 615 St Kilda Rd, Melbourne, Victoria, 3004, Australia; Department of General Practice, University of Melbourne, 200 Berkeley St, Victoria, 3053, Australia

Richard D Neal, North Wales Centre for Primary Care Research, Bangor University, Cambrian House 2, Wrexham Technology Park, Wrexham, LL13 7YP, United Kingdom; Academic Unit of Primary Care, Leeds Institute of Health Sciences, University of Leeds, Leeds, LS2 9NL, United Kingdom

Samantha Harrison, Early Diagnosis and International Cancer Benchmarking Partnership, Policy and Information, Cancer Research UK, London, EC1V 4AD, United Kingdom

Sigrun Saur Almberg, Department of Cancer Research and Molecular Medicine, Faculty of Medicine, Norwegian University of Science and Technology (NTNU), N-7491 Trondheim, Norway

Therese Kearney, Northern Ireland Cancer Registry, Centre for Public Health, Queen's University Belfast, Mulhouse Building, Mulhouse Road, Belfast, BT12 6DP, United Kingdom

Victoria Cairnduff, Northern Ireland Cancer Registry, Centre for Public Health, Queen's University Belfast, Mulhouse Building, Mulhouse Road, Belfast, BT12 6DP, United Kingdom

Victoria Hammersley, Centre for Population Health Sciences, University of Edinburgh, Doorway 1, Medical Quad Teviot Place, Edinburgh, EH8 9DX, United Kingdom

Victoria White, Centre for Behavioural Research in Cancer, Cancer Council Victoria, 615 St Kilda Road, Melbourne, Victoria, 3004, Australia; School of Psychology Deakin University, Geelong, Victoria, 3217, Australia

Usha Menon, Gynaecological Cancer Research Centre, Women's Cancer, Institute for Women's Health, University College London, London, WC1E 6BT, United Kingdom

Yulan Lin, European Palliative Care Research Centre (PRC), Department of Oncology, Oslo University Hospital and Institute of Clinical Medicine, University of Oslo, Oslo, N-0424 Oslo, Norway

Section 4 – ICBP Module 4 Academic Reference Group:

Prof Jan Willem Coebergh, Professor of Cancer Surveillance, Department of Public Health, Erasmus Universiteit Rotterdam, Rotterdam, the Netherlands

Jon Emery, Professor of Primary Care Cancer Research, University of Melbourne and Clinical Professor of General Practice, University of Western Australia, Australia

Dr Stefan Bergström, Senior consultant oncologist, Department of Oncology, Gävle, Sweden

Dr Monique E van Leerdam, Erasmus MC University Medical Centre, the Netherlands

Prof Marie-Louise Essink-Bot, Academic Medical Centre, Amsterdam University, the Netherlands

Prof Una MacLeod, Senior Lecturer in General Practice and Primary Care, Hull-York Medical School, United Kingdom
