# Supplementary material for: Diagnostic routes and time intervals for patients with colorectal cancer in 10 international jurisdictions; findings from a cross-sectional study from the International Cancer Benchmarking Partnership (ICBP)
Source: BMJ Open. 2018 Nov 27;8(11):e023870. doi: 10.1136/bmjopen-2018-023870 (PMC6278806; doi:10.1136/bmjopen-2018-023870)
Supplement: Supplementary file 6 [file bmjopen-2018-023870supp006.pdf]

Supplementary File 6 – graphs of regression analysis for symptomatic patients (based on Table 6). The difference in the length of jurisdiction's intervals are shown compared to the reference Wales (days).

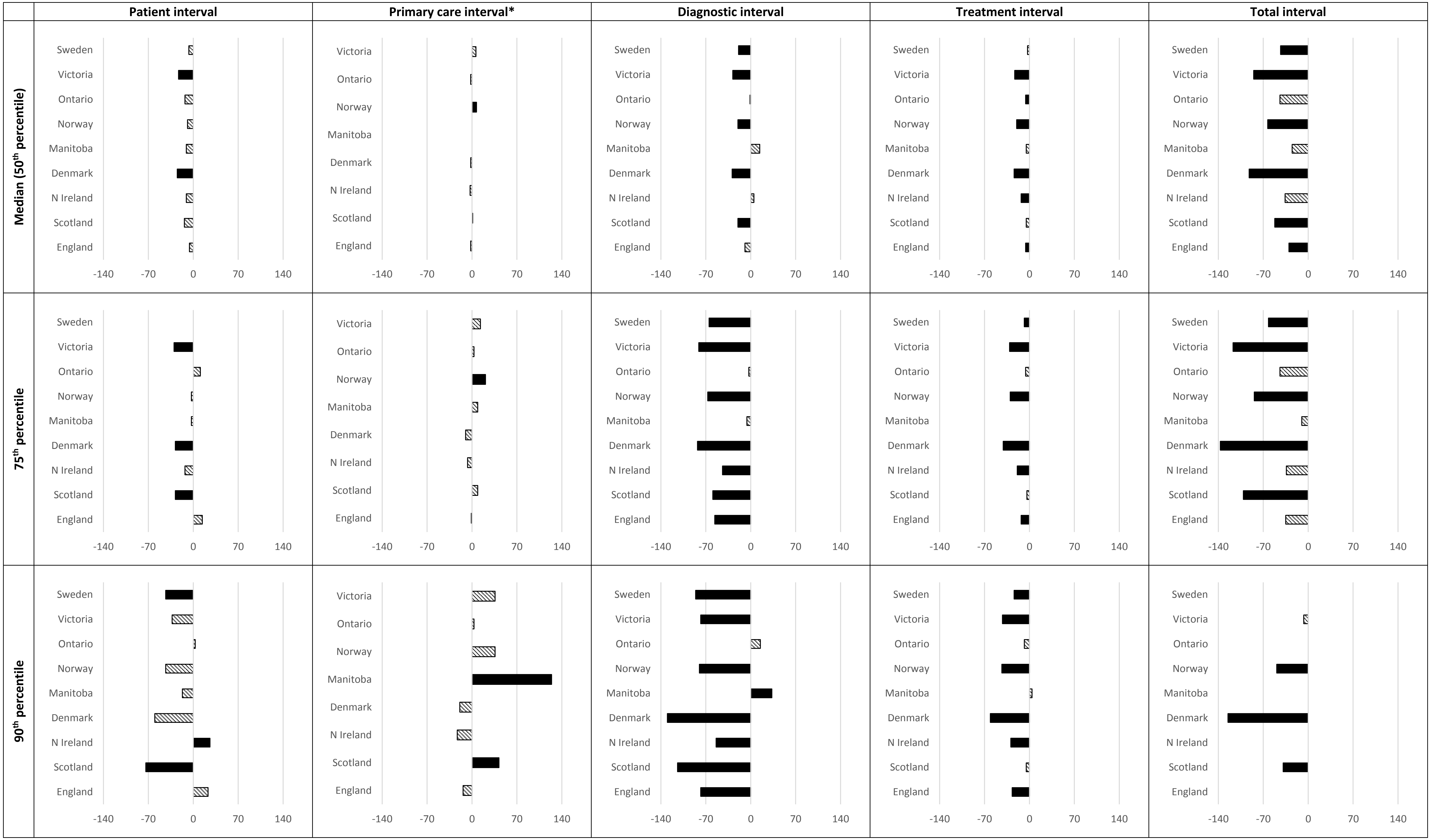

* Sweden did not provide any data for the primary care interval, and so has not been included in these graphs.

Differences in interval lengths (in days) are shown for the median, 75th and 90th percentiles compared to the reference used for the regression analyses, Wales. Wales is represented by the axis, with jurisdictions with shorter intervals shown to the left of the axis, and jurisdictions with longer intervals shown to the right of the axis for each graph. Statistically significant results are shown in solid bars, whilst non-significant results are shown with a pattern fill.
