# Supplementary material for: Diagnostic routes and time intervals for patients with colorectal cancer in 10 international jurisdictions; findings from a cross-sectional study from the International Cancer Benchmarking Partnership (ICBP)
Source: BMJ Open. 2018 Nov 27;8(11):e023870. doi: 10.1136/bmjopen-2018-023870 (PMC6278806; doi:10.1136/bmjopen-2018-023870)
Supplement: Supplementary file 2 [file bmjopen-2018-023870supp002.pdf]

ICBP
International Cancer
Benchmarking Partnership

International Cancer Benchmarking Partnership Module 4

Patient questionnaire
Colorectal Cancer

Thank you very much for taking the time to fill in this questionnaire – it should take about 20 minutes to complete. We are sending the questionnaire to a large sample of people who we understand have had a diagnosis of colorectal cancer. If this has been sent to you in error and you do not have cancer, please do not continue and return the documents in the prepaid envelope.

Sample

Our aim is to gain a better understanding of the process by which people have their cancer diagnosed. We would also like to find out more about the symptoms they experience (if any), and the pathway they follow from start of symptoms to treatment of their cancer. This will help in identifying ways in which cancers can be diagnosed quickly and effectively. Thank you once again for your time.

This information is confidential and will not be passed to anyone involved in your treatment.

Name: ______________________

Date of Birth: ______________________

Address: ______________________

____

____

Copyright property of the International Benchmarking Partnership (ICBP)
and Module 4 collaborators. Usage must be authorised

ICBP
International Cancer
Benchmarking Partnership

Consent form

Please read the consent form and sign your name and date **BELOW**.

If you require any clarification, please do not hesitate to ring the study team members. Their contact details are found on the information sheet.

Please be reassured that your responses are completely confidential and will not be passed to anyone involved in your treatment. For the purposes of the study it is important that you agree to consent to all the statements listed below.

- I confirm that I have read the attached information sheet and I understand why the research is being done.
- I am willing for the team to request information from my GP and hospital doctors which is relevant to the audit as described in the information sheet.
- I give permission for my details (name, address) to be given to the cancer registry (NHS Information Centre for Health and Social Care) for follow up.
- I agree for the information I have provided and any other relevant information from my medical records to be stored as described in the information sheet under the custodianship of University College London.
- I consent to sharing of coded data which contains no personal identifiers between researchers, some of whom are located outside the European Union.
- I consent for use of my data if I become mentally incapacitated during the course of the project.

I agree to all the statements listed and consent to participate in the study.

Sample

Name (Please print)

Signature: Date:

If we have any questions, may we phone you for clarification?
(Please tick) ☐ **Yes** ☐ **No**

If **Yes**, please provide your telephone number:

Copyright property of the International Benchmarking Partnership (ICBP)
and Module 4 collaborators. Usage must be authorised

ICBP
International Cancer
Benchmarking Partnership

1. **Please can you confirm the details of your GP/GP practice (name, practice address – as best as you can remember): We appreciate that you may have more than one GP involved in your care – in which case, we are interested in the GP you would say provides the majority of your care, particularly relating to the cancer you've had diagnosed.**

Name of doctor ______________________________

Name of practice ______________________________

Address ______________________________

____

____

Postcode ______________________________

Town ______________________________

Sample

Copyright property of the International Benchmarking Partnership (ICBP)
and Module 4 collaborators. Usage must be authorised

ICBP
International Cancer
Benchmarking Partnership

2. Which of the following best describes the events which led to your diagnosis of cancer? (please tick only **ONE** answer)

	✓
I had symptoms/I noticed a bodily change and went to see a doctor (e.g. GP)	
I had symptoms/I noticed a bodily change and went/was taken to Accident and Emergency (A&E)	
I had seen a doctor/GP with symptoms, but went/was taken to Accident and Emergency (A&E) when things worsened	
I was being investigated by my doctor(s) for another problem during which time the cancer was discovered	
I had a cancer screening test as part of a colorectal screening programme (e.g. the NHS Bowel Cancer Screening Programme in England – NHS BCSP)	
Other (please describe):	

Sample

What date did you have this screening test? If you cannot remember the exact date, you can fill in the month and the year.

Day (optional), month, year

D	D	M	M	Y	Y	Y	Y

Other (please describe):

Copyright property of the International Benchmarking Partnership (ICBP)
and Module 4 collaborators. Usage must be authorised

ICBP
International Cancer
Benchmarking Partnership

3. The following health concerns or symptoms are commonly experienced with colorectal cancer.

Rectal bleeding or blood in faeces (poo)
Change in bowel habit
Unexplained tiredness/fatigue
Unexplained weight loss
Pelvic or abdominal pain
Skin went yellow
Abdominal distension/increased abdominal size/persistent bloating

Sample

Please write down ALL health concern(s) or symptom(s) you may have had before contacting a doctor or taking part in screening. It does not matter if they are not included in the list above:

Please write your health concern(s) or symptom(s) in the boxes below:
1)
2)
3)
4)
5)
6)

	✓
This is not applicable to me (e.g. I did not have any symptoms), please tick	

Copyright property of the International Benchmarking Partnership (ICBP)
and Module 4 collaborators. Usage must be authorised

ICBP
International Cancer
Benchmarking Partnership

4. Please write down your best estimate of the date you noticed the first of these health concern(s) or symptom(s). If you cannot remember the exact date, you can fill in the month and the year.

Day (optional), month, year

D	D	M	M	Y	Y	Y	Y

This is not applicable to me (e.g. I had no symptoms), please tick	

5. Approximately how long did you have health concern(s) or symptom(s) before contacting a doctor? (Please think of the first visit to the doctor, not re-visits after that). Please tick only **ONE** answer.

Less than 1 week	
1-2 weeks	
3-4 weeks	
5-7 weeks	
2-5 months	
6-12 months	
More than 12 months	

This is not applicable to me (e.g. I had no symptoms), please tick	

Sample

Copyright property of the International Benchmarking Partnership (ICBP)
and Module 4 collaborators. Usage must be authorised

ICBP
International Cancer
Benchmarking Partnership

6a. Once you contacted a practice about your health concern(s) or symptom(s), how long did it take to get an appointment with a doctor? (Please think of the first visit to the doctor, to discuss your health concern(s) or symptom(s)). Please tick only **ONE** answer.

	✓
Same day/next day	
Within 1 week	
1-2 weeks	
3-4 weeks	
Longer	

If there was no waiting time<br>(e.g. you went/were taken to A&E), please tick this box	

This is not applicable to me (e.g. I had no symptoms), please tick	

Sample

6b. What was the date you first saw your doctor about your health concern(s) or symptom(s)? If you cannot remember the exact date, you can fill in the month and the year.

Day (optional), month, year

D	D	M	M	Y	Y	Y	Y

	✓
This is not applicable to me (e.g. I had no symptoms), please tick	

Copyright property of the International Benchmarking Partnership (ICBP)
and Module 4 collaborators. Usage must be authorised

ICBP
International Cancer
Benchmarking Partnership

7. How many times did you visit the following for the investigation of your symptoms before your cancer was diagnosed?

	Please write down the number of visits
GP	
Hospital	
Consultant/specialist outside of a hospital	

	✓
This is not applicable to me (e.g. I had no symptoms)	

8a. After your doctor referred you to a specialist, how long did it take you to get an appointment? Please tick only **ONE** answer.

	✓
Less than 1 week	
1-2 weeks	
3-4 weeks	
5-7 weeks	
2-5 months	
6-12 months	
More than 12 months	

	✓
This is not applicable to me (eg my doctor did not refer me), please tick	

Sample

Copyright property of the International Benchmarking Partnership (ICBP)
and Module 4 collaborators. Usage must be authorised

International Cancer Benchmarking Partnership

8b. What was the date of your first appointment with a doctor, involved in investigating and/or treating your cancer, to whom you were referred? If you cannot remember the exact date, you can fill in the month and the year.

Day (optional), month, year

D	D	M	M	Y	Y	Y	Y

This is not applicable to me (e.g. my doctor did not refer me), please tick	✓

9. What was the date you were told you had cancer? If you cannot remember the exact date, you can fill in the month and the year.

Day (optional), month, year

D	D	M	M	Y	Y	Y	Y

Sample

Copyright property of the International Benchmarking Partnership (ICBP) and Module 4 collaborators. Usage must be authorised

ICBP
International Cancer
Benchmarking Partnership

10. Have you had any of the following treatments for your cancer yet? If so, please can you estimate the date this treatment started? Please tick **ALL** that apply. If you cannot remember the exact date, you can fill in the month and the year.

	**Type of treatment**		**Date of treatment** (give first date if you had more than one)
a.	Surgery	☐ **Yes** ☐ **No**	Day (optional), month, year: D D M M Y Y Y Y
b.	Chemotherapy	☐ **Yes** ☐ **No**	Day (optional), month, year: D D M M Y Y Y Y
c.	Radiotherapy	☐ **Yes** ☐ **No**	Day (optional), month, year: D D M M Y Y Y Y
d.	Other Please specify:	☐ **Yes** ☐ **No**	Day (optional), month, year: D D M M Y Y Y Y
e.	Treatment not started yet	☐ **Yes**	

Sample

Copyright property of the International Benchmarking Partnership (ICBP) and Module 4 collaborators. Usage must be authorised

ICBP
International Cancer
Benchmarking Partnership

11. Who is the consultant doctor who has taken responsibility for diagnosing and or/treating your cancer?

Name of consultant:
Hospital name:
Hospital department:

Please can you answer some more general questions about your health?
It will help us in interpreting your responses to this questionnaire to know about your general health and other health problems you may have had in the past.

12. Looking back to the 2 years before you were diagnosed with cancer, would you say your general health was (Please tick only **ONE** answer.)**:** ✓

Sample

Very good	
Good	
Fair	
Poor	
Very poor	

13. Have you been treated before for any of the conditions below?
Please tick 'yes' or 'no' for each condition:

Heart disease	☐ **Yes**	☐ **No**
Stroke	☐ **Yes**	☐ **No**
Lung disease	☐ **Yes**	☐ **No**
Diabetes	☐ **Yes**	☐ **No**

Copyright property of the International Benchmarking Partnership (ICBP)
and Module 4 collaborators. Usage must be authorised

ICBP
International Cancer
Benchmarking Partnership

Finally, a little more information about you. The information you provide below will help us to analyse the results of the survey in more detail.

14. Which of these best describes your ethnic group? (please tick one box, as appropriate). If you are descended from more than one ethnic or racial group, please tick the group you consider you belong to, or tick 'any other ethnic group'.

	✓		✓		✓		✓
White		Chinese		Black - Caribbean		Black - African	
Black - other		Indian		Pakistani		Bangladeshi	
Any other ethnic group, please specify							

Sample

15. What is the main language spoken in your home? Please tick

	✓
English	
Other, please specify:	

16. What is the highest level of education you have achieved?
Please tick only **ONE** answer.

	✓
Finished school at or before the age of fifteen	
Completed GCSEs, O-levels or equivalent	
Completed A Levels or equivalent	
Completed further education but not a degree	
Completed a Bachelor's degree / Masters degree / PhD	
Other, please specify:	

Copyright property of the International Benchmarking Partnership (ICBP)
and Module 4 collaborators. Usage must be authorised

ICBP
International Cancer
Benchmarking Partnership

17. Have you ever smoked cigarettes, including hand-rolled ones, pipes or cigars?

☐ Yes ☐ No

18. Are you a current smoker, smoking either cigarettes, including hand-rolled ones, pipes or cigars?

☐ Yes ☐ No

19. If you are a current smoker or have smoked in the past, how many cigarettes, including hand-rolled ones, pipes or cigars on average do you smoke/have you smoked per day?

Number per day:	

Sample

Copyright property of the International Benchmarking Partnership (ICBP)
and Module 4 collaborators. Usage must be authorised

ICBP
International Cancer
Benchmarking Partnership

20. Further comments

Please add anything else that you would like to tell us about your cancer diagnosis or treatment.

Sample

Thank you very much for taking the time to complete this questionnaire.

Copyright property of the International Benchmarking Partnership (ICBP)
and Module 4 collaborators. Usage must be authorised
