# Supplementary material for: Diagnostic routes and time intervals for patients with colorectal cancer in 10 international jurisdictions; findings from a cross-sectional study from the International Cancer Benchmarking Partnership (ICBP)
Source: BMJ Open. 2018 Nov 27;8(11):e023870. doi: 10.1136/bmjopen-2018-023870 (PMC6278806; doi:10.1136/bmjopen-2018-023870)
Supplement: Supplementary file 3 [file bmjopen-2018-023870supp003.pdf]

ICBP
International Cancer
Benchmarking Partnership

International Cancer Benchmarking Partnership Module 4

Primary Care Audit
Colorectal Cancer

Thank you very much for agreeing to fill in this questionnaire. As part of an international study examining differences in cancer survival, we are sending the questionnaire to health care providers of a sample of consented patients with cancer.

Our aim is to gain a better understanding of the process by which people have their cancer diagnosed – the symptoms they experience, and the pathway they follow from onset of symptoms to treatment of their cancer. This will help in identifying ways in which cancers can be diagnosed and treated quickly and effectively. Thank you once again for your time.

Sample

Please can you refer to your patient's notes in completing the questionnaire as this will help in obtaining accurate data on time points.

If you would prefer to return this questionnaire without the patient details, please tear off along the dotted line

ID-number: Jurisdiction-ID + Patient-ID:

Copyright property of the International Benchmarking Partnership (ICBP)
and Module 4 collaborators. Usage must be authorised

ICBP
International Cancer
Benchmarking Partnership

Sample

Patient information

ID-number: Jurisdiction-ID + Patient-ID:

Full name:

Address:

Postcode:

Date of birth: D D M M Y Y Y Y

Copyright property of the International Benchmarking Partnership (ICBP)
and Module 4 collaborators. Usage must be authorised

International Cancer Benchmarking Partnership

1. Duration of symptoms

Please estimate how long your patient had symptom(s), attributable to colorectal cancer, before attending your practice (or other health service).

We appreciate that identifying a 'date of first symptom' is not always straightforward – particularly when there are multiple and/or chronic symptoms. Nevertheless, we hope you can provide a 'best estimate'.

**Estimate of symptom duration** (please tick **one**):	✓	**What were the symptoms?** Please describe:
Less than 1 week		
1 to 4 weeks		
5 to 7 weeks		
2-5 months		
6-12 months		
More than 12 months		
Not possible to estimate		
No symptoms (e.g. screen detected cancers)		

Sample

Copyright property of the International Benchmarking Partnership (ICBP)
and Module 4 collaborators. Usage must be authorised

International Cancer Benchmarking Partnership

2. Pathway of presentation

2.1 Through what route did the patient first present? Please tick **ONE**.

	✓	
Your patient first presented to primary care (either in-hours or out-of-hours)		Please can you provide your best approximation of the date of this primary care visit<br>D D M M Y Y Y Y
Your patient presented straight to A&E (with or without your involvement)		
Your patient first presented to primary care, but then at a later date presented to A&E as an emergency (with or without your involvement)		Please can you provide your best approximation of the date of this primary care visit<br>D D M M Y Y Y Y
Your patient's colorectal cancer was diagnosed through an organised screening programme (e.g. not as a result of investigation of symptoms)		
Other – please describe:		

Sample

Copyright property of the International Benchmarking Partnership (ICBP)
and Module 4 collaborators. Usage must be authorised

ICBP
International Cancer
Benchmarking Partnership

3. Date you ordered any tests/investigations in response to symptom(s).

We are interested in any kind of tests/investigations (e.g. imaging etc) that you may have ordered. Please only consider the tests/investigations that you ordered yourself. Please tick **ALL** that apply and put in the date that the test/investigation was ordered:

	✓	
Blood test		D D M M Y Y Y Y
Faecal occult blood test (FOBT)		D D M M Y Y Y Y
Colonoscopy		D D M M Y Y Y Y
Sigmoidoscopy		D D M M Y Y Y Y
Double contrast barium enema (DCBE)		D D M M Y Y Y Y
Digital Rectal Exam (DRE)		D D M M Y Y Y Y
Virtual colonoscopy (computerised tomographic colonography)		D D M M Y Y Y Y
Other (please specify):		D D M M Y Y Y Y

Sample

4. Date of referral to specialist medical services

At what date did you **first** refer the patient to hospital or another specialist transferring the responsibility for on-going investigation/treatment to other medical services?

D	D	M	M	Y	Y	Y	Y

Copyright property of the International Benchmarking Partnership (ICBP)
and Module 4 collaborators. Usage must be authorised

ICBP
International Cancer
Benchmarking Partnership

5. Nature of this referral

5.1 Do you know the date that the patient was seen for this referral?

☐ **Yes**, please provide the date: D D M M Y Y Y Y

☐ **No**

5.2 If you did make a referral to specialist services, which of the following best describes the nature/characteristics of this referral? Please tick **one**.

	✓
Emergency admission: a referral to A&E (or equivalent) for immediate admission	
An urgent referral for assessment of cancer symptoms/signs/test results (Note this will be within 2 weeks for England/Wales)	
A less urgent referral in which cancer is raised as a possibility (Note this will be greater than 2 weeks for England/Wales)	
A more general referral for investigation and assessment without cancer mentioned	
No referral was made	
Other – please describe	

Sample

5.3 Would you say this patient's diagnostic pathway was conducted predominantly in the public or private system? Please tick **one**.

	✓
Public healthcare system	
Private healthcare system	

Copyright property of the International Benchmarking Partnership (ICBP)
and Module 4 collaborators. Usage must be authorised

International Cancer
Benchmarking Partnership

6. Date of colorectal cancer diagnosis

This can be decided in different ways. Please provide whichever of the following dates you have to hand. Please tick **all** that apply.

	✓	
Date of histological confirmation [ideal]		D D M M Y Y Y Y
Date results of investigation (histological or other) confirming cancer received		D D M M Y Y Y Y
Date patient was told		D D M M Y Y Y Y
Date biopsy undertaken		D D M M Y Y Y Y
Date patient was first admitted to hospital because of the malignancy		D D M M Y Y Y Y
Other (please specify)		D D M M Y Y Y Y

Sample

Copyright property of the International Benchmarking Partnership (ICBP)
and Module 4 collaborators. Usage must be authorised

ICBP
International Cancer
Benchmarking Partnership

7. Additional information

Finally, we are interested to know what other conditions your patient has, and the severity/impact of these conditions

Have you and/or any of your partners treated this patient (or has the patient been to hospital) for any of the following conditions? Please tick **all** that apply:

Cardiovascular disease	☐ **Yes** ☐ **No**
Stroke	☐ **Yes** ☐ **No**
Lung disease	☐ **Yes** ☐ **No**
Diabetes	☐ **Yes** ☐ **No**

Sample

Are there any other comments you would like to make about this patient?

Name (and title):

Signature:

Date:

Thank you very much for taking the time to complete this questionnaire.

Copyright property of the International Benchmarking Partnership (ICBP)
and Module 4 collaborators. Usage must be authorised
