# Supplementary material for: Diagnostic routes and time intervals for patients with colorectal cancer in 10 international jurisdictions; findings from a cross-sectional study from the International Cancer Benchmarking Partnership (ICBP)
Source: BMJ Open. 2018 Nov 27;8(11):e023870. doi: 10.1136/bmjopen-2018-023870 (PMC6278806; doi:10.1136/bmjopen-2018-023870)
Supplement: Supplementary file 4 [file bmjopen-2018-023870supp004.pdf]

ICBP
International Cancer
Benchmarking Partnership

International Cancer Benchmarking Partnership Module 4

Specialist Care Audit
Colorectal Cancer

Thank you very much for agreeing to fill in this questionnaire – it should take about 10 minutes to complete. As part of an international study examining differences in cancer survival, we are sending the questionnaire to health care providers of a sample of patients with cancer.

Our aim is to gain a better understanding of the process by which people have their cancer diagnosed – the symptoms they experience, and the pathway they follow from onset of symptoms to treatment of their cancer. We hope you can help us with information on this patient's cancer journey once they were referred to specialist cancer services. This will help in identifying ways in which cancers can be diagnosed and treated quickly and effectively.

Sample

Thank you once again for your time

Please can you refer to your patient's notes in completing the questionnaire, as this will help in obtaining accurate data on time points.

If you would prefer to return this questionnaire without the patient details, please tear off along the dotted line.

Your patient ______________________________

is participating in the study.

Copyright property of the International Benchmarking Partnership (ICBP)
and Module 4 collaborators. Usage must be authorised

ICBP
International Cancer
Benchmarking Partnership

Sample

Patient information

ID-number: Jurisdiction-ID + Patient-ID:

Full name:

Address:

Postcode:

Date of birth: D D M M Y Y Y Y

Copyright property of the International Benchmarking Partnership (ICBP)
and Module 4 collaborators. Usage must be authorised

ICBP
International Cancer
Benchmarking Partnership

1. **Date patient first attended hospital/specialist services related to their cancer diagnosis.** We appreciate this date can at times be difficult to identify, particularly when there have been multiple visits in the lead up to a definitive diagnosis. Put another way, it's the date that the hospital/specialist service assumed responsibility for on-going investigation/treatment for your patient.

Day (optional), month, year

D	D	M	M	Y	Y	Y	Y

2. **How was the patient referred to the hospital/specialist services related to their cancer diagnosis?** Please tick.

Was it through a:

	✓		✓
GP referral		Screening	
Referral from general surgery clinic		Medical specialist/ Consultant referral	
Other referral – please specify:			

Sample

3. **Where did this first contact/appointment happen?** Please tick.
Which of the following best describes where this first contact/appointment took place?

	✓		✓
Emergency department ('A&E')		Medical outpatient department, please specify which department	
Oncology general outpatient department		Surgical outpatient department, please specify which department	
Other – please specify:			

Copyright property of the International Benchmarking Partnership (ICBP)
and Module 4 collaborators. Usage must be authorised

ICBP
International Cancer
Benchmarking Partnership

4. Date of diagnosis

This can be decided in different ways.
Please tick and complete as many of the following dates as possible.

	✓	
Date of histological confirmation (ideal)		Day (optional), month, year<br>D D M M Y Y Y Y
Date results of investigation confirming cancer received		Day (optional), month, year<br>D D M M Y Y Y Y
Date patient was told		Day (optional), month, year<br>D D M M Y Y Y Y
Date of biopsy		Day (optional), month, year<br>D D M M Y Y Y Y
Date patient was first admitted to hospital because of the malignancy		Day (optional), month, year<br>D D M M Y Y Y Y
Date of MDT confirmation of diagnosis		Day (optional), month, year<br>D D M M Y Y Y Y
Other (please specify):		Day (optional), month, year<br>D D M M Y Y Y Y

Sample

Copyright property of the International Benchmarking Partnership (ICBP)
and Module 4 collaborators. Usage must be authorised

5. Date treatment for the cancer commenced

Based on your records, when would you say that any treatment specifically targeting the patient's cancer started?

Day (optional), month, year

D	D	M	M	Y	Y	Y	Y

6. Additional information

Please can you provide any further information on the patient's cancer:

**TNM**, please tick as appropriate:		**Duke's**, please tick as appropriate:	
0		A	
I		B	
IIA		C	
IIB		D	
IIC			
IIIA			
IIIB			
IIIC			
IV			
Not able to stage			

Sample

Copyright property of the International Benchmarking Partnership (ICBP)
and Module 4 collaborators. Usage must be authorised

ICBP
International Cancer
Benchmarking Partnership

6.1 Histological subtype:

	✓
Adenocarcinoma	
Mucinous (colloid) adenocarcinoma	
Signet-ring cell carcinoma	
Other (please specify):	

Sample

Copyright property of the International Benchmarking Partnership (ICBP)
and Module 4 collaborators. Usage must be authorised

ICBP
International Cancer
Benchmarking Partnership

Further comments

Sample

Copyright property of the International Benchmarking Partnership (ICBP)
and Module 4 collaborators. Usage must be authorised

ICBP
International Cancer
Benchmarking Partnership

Name (and title):

Signature:

Date:

Sample

Are you a ... (please tick below):

	✓
Surgeon	
Medical Oncologist	
Clinical Oncologist	
Clinical Nurse Specialist	
Other (please specify):	

Thank you very much for taking the time to complete this questionnaire.

Copyright property of the International Benchmarking Partnership (ICBP)
and Module 4 collaborators. Usage must be authorised
