# Supplementary material for: Diagnostic routes and time intervals for patients with colorectal cancer in 10 international jurisdictions; findings from a cross-sectional study from the International Cancer Benchmarking Partnership (ICBP)
Source: BMJ Open. 2018 Nov 27;8(11):e023870. doi: 10.1136/bmjopen-2018-023870 (PMC6278806; doi:10.1136/bmjopen-2018-023870)
Supplement: Author's manuscript [file bmjopen-2018-023870.draft_revisions.pdf]

BMJ Open is committed to open peer review. As part of this commitment we make the peer review history of every article we publish publicly available.

When an article is published we post the peer reviewers' comments and the authors' responses online. We also post the versions of the paper that were used during peer review. These are the versions that the peer review comments apply to.

The versions of the paper that follow are the versions that were submitted during the peer review process. They are not the versions of record or the final published versions. They should not be cited or distributed as the published version of this manuscript.

BMJ Open is an open access journal and the full, final, typeset and author-corrected version of record of the manuscript is available on our site with no access controls, subscription charges or pay-per-view fees (http://bmjopen.bmj.com).

If you have any questions on BMJ Open's open peer review process please email info.bmjopen@bmj.com

Diagnostic routes and time intervals for colorectal cancer in 10 international jurisdictions; findings from the International Cancer Benchmarking Partnership (ICBP)

Journal:	*BMJ Open*
Manuscript ID	bmjopen-2018-023870
Article Type:	Research
Date Submitted by the Author:	27-Apr-2018

Complete List of Authors:	Weller, David; University of Edinburgh, Centre for Population Health Sciences<br>Vedsted, Peter; Research Unit for General Practice, Department of Public Health, Aarhus University<br>Zalounina Falborg, Alina; Research Unit for General Practice, Department of Public Health, Aarhus University<br>Jensen, Henry; Research Unit for General Practice, Department of Public Health, Aarhus University<br>Menon, Usha; University College London,<br>Bergin, Rebecca; Cancer Council Victoria, Centre for Behavioral Research in Cancer; University of Melbourne, Department of General Practice<br>Gavin, Anna; Queen's University Belfast, Northern Ireland Cancer Registry<br>Barisic, Andriana; Cancer Care Ontario, Renal Network<br>Lambe, Mats; Karolinska Institutet<br>Neal, RD; Bangor University, North Wales Centre for Primary Care Research; University of Leeds, Academic Unit of Primary Care<br>Turner, Donna<br>White, Victoria; Cancer Council Victoria, Centre for Behavioral Research in Cancer; Deakin University School of Psychology<br>Law, Rebecca-Jane; Bangor University, North Wales Centre for Primary Care Research<br>Lin, Yulan; Oslo University Hospital , European Palliative Care Research Centre (PRC), Department of Oncology<br>Malmberg, Martin; Skanes universitetssjukhus Lund, Department of Oncology<br>Harland, Elizabeth; CancerCare Manitoba, Department of Epidemiology and Cancer Registry<br>Grunfeld, Eva; University of Toronto, Department of Family and Community Medicine<br>Cairnduff, Victoria; Northern Ireland Cancer Registry<br>Brewster, David; University of Edinburgh, Centre for Population Health Sciences; NHS National Services Scotland, Scottish Cancer Registry, Information Services Division<br>Reguilon, Irene; Cancer Research UK, Policy and Information<br>Harrison, Samantha; Cancer Research UK, Policy and Information<br>Working Group, ICBP; Cancer Research UK, Policy and Information

Keywords:	Health policy < HEALTH SERVICES ADMINISTRATION & MANAGEMENT, Organisation of health services < HEALTH SERVICES ADMINISTRATION & MANAGEMENT, International health services < HEALTH SERVICES

	ADMINISTRATION & MANAGEMENT, Gastrointestinal tumours < ONCOLOGY, Epidemiology < ONCOLOGY

SCHOLARONE™ Manuscripts

For peer review only

Diagnostic routes and time intervals for colorectal cancer in 10 international jurisdictions; findings from the International Cancer Benchmarking Partnership (ICBP)

Corresponding author:
David Weller
University of Edinburgh, Medical Quad, Teviot Row, Edinburgh EH8 9DX
david.weller@ed.ac.uk
+44 131 6509518

- David Weller*, Centre for Population Health Sciences, University of Edinburgh, Edinburgh, United Kingdom
- Usha Menon*, Institute for Women's Health, University College London, London, United Kingdom
- Alina Zalounina Falborg, Research Unit for General Practice, Aarhus University, Aarhus, Denmark
- Henry Jensen, Research Unit for General Practice, Aarhus University, Aarhus, Denmark
- Andriana Barisic, Ontario Renal Network, Cancer Care Ontario, Toronto, Canada
- Anne Kari Knudsen, European Palliative Care Research Centre (PRC), Department of Oncology, Oslo University Hospital and Institute of Clinical Medicine, University of Oslo, Oslo, N-0424 Oslo, Norway
- Rebecca J Bergin, Centre for Behavioural Research in Cancer, Cancer Council Victoria, Victoria, Australia; Department of General Practice, University of Melbourne, 200 Berkeley St, Victoria, 3053, Australia
- David H Brewster, Scottish Cancer Registry, Information Services Division, NHS National Services Scotland, Gyle Square, 1 South Gyle Crescent, Edinburgh, EH12 9EB, United Kingdom; Centre for Population Health Sciences, University of Edinburgh, Edinburgh, United Kingdom
- Victoria Cairnduff, Northern Ireland Cancer Registry, Queen's University Belfast, Belfast, United Kingdom
- Anna T Gavin, Northern Ireland Cancer Registry, Queen's University Belfast, Belfast, United Kingdom
- Eva Grunfeld, Health Services Research Program, Ontario Institute for Cancer Research, Toronto, Canada
- Elizabeth Harland, Department of Epidemiology and Cancer Registry, CancerCare Manitoba, Winnipeg, Canada
- Jatinderpal Kalsi, Project Manager, Gynaecological Cancer Research Centre, Women's Cancer, Institute for Women's Health, University College London, United Kingdom
- Mats Lambe, Regional Cancer Center Uppsala and Department of Medical Epidemiology and Biostatics, Karolinska Institutet, Stockholm, Sweden
- Rebecca-Jane Law, North Wales Centre for Primary Care Research, Bangor University, Wrexham, United Kingdom
- Yulan Lin, European Palliative Care Research Centre (PRC), Department of Oncology, Oslo University Hospital and Institute of Clinical Medicine, University of Oslo, Oslo, N-0424 Oslo, Norway
- Martin Malmberg, Department of Oncology, Lund University Hospital, Lund, Sweden
- Donna Turner, Population Oncology, Cancer Care Manitoba, Winnipeg, Canada
- Richard D Neal, North Wales Centre for Primary Care Research, Bangor University, Cambrian House 2, Wrexham Technology Park, Wrexham, LL13 7YP, United Kingdom; Academic Unit of Primary Care, University of Leeds, Leeds, United Kingdom
- Victoria White, Centre for Behavioral Research in Cancer, Cancer Council Victoria, Victoria, Australia; School of Psychology Deakin University, Geelong, Victoria, 3217, Australia
- Samantha Harrison, Policy and Information, Cancer Research UK, London, United Kingdom
- Irene Reguilon, Policy and Information, Cancer Research UK, London, United Kingdom
- ICBP Module 4 Working Group (details within Supplementary File 5)
- Peter Vedsted*, Research Unit for General Practice, Aarhus University, Aarhus, Denmark

For peer review only

For peer review only - http://bmjopen.bmj.com/site/about/guidelines.xhtml

* Equal contribution

Word count: 4,319

Keywords: colorectal cancer, routes to diagnosis, time intervals, international health systems, symptomatic presentation

For peer review only

For peer review only - http://bmjopen.bmj.com/site/about/guidelines.xhtml

ABSTRACT

Objective: International differences in colorectal cancer (CRC) survival and stage at diagnosis have been reported previously. They may be linked to differences in time intervals and routes to diagnosis. The International Cancer Benchmarking Partnership Module 4 (ICBP M4) reports the first international comparison of routes to diagnosis for CRC patients and the time intervals from symptom onset until the start of treatment. Data came from patients in ten jurisdictions across six countries (Canada, the UK, Norway, Sweden, Denmark and Australia).

Design: CRC patients were identified via cancer registries. Data on symptomatic and screened patients were collected; questionnaire data from patients' primary care physicians and specialists, as well as information from treatment records or databases, supplemented patient data from the questionnaires. Routes to diagnosis and the key time intervals were described, as were between-jurisdiction differences in time intervals, using quantile regression.

Participants: A total of 14,664 eligible CRC patients diagnosed between 2013 and 2015 were identified, of which 2,866 were included in the analyses.

Primary and secondary outcome measures: Interval lengths in days (primary), reported patient symptoms (secondary).

Results: The main route to diagnosis for patients was symptomatic presentation and the most commonly reported symptom was 'bleeding/blood in stool'. The median intervals between jurisictions ranged from: 21 to 49 days (patient); 0 to 12 days (primary care); 27 to 76 days (diagnostic); and 77 to 168 days (total, from first symptom to treatment start). Including screen-detected cases did not significantly alter the overall results.

Conclusion: ICBP M4 demonstrates important differences in time intervals between ten jurisdictions internationally. The differences may justify efforts to reduce intervals in some jurisdictions.

Strengths and limitations of this study

- This is the first international study of this scale to use standarised survey methods to systematically examine key intervals from patients first noticing symptoms or bodily changes until the start of treatment for their colorectal cancer
- Questionnaire data were enriched and validated with registry data (cancer registry and screening programmes) and data rules were applied consistentlyto ensure validity
- As with all questionnaire based studies, there may be some response differences due to participant interpretation, cohort characteristics and sampling strategy, but we did not find obvious differences between study participatnts which could bias our results.
- While our analyses adjusted for age, gender and comorbidity, we were unable to adjust for ethnicity and education due to different classification systems in participating countries
- Understanding variations in diagnostic and treatment intervals for colorectal cancer patients may, in jurisdictions with longer intervals, signal the need for improvements in service configuration and patient pathways.

For peer review only - http://bmjopen.bmj.com/site/about/guidelines.xhtml

For peer review only

BACKGROUND

Colorectal cancer (CRC) is a leading cause of morbidity and mortality and places a major burden on health systems; worldwide 1.36 million new cases are diagnosed every year.[1] CRC is the second most common cause of death due to cancer in Europe, accounting for more than 200,000 deaths per year.[2] Prognosis strongly depends on stage at diagnosis, and the disease can mostly be cured if diagnosed at an early stage. Survival has increased over the last several years in Europe.[3] However, there remains substantial international variation in both 1 and 5 year survival, with countries such as the United Kingdom and Denmark having significantly poorer survival than other countries such as Sweden, Canada and Australia (Figure 1).[4] Some of the variation derives from differences in stage at diagnosis which in turn is a result of the pathway to diagnosis and treatment.[5] Therefore, it is crucial to investigate international differences in this pathway for CRC.

FIGURE 1

Module 4 of the International Cancer Benchmarking Partnership (ICBP M4) focusses on the routes to diagnosis and length of diagnostic and treatment intervals as a means of understanding differences in cancer prognosis between countries.[6] This will help shape policy and practice interventions in participating jurisdictions.

Diagnosis of CRC can be difficult; the symptoms are often vague (e.g. fatigue and non-specific abdominal pain), and this poses a significant diagnostic challenge for primary care, where most patients with CRC present.[7-12] There is growing evidence that prolonged diagnostic and treatment intervals are associated with poorer outcomes in CRC.[13-14] Access to investigations such as flexible sigmoidoscopy and colonoscopy is a further key issue; open access may expedite diagnosis and effect short diagnostic intervals.[12,15]

Many countries have implemented screening – typically faecal occult blood test (FOBT)-based – which can make a significant contribution to improved CRC outcomes.[16] However, currently, the large majority of CRC diagnoses are based on symptomatic presentation– for example, seeking help in primary care or attending emergency services.[7,17]

This study aims to systematically compare the diagnostic routes and time intervals from first noticing symptoms to start of treatment in CRC patients in ten healthcare systems with broadly similar access to high quality treatment and valid cancer registration.[6]

For peer review only - http://bmjopen.bmj.com/site/about/guidelines.xhtml

For peer review only

METHODS

The methods for ICBP M4 have been described.[18] In brief, we recruited patients through cancer registries in ten jurisdictions (Victoria, Manitoba, Ontario, Denmark, Northern Ireland, Norway, Sweden, England, Scotland and Wales). The target was to recruit 200 symptomatic recently diagnosed CRC patients per jurisdiction and to the patient, primary care, diagnostic, treatment and total intervals (Figure 2).

FIGURE 2

In defining these intervals we used principles articulated in the Aarhus Statement.[19] Data were collected from patients, their primary care physician (PCP) and their cancer treatment specialists (CTSs) as well as cancer registries. When calculating the route and time intervals we used predefined rules including a data 'hierarchy' around these information sources (Supplementary File 1). Based on a standardised protocol, teams within each jurisdiction established data collection processes with registries; survey logistics and data management were adapted to each local setting.

Data were transferred in anonymised format to the analysis team at Aarhus University – all data sources were combined into a single database.

Identification of study population

Eligible patients were consecutive patients aged 40 years or more with a first-diagnosis of CRC, ICD10 coded as C18.0-C18.9, C20.0 and C20.9.[20] Patients who had had another non-index cancer earlier were eligible, but those with synchronous different primary cancers were excluded[18].

Each jurisdiction used a registry-based identification to enhance validity. We aimed to recruit patients 3 – 6 months after diagnosis; this avoided approaching patients too soon after diagnosis, while mimimising recall bias from a long period post-diagnosis.

Recruitment was via cancer registries; either through 1) sending a letter to the relevant healthcare professional, requesting a pre-addressed envelope be forwarded to the patient on confirmation the person was aware of the diagnosis, or 2) registries via the research team or directly sent a letter to the patient.[18] Consent was required from all patients prior to participation and data transfer.

Data sources

Data from three questionnaires of eligible patients, their PCP and CTSs (Supplementary Files 2-4) were combined with information from participating cancer registries.

For peer review only - http://bmjopen.bmj.com/site/about/guidelines.xhtml

1. Survey data

Questionnaires were developed collaboratively with all jurisdictions. For consented patients, based on practice lists or the patient's response, a questionnaire was sent to the PCP with whom they were listed or who had been primarily involved in the diagnostic pathway. The patients and PCPs were asked about milestones, symptoms and route to cancer diagnosis. A questionnaire was sent to the CTSs who were first involved in the treatment. Jurisdictional differences in local recruitment processes are detailed in Supplementary File 5.

2. Registry data

To enhance complete and valid data on date of diagnosis, stage and screening status, data were collected through cancer registries wherever possible. Date of diagnosis was defined based on an established International Agency for Research on Cancer (IARC) hierarchy and stage was preferably given in tumour, node and metastasis (TNM) and Duke's.[21,22]

Data handling

Local teams entered data and questionnaire responses. They were validated for obvious errors where possible and queries discussed with local contacts. All survey data underwent cleaning centrally (Aarhus University) to ensure that the same explicit rules were applied on the full dataset. Patients where age, date of diagnosis or date of consent were unknown were excluded.

As described the data rules allowed the combination of data from different sources in a standardised way that ensured reproducibility and transparency (Supplementary File 1). The rules, based on the Aarhus Statement,[19] employed a 'hierarchy' principle in terms of the order in which data sources (patient, PCP, CTS, registry) should take precedence where responses between sources differed, and included imputation rules based on the available data. The exact rule was guided by the measure in question – for example, patient interval was collected primarily from the patient questionnaire whereas primary care time-points were collected from the PCP questionnaire. All the measures were further validated using algorithms for outliers and out of range responses (e.g. negative time intervals).

Although the protocol mandated contacting patients within a 3-6-month time window after diagnosis, some local registries needed to extend this period, primarily due to delays in recording the cancer diagnosis.

Measures of routes to diagnosis

We defined routes to diagnosis for CRC using categories derived from the Aarhus Statement check-list – the following categories were used in the anaylsis:[19]

For peer review only - http://bmjopen.bmj.com/site/about/guidelines.xhtml

- Screening
- Symptomatic:
  - Visit PCP
  - Visit PCP and Accident and Emergency (A&E)
  - A&E
  - Investigation for another problem
- Other/unknown routes to diagnosis

Measures of time intervals

To ensure international comparison the time interval definition was adapted from the Aarhus Statement and included the following time-points:[19]

- First onset of symptoms: the time-point when first bodily change(s) and/or symptom(s) are noticed by the patient.
- First presentation to healthcare: the time-point at which it would be at least possible for the clinician seeing the patient to have started investigating.
- First referral to secondary care: the time-point at which the PCP refers the patient (and responsibility of the patient) to secondary/specialist care.
- Date of diagnosis: date the definite diagnosis was made, defined by the IARC hierarchy.[21]
- Date of start of treatment: the date where the patient started curative or palliative treatment or a decision not to treat.

The time intervals were calculated as the number of days between these time-points (Figure 2). For screen detected CRC, the patient and primary care interval were not applicable, with other intervals calculated using screening date as the first time-point. All time-points were validated manually and negative intervals were set to 0 days. Missing day was imputed based on specific rules to ensure that the direction of a possible misclassification bias was known (Supplementary File 1).

Establishing screening status

CRC patients were categorised using data rules as 'screen-detected', 'symptomatic' or 'other presentation'. In some jurisdictions it was possible to identify screen-detected cancers from registries; in others this categorisation depended on questionnaire responses. Due to differences in the understanding and registration of screening across jurisdictions, we specified symptom-based detection should include all patients who reported symptoms or A&E/primary care presentation, even if the patient had indicated 'screening' as the diagnostic route (unless their PCP or CTS specified a screening route). For UK countries

For peer review only - http://bmjopen.bmj.com/site/about/guidelines.xhtml

the distinction between a screen-detected and non-screen-detected CRC was validated using registry data on screen-detected through public programs.

Covariates

Health status was measured using the self-reported general health item from the 36-Item Short Form Health Survey (SF36).[23] Comorbidity was assessed from the patient survey as presence of four diseases (stroke, diabetes, lung or heart diseases) and categorised into: 'none', 'medium' (one or two) or 'high' (three or four). Educational level was categorised as 'low' (vocational school or lower) and 'high' (university or higher). Symptoms reported were divided into two categories: 'a CRC specific symptom' or 'other symptoms'. This was based on a symptom coding done independently by two PCP-authors (DW and PV) with the aim of identifying symptoms where clinical suspicion could be raised.[24]

Statistical analysis

Quantile regression was used to estimate differences in intervals between all jurisdictions.[25] We compared the 50th, 75th and 90th percentiles. Wales was used as the reference jurisdiction as it had the lowest CRC survival according to the ICBP Module 1 cancer survival benchmark.[4] Quantile regression allows a comparison on the interval scale with optimal information on differences. Counting days, we used the 'qcount' procedure proposed by Miranda (2006).[26] Parameters were calculated with 1000 jittered samples. The differences in intervals between jurisdictions were calculated as marginal effects after quantile regression by setting the continuous covariate age to its mean value and the categorical covariates (gender and comorbidity) to their modes. Significance level was set to 0.05 or less, and 95% confidence intervals (95%CI) were calculated when appropriate. Statistical analyses were carried out using STATA v14 software.

Sensitivity and validity analyses

All analyses were undertaken using the 6 and 9 months cut-off criteria for allowable interval from diagnosis to questionnaire completion. To estimate the effect of using patient reported intervals only, a sensitivity analysis based solely on patient data was performed. The effect of excluding patients for whom at least one time interval hadn't been reported was also investigated.

Kappa coefficient and overall agreement percentage assessed the agreement on routes to diagnosis (screening and symptomatic presentation) between the different data sources. Kappa coefficients were interpreted using Landis' and Koch's criteria:[27] 0.00 – 0.20 = slight, 0.21 – 0.40 = fair, 0.41 – 0.60 =moderate, 0.61 – 0.80 = substantial, above 0.80 = almost perfect.

For peer review only - http://bmjopen.bmj.com/site/about/guidelines.xhtml

Agreement between the different data sources was also assessed by Lin's concordance correlation coefficient (CCC).[28] The ICBP M4 definition of screening-status was validated against registry data on screening where available, and assessed by Kappa and overall agreement percentage.

Patient involvement

The research questions for this survey drew on an extensive literature elating diagnosis and treatment delays leading to negative patient experiences. While patient experience was not a primary outcome measure for this study, patients were given the opportunity to comment on their experience through questionnaire free-text response options (under separate analysis). Patients were involved in the piloting of study instruments to ascertain if recruitment and questionnaire content and dissemination strategies were appropriate. Each jurisdiction has committed to comunicating the findings and local implications of this study to organisations representing their study participants.

RESULTS

Patient characteristics and participation

Of 14,664 eligible patients, 3,881 returned completed questionnaires (a 31% response rate, ranging from 19% in Norway to 69% in Denmark). Of these, 2,866 (95%) were included in the analyses after application of inclusion and exclusion criteria. The study flow with identification, exclusion and responses for each jurisdiction is seen in Table 1.

For peer review only

Table 1: Patient flow from identification to analyses for all ten jurisdictions and totally.

Jurisdiction	Wales	England	Scotland	N Ireland	Denmark	Manitoba	Norway	Sweden	Ontario	Victoria	Total
Start date	04-10-2013	01-11-2013	01-12-2013	06-08-2013	28-10-2013	01-05-2013	01-09-2014	01-02-2014	30-04-2014	01-07-2013	
	n (%)	n (%)	n (%)	n (%)	n (%)	n (%)	n (%)	n (%)	n (%)	n (%)	n (%)
**Eligible patients** [a, b]	**1.274 (100%)**	**1.314 (100%)**	**1.852 (92.4%)**	**568 (45.0%)**	**490 (79.9%)**	**1.288 (84.6%)**	**1.860 (95.5%)**	**537 (85.8%)**	**5.585 (71.8%)** [i]	**1.170 (58.7%)**	**14.664 (76.9%)**
**Packs sent to GP** [c]	**1.274 (100%)**	**1.198 (91.2%)**	**1.070 (57.8%)**								**3.542 (79.8%)**
- Pack not forwarded by GP	*211 (16.6%)*	*87 (7.3%)*	*103 (9.6%)*								401 (11.3%)
- Unsure if pack forwarded by GP	*333 (26.1%)*	*362 (30.2%)*	*209 (19.5%)*								904 (25.5%)
- Pack forwarded by GP	*730 (57.3%)*	*749 (62.5%)*	*758 (70.8%)*								2.237 (63.2%)
**Patients contacted by GP** [c, d]	**1.063 (83.4%)**	**1.111 (92.7%)**	**967 (90.4%)**								**3.141 (88.7%)**
**Patients approached directly** [c]				**555 (97.7%)**	**490 (100%)**	**761 (59.1%)**	**1.860 (100%)**	**537 (100%)**	**5.099 (91.3%)**	**1.049 (89.7%)**	**10.351 (70.6%)**
- Patient died						*49* (3.2%)			139 (1.8%)		188 (1.8%)
- Other				*13* (1.0%)		*26* (1.7%)			368 (4.7%)		407 (3.9%)
- No address						*11* (0.7%)			309 (4.0%)		320 (3.1%)
**Patient responses (% of eligible patients)** [c]	**314 (24.6%)**	**285 (21.7%)**	**337 (18.2%)**	**283 (49.8%)**	**340 (69.4%)**	**274 (21.3%)**	**358 (19.2%)**	**319 (59.4%)**	**899 (16.1%)**	**472 (40.3%)**	**3.881 (26.5%)**
**Patient responses (% of contacted)** [e]	**314 (29.5%)**	**285 (25.7%)**	**337 (34.9%)**	**283 (52.2%)**	**340 (69.4%)**	**274 (40.6%)**	**358 (19.2%)**	**319 (59.4%)**	**899 (21.0%)**	**472 (45.0%)**	**3.881 (30.9%)**
- Did not fulfil eligibility criteria	7 (2.2%)	1 (0.4%)		1 (0.4%)		1 (0.4%)					*10 (0.3%)*
- Received after submission				20 (7.1%)						55 (11.7%)	*75 (2.1%)*
- Other	7 (2.2%)		57 (16.9%)	6 (2.1%)	16 (4.7%)	11 (4.0%)			45 (5.0%)	127 (26.9%)	*269 (7.6%)*
**Patient surveys forwarded for analyses** [f]	**300 (95.5%)**	**284 (99.6%)**	**280 (83.1%)**	**256 (90.5%)**	**324 (95.3%)**	**262 (95.6%)**	**358 (100%)**	**319 (100%)**	**854 (95.0%)**	**290 (61.4%)**	**3.527 (90.9%)**
Excluded for analyses – total	*17 (5.7%)*	*10 (3.5%)*	*7 (2.5%)*	*3 (1.2%)*	*7 (2.2%)*	*4 (1.5%)*	*72 (20.1%)*	*8 (2.5%)*	*532 (62.3%)*	*1 (0.3%)*	*661 (18.7%)*
- Not sampled	*0 (0%)*	*0 (0%)*	*0 (0%)*	*0 (0%)*	*0 (0%)*	*0 (0%)*	*0 (0%)*	*0 (0%)*	*494 (57.8%)*	*0 (0%)*	*494 (14%)*
- Previous cancer	*0 (0%)*	*3 (1.1%)*	*0 (0%)*	*0 (0%)*	*0 (0%)*	*0 (0%)*	*0 (0%)*	*0 (0%)*	*1 (0.1%)*	*0 (0%)*	*4 (0.1%)*
- Unknown date of consent	*0 (0%)*	*0 (0%)*	*0 (0%)*	*0 (0%)*	*0 (0%)*	*0 (0%)*	*3 (0.8%)*	*6 (1.9%)*	*0 (0%)*	*0 (0%)*	*9 (0.3%)*
- Unknown date of diagnosis	*0 (0%)*	*0 (0%)*	*0 (0%)*	*0 (0%)*	*0 (0%)*	*1 (0.4%)*	*6 (1.7%)*	*0 (0%)*	*0 (0%)*	*0 (0%)*	*7 (0.2%)*
- Consent too late/too early	*17 (5.7%)*	*7 (2.5%)*	*7 (2.5%)*	*3 (1.2%)*	*0 (0%)*	*3 (1.1%)*	*60 (16.8%)*	*1 (0.3%)*	*37 (4.3%)*	*1 (0.3%)*	*136 (3.9%)*
- Other	*0 (0%)*	*0 (0%)*	*0 (0%)*	*0 (0%)*	*7 (2.2%)*	*0 (0%)*	*3 (0.8%)*	*1 (0.3%)*	*0 (0%)*	*0 (0%)*	*11 (0.3%)*
**Patient surveys anlysed (% of forwarded surveys)**	**283 (94.3%)**	**274 (96.5%)**	**273 (97.5%)**	**253 (98.8%)**	**317 (97.8%)**	**258 (98.5%)**	**286 (79.9%)**	**311 (97.5%)**	**322 (89.4%)**	**289 (99.7%)**	**2.866 (94.5%)** [k]
**GP surveys analysed (% of analysed patients)**	**234 (82.7%)**	**225 (82.1%)**	**224 (82.1%)**	**213 (84.2%)**	**241 (76.0%)**	**148 (57.4%)**	**169 (59.1%)**	**n/a n/a**	**121 (37.7%)**	**199 (68.9%)**	**1.774 (69.5%)** [l]
**Specialist surveys analysed (% of analysed patients)**	**164 (58.0%)**	**156 (56.9%)**	**179 (65.6%)**	**n/a** [g] **n/a**	**187** [g] **(59%)**	**n/a** [h] **n/a**	**64 (22.4%)**	**n/a** [h] **n/a**	**89 (27.7%)**	**99 (34.3%)**	**938 (45.9%)** [m]

[a] Eligible according to protocol: i.e. woman, 40 years or more, alive, consented to participate within nine months of diagnosis, diagnosed with breast cancer (ICD-10: C50.0-C50.9), behaviour code ICD-O-3=3 and without prior history of cancer of the breast or synchronous primary breast cancer. [b] In some jurisdictions some 'eligible' patients had pre-opted out from being contacted and a small number where GP information was not available. [c] Percentages of eligible patients. [d] Maximum of potentially contacted patients, i.e. sum of packs forwarded by GP and packs unsure if forwarded by GP. [e] Percentages of patients contacted by GP (see note d) for Wales, England and Scotland or percentages of patients contacted directly by a registry excl. non-accessible patients (all other jurisdictions). [f] Percentages of patient responses. [g] Data obtained from registries instead: N Ireland from from the N.Ireland Cancer Registry supported by extracts from clinical datasets; Denmark from the Danish Colorectal Cancer Group (DCCG). [h] Data not collected in this jurisdiction. [i] initially additionally 1,458 cases were eligible but excluded for this study as urban sample size was reached. [j] Additionally 92 cases were ineligible based on registry-criteria exclusions and a further 108 excluded after treating doctor approach. [k] Denominator = total number of forwarded cases excl. patients not included in analytic sample in Ontario. [l] Denominator = total number of analysed cases excl. patients from Sweden. [m] Denominator = total number of analysed cases excl. patients from Sweden, Manitoba & N Ireland.

For peer review only

For peer review only - http://bmjopen.bmj.com/site/about/guidelines.xhtml

The characteristics of the included patients are detailed in Table 2. For tumour stage the proportion of missing stage was high in Norway due to lack of registry data.

Table 2. *The characteristics of the 2866 patients aged 40 or over with a first diagnosis of CRC included in the analyses (% if nothing else stated)*

	**Wales (N=283)**	**England (N=274)**	**Scotland (N=273)**	**N Ireland (N=253)**	**Denmark (N=317)**	**Manitoba (N=258)**	**Norway (N=286)**	**Sweden (N=311)**	**Ontario (N=322)**	**Victoria (N=289)**	**Total (N=2866)**
**Age** years											
Median (IQI)	71 (65,79)	71 (64,78)	70 (61,77)	67 (60,74)	71 (65,77)	69 (59,77)	70 (62,77)	72 (65,79)	69 (61,77)	66 (58,76)	70 (62,77)
**Age** years (Symptomatic patients)	(N=208)	(N=212)	(N=192)	(N=214)	(N=311)	(N=176)	(N=264)	(N=307)	(N=257)	(N=220)	(N=2361)
Median (IQI)	72 (64,81)	72 (64,80)	72 (61,80)	67 (59,76)	71 (65,77)	73 (60,81)	70 (62,77)	72 (65,79)	69 (61,77)	69 (57,77)	71 (62,78)
**Age** years (Screen-detected patients)	(N=73)	(N=58)	(N=78)	(N=35)	(N=0)	(N=81)	(N=18)	(N=0)	(N=55)	(N=62)	(N=460)
Median (IQI)	67 (65,73)	68 (65,74)	66 (62,72)	68 (63,70)	n/a	65 (57,70)	69 (61,74)	n/a	68 (63,72)	65 (60,69)	67 (63,72)
**Gender**											
Male	59.0	56.2	58.6	58.9	59.0	56.6	51.4	51.1	56.5	56.1	56.3
**Health State**											
Good	80.6	84.7	84.6	75.5	82.3	88.0	74.1	78.8	85.1	82.4	81.6
Fair	13.8	11.7	10.6	17.4	13.6	9.3	22.0	16.1	11.5	12.1	13.8
Poor	5.0	3.3	3.3	6.7	1.6	1.9	2.8	3.2	2.5	4.2	3.4
Missing	0.7	0.4	15	0.4	2.5	0.8	1.1	1.9	0.9	1.4	1.2
**Comorbidity** [1]											
No	52.7	58.8	57.1	55.3	50.2	63.2	61.9	58.5	53.7	61.9	57.2
Medium	44.2	38.7	41.8	43.5	46.7	34.9	35.0	35.1	31.4	35.6	38.6
High	2.5	2.6	0.7	1.2	1.9	1.6	3.2	4.5	1.9	1.7	2.2
Missing	0.7	0.0	0.4	0.0	1.3	0.4	0.0	1.9	13.0	0.7	2.0
**Education**											
Low	76.7	80.7	74.7	77.5	76.0	80.2	75.2	78.8	73.3	77.5	77.0
High	15.6	14.2	19.8	13.0	12.0	17.8	18.9	20.3	23.9	21.5	17.8
Missing	7.8	5.1	5.5	9.5	12.0	1.9	5.9	1.0	2.8	1.0	5.2
**Ethnicity**											
White	99.9	98.5	98.5	99.6	95.9	93.4	99.7	99.4	92.6	94.5	97.1
Asian	0.4	0.4	0.7	0.0	0.3	1.9	0.0	0.3	5.9	2.4	1.3
Black	0.0	0.0	0.4	0.0	0.0	0.4	0.0	0.0	0.6	0.0	0.1
Other	0.0	0.0	0.0	0.0	0.0	3.5	0.0	0.0	0.3	0.0	0.4
Missing	0.0	1.1	0.4	0.4	3.8	0.8	0.3	0.3	0.6	3.1	1.1
**Smoking**											
Currently	4.2	2.6	8.1	9.1	11.4	8.9	7.0	4.8	4.4	4.8	6.5
In the past	55.5	54.7	51.3	49.0	55.2	50.8	56.3	52.7	59.3	51.6	53.8
Never	39.9	41.2	40.7	39.5	31.6	39.9	36.4	42.4	35.4	42.6	38.8
Missing	0.4	1.5	0.0	2.4	1.9	0.4	0.3	0.0	0.9	1.0	0.9
**Tumor stage – TNM & Dukes**											
0	0.4	0.4	0.0	0.0	0.0	0.0	0.0	0.0	0.6	0.0	0.1
I	19.4	20.8	18.7	18.2	20.5	17.1	3.9	16.7	27.0	26.0	19.0
II	25.4	29.6	32.6	29.6	36.0	28.3	4.9	26.1	32.0	35.6	28.1

For peer review only - http://bmjopen.bmj.com/site/about/guidelines.xhtml

	Wales (N=283)	England (N=274)	Scotland (N=273)	N Ireland (N=253)	Denmark (N=317)	Manitoba (N=258)	Norway (N=286)	Sweden (N=311)	Ontario (N=322)	Victoria (N=289)	Total (N=2866)
III	40.3	30.7	35.9	37.6	24.6	39.5	7.0	31.5	28.0	29.1	30.1
IV	9.5	16.1	12.1	13.4	13.9	11.6	4.6	17.4	6.5	8.3	11.3
Missing	5.0	2.6	0.7	1.2	5.0	3.5	79.7	8.4	5.9	1.0	11.4
**Tumor stage – TNM & Dukes** (Symptomatic patients)	(N=208)	(N=212)	(N=192)	(N=214)	(N=311)	(N=176)	(N=264)	(N=307)	(N=256)	(N=220)	(N=2360)
0	0.5	0.5	0.0	0.0	0.0	0.0	0.0	0.0	0.4	0.0	0.1
I	16.4	17.0	13.5	14.0	20.9	15.3	3.8	16.6	22.6	20.9	16.2
II	26.4	30.7	34.9	31.8	35.1	29.6	4.2	26.4	33.5	36.8	28.6
III	39.9	31.6	36.5	38.3	25.1	36.9	7.2	31.6	29.6	30.9	29.9
IV	11.5	17.0	14.1	14.5	13.8	13.1	4.9	17.3	8.2	10.0	12.4
Missing	5.3	3.3	1.0	1.4	5.1	5.1	79.9	8.1	5.8	1.4	12.8
**Tumor stage – TNM & Dukes** (Screen-detected patients)	(N=73)	(N=58)	(N=78)	(N=35)	(N=0)	(N=81)	(N=18)	(N=0)	(N=55)	(N=62)	(N=460)
0	0.0	0.0	0.0	0.0	n/a	0.0	0.0	n/a	1.8	0.0	0.2
I	28.8	36.2	30.8	40.0	n/a	21.0	5.6	n/a	47.3	47	33.3
II	23.3	24.1	28.2	17.1	n/a	24.7	16.7	n/a	21.8	29.0	24.4
III	41.1	27.6	33.3	34.3	n/a	45.7	5.6	n/a	21.8	21.0	32.0
IV	2.7	12.1	7.7	8.6	n/a	8.6	0.0	n/a	0.0	3.2	5.9
Missing	4.1	0.0	0.0	0.0	n/a	0.0	72.2	n/a	7.3	0.0	4.4

[1] Comorbidity coded as none=no reported, medium=1-2 reported and high=3+ reported
Abbreviations: IQI=inter-quartile interval; n/a=not applicable

Routes to diagnosis

Routes to diagnosis were broadly similar, with the exception of screening; of all patients, 16.1% had a screen detected CRC, ranging from 6.3% in Norway to 31.4% in Manitoba (Table 3). In Denmark and Sweden CRC-screening had not been implemented at the time of study and screening status in Norway was determined by information from local screening trials. The proportion of screen-detected cancers in Northern Ireland is lower as most were excluded in the recruitment process. Overall (excluding Northern Ireland), most (82.2%) respondents presented with a symptomatic CRC. A high level of agreement was found between ICBP and registry data for screening status (Table 3). PCP data indicated 53.1% of the symptomatic patients were urgently referred with a suspicion of cancer; ranging from 36.6% in Ontario to 69.3% in Wales (data not shown).

For peer review only - http://bmjopen.bmj.com/site/about/guidelines.xhtml

Table 3. *The overall route (symptomatic or screened) for CRC in each jurisdiction (%) and place of initial presentation for symptomatic patients*

	Wales (N=283)	England (N=274)	Scotland (N=273)	N Ireland[1] (N=253)	Denmark (N=317)	Manitoba (N=258)	Norway[2] (N=286)	Sweden (N=311)	Ontario (N=322)	Victoria (N=289)	Total (N=2866)
**Symptomatic**	**73.5**	**77.3**	**70.3**	**84.6**	**98.1**	**68.2**	**92.3**	**99.0**	**79.8**	**76.1**	**82.4**
Visit PCP, Visit PCP and A&E[3]	*77.4*	*82.6*	*92.3*	*83.2*	*82.3*	*67.1*	*78.4*	*51.8*	*62.3*	*73.2*	*73.2*
A&E[3]	*7.2*	*3.8*	*0.5*	*6.5*	*3.2*	*9.1*	*4.6*	*11.7*	*8.2*	*8.6*	*6.7*
Investigation for another problem[3]	*6.7*	*4.7*	*4.8*	*3.3*	*9.3*	*9.7*	*9.9*	*32.9*	*11.3*	*7.3*	*11.1*
Other[3]	*8.7*	*9.0*	*2.4*	*7.0*	*5.1*	*14.2*	*7.2*	*3.6*	*18.3*	*10.9*	*8.9*
**Screening**	**25.8**	**21.2**	**28.6**	**13.8**	**0.0**	**31.4**	**6.3**	**0.0**	**17.1**	**21.5**	**16.1**
**Other**	**0.7**	**1.5**	**1.1**	**1.6**	**1.9**	**1.0**	**1.4**	**1.0**	**3.1**	**2.4**	**1.5**

Agreement between the ICBP M4 Presentation-rule (without using registry data) and registry information to define if a CRC case was screen detected:

Jurisdiction	Number of cases	Agreement on screening-status between ICBP4 and Registry %	Kappa	(95%CI)
Wales	277	92	0.78	(0.68-0.86)
England	259	95	0.84	(0.74-0.92)
Scotland	270	93	0.83	(0.75-0.90)
N Ireland	251	94	0.75	(0.61-0.87)

[1] In N Ireland the proportion of screen detected CRC cases was lower as these patients were primarily excluded from the eligible group
[2] The reporting of screening status in Norway was based on local screening trials.
[3] Percentage of Symptomatic-route

For peer review only

Symptom prompting concern

The proportion (%) of patients and PCPs reporting symptoms are shown in Table 4 (for Northern Ireland and Sweden, only patient data were collected). The most common symptom reported by PCP respondents was rectal bleeding (40% of respondents), followed by change in bowel habit. While every third patient indicated fatigue as a key symptom, it was rarely reported by PCPs.

For peer review only - http://bmjopen.bmj.com/site/about/guidelines.xhtml

Table 4. *The symptoms experienced by patients before presentation and the presenting symptosm seen by the PCP for the 2,361 patients aged 40 or over with a first diagnosis of CRC who had a symptom based diagnosis. All figures are %*

	Wales	England	Scotland	N Ireland	Denmark	Manitoba	Norway	Sweden	Ontario	Victoria	Total
**First symptom (reported by patient)**	(N=208)	(N=212)	(N=192)	(N=214)	(N=311)	(N=176)	(N=264)	(N=307)	(N=257)	(N=220)	(N=2361)
Bleeding/blood in stool	43.8	41.0	37.5	43.0	34.7	42.0	51.9	47.2	50.6	37.3	43.1
Bowel habit change	42.3	33.5	33.9	42.1	31.8	27.3	28.4	35.8	48.3	31.4	35.5
Fatigue	31.3	33.5	34.4	37.9	19.3	31.3	24.6	30.0	31.5	29.6	29.7
Abdominal pain	24.0	25.0	28.7	27.6	19.3	26.7	22.0	19.2	22.2	28.2	23.7
Weight loss	18.8	19.3	22.4	17.3	14.2	14.7	14.0	18.2	15.6	16.4	16.9
Other	40.4	35.9	37.0	42.5	27.3	34.7	31.1	40.1	18.7	30.5	33.4
No symptoms	6.7	3.8	9.9	4.7	9.7	14.8	7.6	9.5	5.8	8.2	8.0
Missing	1.4	1.9	2.6	1.9	10.3	3.4	3.0	1.0	1.2	0.9	3.0
**Number of symptoms per patient**											
Median (IQI)	2(1,3)	2(1,3)	2(1,3)	2(1,3)	1(1,2)	2(1,3)	2(1,2)	2(1,3)	2(1,3)	2(1,3)	2(1,3)
**Presenting symptom (reported by PCP)**	(N=140)	(N=151)	(N=132)	(N=0)	(N=212)	(N=77)	(N=134)	(N=0)	(N=82)	(N=118)	(N=1046)
Bleeding/blood in stool	37.1	33.8	33.3	n/a	29.1	26.0	29.1	n/a	32.9	33.9	31.9
Bowel habit change	33.6	27.8	21.2	n/a	26.9	16.9	17.9	n/a	19.5	21.2	24.1
Fatigue	4.3	1.3	3.8	n/a	7.6	5.2	4.5	n/a	9.8	8.5	5
Abdominal pain	20.7	15.2	17.4	n/a	18.9	16.9	12.7	n/a	15.9	26.3	18.1
Weight loss	8.6	6.6	8.3	n/a	7.6	1.3	3.0	n/a	3.7	6.8	6.2
Other	36.4	30.5	36.4	n/a	34.9	27.3	33.6	n/a	18.3	39.8	33.2
No symptoms	3.6	4.6	6.8	n/a	0.5	15.6	0.0	n/a	3.7	1.7	3.7
Missing	8.6	8.6	9.1	n/a	16.5	26.0	8.2	n/a	17.1	11.0	12.4
**Cancer-specificity of symptom presented**											
Cancer-specific symptom	67.9	66.2	56.1	n/a	57.6	46.8	53.0	n/a	48.8	63.6	58.6
Non-specific symptom	20.0	20.5	28.0	n/a	25.5	11.7	38.8	n/a	30.5	23.7	25.2
No symptoms /missing	12.1	13.3	15.9	n/a	17.0	41.6	8.2	n/a	20.7	12.7	16.2

Abbreviations: IQI=inter-quartile interval; n/a=not applicable

For peer review only

Time intervals

The median patient interval varied from 21 days (Denmark) to 49 days (Wales) (Table 5). Differences in days between intervals (using Wales as a reference) were calculated as marginal effects after quantile regression by setting the continuous covariate age to its mean value and categorical covariates (gender and comorbidity) to their modes. Table 6 shows the adjusted patient median interval was 25 days shorter in Denmark than in Wales; none were longer compared to Wales (Supplementary File 6).

For peer review only - http://bmjopen.bmj.com/site/about/guidelines.xhtml

Table 5. *The time intervals (days) for each of the ten jurisdictions depicted as median, 75th and 90th percentiles. In Sweden no data on the primary care interval was available*

		Wales	England	Scotland	N Ireland	Denmark	Manitoba	Norway	Sweden	Ontario	Victoria
Patient Interval (Symptomatic patients)	Number	(n=195)	(n=199)	(n=175)	(n=199)	(n=292)	(n=134)	(n=240)	(n=266)	(n=230)	(n=199)
	Median	49	34	30	35	21	34	36	31	31	22
	75th centile	92	118	73	88	62	92	92	92	96	63
	90th centile	249	346	181	312	180	215	218	201	304	234
Primary Care interval (Symptomatic patients)	Number	(n=157)	(n=152)	(n=127)	(n=160)	(n=207)	(n=72)	(n=124)	(n=0)	(n=77)	(n=117)
	Median	3	2	4	0	1	4	12	n/a	1	9
	75th centile	20	21	28	14	10	30	39	n/a	23	32
	90th centile	78	54	93	54	51	210	82	n/a	70	128
Diagnostic interval (Symptomatic patients)	Number	(n=194)	(n=196)	(n=174)	(n=190)	(n=290)	(n=133)	(n=229)	(n=249)	(n=218)	(n=197)
	Median	60	48	38	64	27	76	37	36	54	28
	75th centile	155	86	91	111	66	162	85	82	146	66
	90th centile	284	201	164	238	129	365	222	196	312	200
Diagnostic interval (Screen-detected patients)	Number	(n=69)	(n=56)	(n=76)	(n=35)	(n=0)	(n=25)	(n=14)	(n=0)	(n=50)	(n=38)
	Median	35	25	36	0	n/a	66	22	n/a	3	40
	75th centile	65	46	49	0	n/a	111	48	n/a	43	64
	90th centile	99	70	76	0	n/a	206	84	n/a	120	122
Diagnostic interval (All patients)	Number	(n=263)	(n=252)	(n=250)	(n=225)	(n=290)	(n=158)	(n=243)	(n=249)	(n=268)	(n=235)
	Median	52	43	37	47	27	72	36	36	44	28
	75th centile	120	76	72	101	66	139	85	82	128	64
	90th centile	242	176	151	207	129	320	212	196	278	178
Treatment interval (Symptomatic patients)	Number	(n=197)	(n=206)	(n=185)	(n=208)	(n=306)	(n=161)	(n=258)	(n=281)	(n=248)	(n=209)
	Median	39	31	33	25	14	34	18	35	33	14
	75th centile	59	47	56	40	19	59	29	52	54	29
	90th centile	83	60	79	58	28	97	45	65	79	47
Treatment interval (Screen-detected patients)	Number	(n=72)	(n=58)	(n=78)	(n=34)	(n=0)	(n=79)	(n=17)	(n=0)	(n=52)	(n=60)
	Median	44	39	49	38	n/a	38	19	n/a	40	17
	75th centile	68	46	71	52	n/a	61	27	n/a	54	35
	90th centile	80	62	91	61	n/a	83	43	n/a	88	44
Treatment interval (All patients)	Number	(n=271)	(n=268)	(n=266)	(n=246)	(n=312)	(n=240)	(n=279)	(n=284)	(n=310)	(n=276)
	Median	41	34	37	27	14	35	18	36	34	15
	75th centile	63	47	63	42	19	60	28	53	54	29
	90th centile	80	61	87	59	27	88	43	65	82	44
Total interval (Symptomatic patients)	Number	(n=154)	(n=165)	(n=147)	(n=175)	(n=249)	(n=123)	(n=210)	(n=238)	(n=214)	(n=168)
	Median	168	145	120	138	77	154	108	127	124	90
	75th centile	304	248	184	235	146	307	203	224	251	182
	90th centile	365	365	326	365	248	365	312	365	365	357
Total interval (All patients)	Number	(n=222)	(n=221)	(n=223)	(n=209)	(n=249)	(n=148)	(n=224)	(n=238)	(n=262)	(n=205)
	Median	128	112	103	111	77	136	102	127	105	74
	75th centile	239	201	159	211	146	266	194	224	230	153
	90th centile	365	365	253	365	248	365	307	365	365	320

For peer review only - http://bmjopen.bmj.com/site/about/guidelines.xhtml

Table 6. *Analyses of the differences in intervals (days) between Wales as the reference and the other nine jurisdictions.*

		Wales	England	Scotland	N Ireland	Denmark	Manitoba	Norway	Sweden	Ontario	Victoria
Patient Interval (Symptomatic patients)	Number	(n=195)	(n=199)	(n=175)	(n=199)	(n=292)	(n=134)	(n=240)	(n=266)	(n=230)	(n=199)
	Median	49 (ref.)	-6 (-25,13)	-14 (-29,2)	-11 (-42,20)	**-25** (-38,-11)	-11 (-30,9)	-9 (-46,27)	-7 (-21,7)	-13 (-30,4)	**-23** (-32,-15)
	75th centile	92 (ref.)	14 (-9,38)	**-28** (-44,-12)	-13 (-32,6)	**-28** (-47,-9)	1 (-28,30)	-3 (-22,16)	0 (-29,29)	11 (-8,29)	**-30** (-51,-9)
	90th centile	249 (ref.)	23 (-14,60)	**-74** (-124,-24)	**26** (10,42)	-60 (-174,55)	-17 (-65,31)	-43 (-85,0)	**-43** (-60,-27)	3 (-109,115)	-33 (-87,21)
Primary Care interval (Symptomatic patients)	Number	(n=157)	(n=152)	(n=127)	(n=160)	(n=207)	(n=72)	(n=124)	(n=0)	(n=77)	(n=117)
	Median	3 (ref.)	-2 (-5,2)	1 (-4,6)	-3 (-5,0)	-2 (-5,1)	0 (-8,8)	**7** (3,12)	n/a	-2 (-5,1)	6 (0,13)
	75th centile	20 (ref.)	-1 (-14,12)	9 (-6,25)	-7 (-20,6)	-10 (-25,4)	9 (-3,21)	**21** (3,39)	n/a	3 (-8,15)	13 (-6,31)
	90th centile	78 (ref.)	-14 (-31,3)	**42** (11,73)	-23 (-64,17)	-19 (-91,53)	**124** (63,186)	36 (0,71)	n/a	3 (-43,49)	36 (-16,88)
Diagnostic interval (Symptomatic patients)	Number	(n=194)	(n=196)	(n=174)	(n=190)	(n=290)	(n=133)	(n=229)	(n=249)	(n=218)	(n=197)
	Median	60 (ref.)	**-11** (-18,-4)	**-20** (-27,-13)	5 (-6,16)	**-29** (-35,-24)	14 (-1,29)	**-20** (-28,-12)	**-19** (-35,-3)	-1 (-11,8)	**-28** (-35,-21)
	75th centile	155 (ref.)	**-56** (-119,7)	**-59** (-113,-5)	**-44** (-81,-7)	**-83** (-110,-55)	-6 (-60,47)	**-67** (-93,-42)	**-65** (-117,-13)	-3 (-52,46)	**-81** (-131,-32)
	90th centile	284 (ref.)	**-78** (-116,-40)	**-114** (-132,-96)	**-54** (-89,-19)	**-130** (-161,-99)	**33** (9,56)	**-80** (-114,-46)	**-86** (-116,-56)	15 (-2,32)	**-78** (-103,-53)
Diagnostic interval (Screen-detected patients)	Number	(n=69)	(n=56)	(n=76)	(n=35)	(n=0)	(n=25)	(n=14)	(n=0)	(n=50)	(n=38)
	Median	35 (ref.)	**-8** (-13,-4)	3 (-2,8)	**-32** (-36,-28)	n/a	**31** (19,43)	**-15** (-24,-5)	n/a	**-25** (-34,-15)	1 (-8,10)
	75th centile	65 (ref.)	**-18** (-28,-8)	-12 (-29,4)	**-63** (-70,-55)	n/a	**36** (6,66)	**-24** (-42,-6)	n/a	**-17** (-26,-8)	6 (-20,33)
	90th centile	99 (ref.)	**-17** (-28,-6)	12 (-16,41)	**-98** (-101,-96)	n/a	**90** (32,147)	**-35** (-43,-27)	n/a	**40** (30,51)	**32** (25,40)
Diagnostic interval (All patients)	Number	(n=263)	(n=252)	(n=250)	(n=225)	(n=290)	(n=158)	(n=243)	(n=249)	(n=268)	(n=235)
	Median	52 (ref.)	**-9** (-15,-4)	**-13** (-19,-7)	-2 (-12,7)	**-22** (-28,-17)	**21** (13,29)	**-14** (-24,-3)	**-13** (-21,-4)	-4 (-11,2)	**-21** (-26,-15)
	75th centile	120 (ref.)	**-34** (-49,-18)	**-38** (-53,-23)	-10 (-31,10)	**-44** (-56,-32)	**22** (4,41)	**-29** (-50,-9)	**-27** (-38,-17)	**18** (2,34)	**-46** (-57,-34)
	90th centile	242 (ref.)	**-73** (-101,-45)	**-91** (-118,-64)	**-41** (-57,-24)	**-106** (-127,-85)	**50** (7,94)	**-53** (-88,-18)	**-59** (-88,-30)	**44** (27,61)	**-54** (-82,-26)
Treatment interval (Symptomatic patients)	Number	(n=197)	(n=206)	(n=185)	(n=208)	(n=306)	(n=161)	(n=258)	(n=281)	(n=248)	(n=209)
	Median	39 (ref.)	**-6** (-11,-1)	-5 (-11,0)	**-13** (-19,-8)	**-24** (-27,-21)	-5 (-14,5)	**-20** (-23,-16)	-3 (-8,2)	**-6** (-10,-1)	**-23** (-28,-19)
	75th centile	59 (ref.)	**-13** (-18,-8)	-4 (-11,4)	**-19** (-24,-13)	**-41** (-48,-34)	0 (-9,10)	**-30** (-37,-24)	**-8** (-16,-1)	-6 (-12,0)	**-31** (-38,-25)
	90th centile	83 (ref.)	**-27** (-38,-16)	-5 (-20,11)	**-29** (-42,-17)	**-61** (-72,-50)	4 (-13,20)	**-43** (-59,-27)	**-24** (-35,-14)	-8 (-21,4)	**-42** (-52,-31)
Treatment interval (Screen-detected patients)	Number	(n=72)	(n=58)	(n=78)	(n=34)	(n=0)	(n=79)	(n=17)	(n=0)	(n=52)	(n=60)
	Median	44 (ref.)	-4 (-14,5)	3 (-6,12)	-5 (-15,5)	n/a	-8 (-17,2)	**-28** (-41,-16)	n/a	**-8** (-14,-2)	**-26** (-36,-16)
	75th centile	68 (ref.)	**-22** (-26,-19)	0 (-5,5)	**-22** (-25,-18)	n/a	**-13** (-22,-3)	**-45** (-49,-42)	n/a	**-23** (-26,-20)	**-37** (-45,-29)
	90th centile	80 (ref.)	**-21** (-32,-9)	6 (-3,14)	**-23** (-31,-15)	n/a	-6 (-15,3)	**-37** (-45,-30)	n/a	11 (-1,22)	**-42** (-51,-33)
Treatment interval (All patients)	Number	(n=271)	(n=268)	(n=266)	(n=246)	(n=312)	(n=240)	(n=279)	(n=284)	(n=310)	(n=276)
	Median	41 (ref.)	**-6** (-10,-2)	-2 (-9,5)	**-13** (-18,-9)	**-26** (-30,-21)	-4 (-10,1)	**-22** (-27,-16)	-5 (-10,0)	**-6** (-11,-1)	**-24** (-29,-20)
	75th centile	63 (ref.)	**-15** (-21,-8)	2 (-10,14)	**-19** (-27,-11)	**-42** (-50,-35)	0 (-7,7)	**-32** (-39,-24)	**-9** (-16,-1)	-8 (-17,2)	**-32** (-46,-17)
	90th centile	80 (ref.)	**-24** (-31,-17)	3 (-12,18)	**-25** (-34,-17)	**-59** (-66,-51)	4 (-4,12)	**-41** (-54,-29)	**-22** (-30,-15)	-4 (-19,11)	**-40** (-50,-30)
Total interval (Symptomatic patients)	Number	(n=154)	(n=165)	(n=147)	(n=175)	(n=249)	(n=123)	(n=210)	(n=238)	(n=214)	(n=168)
	Median	168 (ref.)	**-30** (-55,-6)	**-52** (-99,-5)	-36 (-93,21)	**-92** (-106,-78)	-25 (-86,35)	**-63** (-74,-52)	**-43** (-63,-24)	-44 (-98,9)	**-85** (-97,-73)
	75th centile	304 (ref.)	-35 (-78,8)	**-101** (-130,-71)	-34 (-91,22)	**-137** (-177,-97)	-10 (-24,4)	**-84** (-129,-38)	**-62** (-92,-32)	-44 (-91,2)	**-117** (-137,-97)
	90th centile	365 (ref.)	0 (-1,0)	**-39** (-47,-32)	0 (-1,0)	**-125** (-134,-116)	0 (-1,0)	**-49** (-58,-41)	0 (-1,0)	0 (-1,0)	-7 (-16,1)
Total interval (All patients)	Number	(n=222)	(n=221)	(n=223)	(n=209)	(n=249)	(n=148)	(n=224)	(n=238)	(n=262)	(n=205)
	Median	128 (ref.)	-13 (-35,9)	**-22** (-36,-8)	-14 (-38,10)	**-46** (-62,-30)	10 (-13,32)	**-19** (-34,-5)	-1 (-16,15)	**-18** (-34,-2)	**-44** (-70,-18)
	75th centile	239 (ref.)	**-32** (-63,-1)	**-81** (-126,-35)	-26 (-64,12)	**-89** (-137,-41)	10 (-33,52)	**-43** (-70,-17)	-18 (-59,23)	-16 (-80,49)	**-88** (-119,-57)
	90th centile	365 (ref.)	-1 (-2,0)	**-108** (-129,-88)	-1 (-3,1)	**-118** (-127,-109)	0 (-2,2)	**-56** (-80,-32)	0 (-1,0)	0 (-2,1)	**-46** (-54,-37)

For peer review only - http://bmjopen.bmj.com/site/about/guidelines.xhtml

The differences for the 50th (median), 75th and 90th percentiles are calculated as marginal effects after quantile regression by setting the continuous covariate age to its mean value and categorical covariates (gender and comorbidity) to their modes. The actual number of days are included for Wales in Table 5.

For peer review only

For peer review only - http://bmjopen.bmj.com/site/about/guidelines.xhtml

The median primary care interval was 12 days in Norway (Table 5), statistically significantly longer than Wales (Table 6). For the 10% of patients waiting longest for referral, the longest intervals were observed in Manitoba, Victoria and Scotland (210, 128 and 93 days, respectively) (Table 5). This interval at the 90th percentile was either 4 months (Manitoba) or 1 month (Victoria and Scotland) longer than in Wales (Table 6).

The median diagnostic interval for symptomatic patients ranged from 27-28 days in Denmark and Victoria to 76 days in Manitoba. At 90th percentile it ranged from 4 months in Denmark to 10 months in Ontario (Table 5). All jurisdictions had shorter median diagnostic intervals compared to Wales, except Northern Ireland and Manitoba, where the intervals were 5 and 14 days longer, respectively (Table 6).

The shortest median treatment intervals for all patients (about 2 weeks) were observed in Denmark, Victoria and Norway. In other jurisdictions this interval was 1 month or more (Table 5). All jurisdictions had shorter treatment intervals compared to Wales, except Scotland and Manitoba (Table 6).

The median total interval (from first symptom to treatment start) for all CRC patients was between 74 days (Victoria) and 136 days (Manitoba) (Table 5). In Scotland, Denmark, Norway and Victoria, this interval was statistically significantly shorter than in Wales (Table 6).

Sensitivity and validity analyses

Changing the cut-off survey completion date from 9 months post-diagnosis to the per-protocol 6 months changed the number of included patients. However, the estimates of routes to diagnosis and time intervals were not significantly altered and the trend was the same as in the main analyses (results not shown). Sensitivity analysis based solely on patient data for those whose reporting on all time intervals was complete did not change the trend (results not shown). Comparing patient and PCP reported routes (screening and symptomatic presentation) and registry data on screening showed that agreement ranged from moderate to almost perfect (Kappa 0.59-0.86).

Comparing the dates between the different data sources showed a high agreement between all data sources for all categories of dates (CCC $\geq$ 0.95 for date of diagnosis, CCC = 0.94 for date of treatment and CCC = 0.92 for date of first presentation to primary care). The analysis of the ICBP M4 definition of screening route compared with registry data showed an almost perfect agreement (Kappa>0.80) in two jurisdictions and substantial (Kappa>0.70) in two jurisdictions (Table 3).

For peer review only - http://bmjopen.bmj.com/site/about/guidelines.xhtml

DISCUSSION

Our study showed marked variation in the proportion of screen-detected cancers, lengths of diagnostic and treatment (and total) intervals between jurisdictions. Patient intervals were shortest in Denmark and longest in Wales; longer primary care intervals were present in Norway, Scotland, Manitoba and Victoria. Differences in primary care intervals do not necessarily reflect PCP delay - they may arise from PCPs undertaking more invesigations prior to referral. Overall, the differences are marked and suggest the need, in some jurisdictions, for revised diagnostic pathways to reduce the time taken for patients to be diagnosed and treated.

The interval differences did not show an obvious correlation with earlier reported survival differences –[4] jurisdictions with poorer survival did not consistently show longer intervals, and vice versa. While this may question the validity of our findings, and/or the relationship between diagnostic intervals and survival, it is important to note these analyses were several years apart, and there may have been significant health system changes since the survival comparisons. Nevertheless, our study adds to a growing body of evidence on routes to diagnosis and time intervals; there are few similar examples involving multiple countries in the literature.[29]

Many factors underpin the differences observed between jurisdictions, such as structural differences in healthcare delivery (e.g. care pathways, availability and accessibility of diagnostic and treatment facilities, etc.). Differences in routes to diagnosis were influenced by the presence or absence of screening programmes (in Denmark and Sweden CRC-screening had not been implemented at the time of study). Patient interval variation may reflect differences in symptom awareness or health-seeking behaviour. However, a study which compared awareness of cancer symptoms, attitudes towards cancer and barriers to attending a PCP did not demonstrated statistically significant differences.[30] It is possible that other factors, such as culture, rurality, economic or patient-specific barriers and facilitators, influence this important part of the pathway.[31]

There were significant differences in primary care intervals; overall these intervals were much longer than those found in our breast cancer analyses.[32] This raises important questions about diagnostic processes within primary care. There are widespread calls for PCPs to play a greater role in improving outcomes in CRC.[33] Indeed, it is now widely acknowledged that primary care has a major role in cancer control at all stages of the patient journey.[34] Nevertheless, there is some evidence based on observational associations that prolonged intervals might be associated with stonger 'gatekeeper' systems.[35] Further, independent of the nature of symptoms, investigation in primary care has been noted to be associated with later referral for specialist assessment for CRC and other cancers - so a long primary care interval may mean that PCPs are doing more before they refer[36]. Hence, PCP access to and use of investigations and differing national

For peer review only - http://bmjopen.bmj.com/site/about/guidelines.xhtml

cancer referral guidance may influence primary care intervals. There were differences in diagnostic intervals, suggesting that once patients have been referred to secondary care there is considerable variation in their experiences; differences in treatment intervals were less marked. These variations suggest there is room for improvement in reducing the total interval and its various sub-intervals, and that guidance on optimal pathways should be better implemented. Each participating jurisdiction will likely draw unique conclusions about the most appropriate response to our findings.

Strengths and weaknesses

A major strength of this study is its use of standardised survey methods in a broad range of jurisdictions to systematically examine the various components of these intervals and to describe and compare, between countries, patient journeys to a cancer diagnosis and treatment. To ensure comparability across jurisdictions, our surveys drew on existing instruments and went through an extensive process of cognitive testing, piloting and translation and adaptation.[18]

Data quality was enriched by information from national cancer registries and our algorithms showed very good agreement for jurisdictions where validation was possible. Using validated identification of CRC patients minimised the risk of missing cancer cases during inclusion and of selection bias. Further, the use of registries made it possible to exclude patients with previous cancer in the same site, providing a homogeneous group of newly diagnosed CRC patients in need of diagnostic work-up.

It was evident that there were subtle differences in the understanding of 'screening' between jurisdictions. Patients do not always distinguish between tests for screening and those for symptom-based diagnosis. Including data from registries and triangulating patient and PCP data enhanced the validity of 'screen-detected versus non-screen-detected' information, but the underlying factors varied between jurisdictions – for example, in Australia PCPs often provide screening FOBTs during consultations whereas this is rare in the UK and Scandinavia. To counter these inconsistencies, we applied our validated data rules which showed a high agreement with screening registries.

There are inevitable differences in questionnaire interpretation, characteristics of non-responders and availability of supplementary data for validation purposes. There are always considerations with questionnaire interpretation but the methodology and analysis of data sought to minimise or account for this as much as possible. Further, we used triangulation and comprehensive data rules to ensure validity, consistency and preserve statistical precision.[18] We included registry data where possible (screening, stage, date of diagnosis) and developed reliable rules for imputation based on these registry data. To minimise misclassification from data entry and handling, data entry was internally audited by local teams

For peer review only - http://bmjopen.bmj.com/site/about/guidelines.xhtml

and data interpretation was reduced to an absolute minimum and only performed centrally. Recall bias was minimised by the triangulation of different data sources and by ensuring that the patients received the questionnaire with a limited time window after the cancer diagnosis.

The overall response of 31% for the patient survey varied between jurisdictions. There were likely differences in the selection bias in individual jurisdictions; Our patient sampling strategy will have led to some differences in the composition of our samples, as some patients were included directly from registries, some via PCPs and, in Northern Ireland, research nurses checked lists of potentially eligible patients to confirm eligibility. We have no mechanism to examine the direction of such possible selection bias. However, comparison of participating patients on a number of variables (including comorbidity, self-assessed health, smoking, stage, presenting symptom) did not show obvious differences with potential to bias our results. We also compared age, sex and stage of cancer amongst participants versus eligible patients and found no significant differences. There were different classification systems for ethnicity and education across jurisdictions which would lead to biased estimates if included in the regression model, even if mapped or harmonised – hence, they were excluded. There were few respondents from minority ethnic groups, limiting the generalisability of our findings; further work should target these groups as they are likely to have unique characteristics in their routes to diagnosis.

Confounding from aspects related to the diagnostic route for CRC was diminished by adjusting for age, gender and comorbidity. It is possible that there is some residual confounding which can bias the results in different directions. The statistical precision of the study was high as we were able to show clinically significant differences of one week in time intervals.

Comparison with other studies

Other studies have examined symptoms and routes to diagnosis for patients with CRC – although rarely in more than a single setting. A UK study on patients diagnosed in 2001-2002 and 2007-2008 reported median diagnostic intervals of 100 and 80 days respectively.[37] A Danish study showed median diagnostic intervals for CRC of between 31 and 55 days, depending on the timing of measurement in relation to implementation of pathway guidance.[38] The difference between the present study and the former may stem from methodology issues, especially data sources (i.e. databases vs. surveys).

A study in Spain showed a symptom to diagnosis interval for CRC of 128 days and symptom to treatment interval of 155 days – these authors found that nature of symptoms, perceived seriousness of symptoms by patients, and place of first presentation influenced diagnostic and treatment intervals.[39] Sampling strategies and survey differences will have influenced the results, making it difficult to compare these

For peer review only - http://bmjopen.bmj.com/site/about/guidelines.xhtml

studies; nevertheless, they confirm that our results are broadly consistent with previous, single-jurisdiction studies.

The pattern of symptoms in ICBP participants was similar to other studies.[40] CRC is known to be a cancer that clinically presents with either 'alarm' symptoms, or more vague symptoms; there is evidence that doctors and patients respond less promptly to some symptoms of CRC than others – and that this can be influenced by the presence of co-morbid conditions.[41] However, the minor differences in symptom patterns seen in Table 4 are insufficient to explain the between-jurisdiction variation we've demonstrated in routes to diagnosis and diagnostic, treatment and total intervals.

Explaining observed differences between jurisdictions

The variation we see between jurisdictions mostly derives from differences in the extent to which healthcare systems support expedited CRC diagnosis and treatment; indeed, some health system characteristics, such as access and patient mobility between healthcare providers, may influence cancer outcomes - although these factors require further exploration.[42] In Denmark there have been a number of reforms specifically designed to reduce diagnostic intervals.[43] This study indicates a potential to optimise diagnostic routes for CRC in some jurisdictions. This should ideally be in conjunction with screening efforts which is gaining traction across many Western countries in response to policy and guideline initiaves.[44]

CONCLUSION

This study demonstrates considerable absolute and relative differences between jurisdictions in time intervals from first symptom until treatment for CRC. These differences do not demonstrate an obvious relationship with survival differences between the jurisdictions. The median total interval, which varied between 74 and 136 days, indicates that there is unrealised potential to optimise pathways for CRC. The main differences were found for structural parts of the pathway (e.g. those not relating to patient behaviours/actions). Further, there is a 'tail' of patients waiting many months longer to start treatment for their cancer which may affect their outcomes. While our study highlights important international differences in routes to diagnosis, further research is needed to understand these differences, and elucidate the contribution of patient pathway guidance and implementation, and health system structures. Nevertheless, the data provide important prompts for jurisdictions and suggest considerable room for improvement in some areas; they will also serve as a benchmark for measuring the effectiveness of future interventions.

For peer review only - http://bmjopen.bmj.com/site/about/guidelines.xhtml

List of abbreviations

ICBP M4 – International Cancer Benchmarking Partnership Module 4

PCP – primary care physician

CTS – cancer treatment specialist

CRC – colorectal cancer

Author's contribution

DW, PV, UM, AZF, HJ planned the study design, data collection, carried out the analyses and wrote the draft manuscript. All authors were responsible for local data collection, management and interpretation. All authors have participated in writing and have approved the final manuscript version.

Acknowledgements

We thank our colleagues and participating patients, primary care physicians and cancer treatment specialists in all participating ICBP jurisdictions. Catherine Foot, Martine Bomb, Brad Groves, Irene Reguilon and Samantha Harrison of Cancer Research UK for managing the programme. John Butler, Royal Marsden NHS Foundation Trust and ICBP Clinical Lead, for his advice. The ICBP M4 academic reference group (Supplementary File 2) for providing independent peer review and advice for the study protocol and analysis plan development. Throughout the study invaluable guidance was provide by the ICBP Programme Board. Membership of this board: Aileen Keel (Scottish Government, Edinburgh, Scotland); Anna Boltong (Cancer Council Victoria, Carlton, Australia); Anna Gavin (Northern Ireland Cancer Registry, Queens University, Belfast, UK); David Currow (Cancer Institute New South Wales, Sydney, Australia); Gareth Davies (Wales Cancer Network, Cardiff, UK); Gunilla Gunnarsson (Swedish Association of Local Authorities and Regions, Stockholm, Sweden); Heather Bryant (Canadian Partnership Against Cancer, Toronto, Canada); Jane Hanson (Welsh Cancer National Specialist Advisory Group, Cardiff, UK); Kathryn Whitfield (Department of Health, Victoria Australia); Linda Rabeneck (Cancer Care Ontario, Toronto, Canada); Michael A Richards (Care Quality Commission, London, UK); Michael Sherar (Cancer Care Ontario, Toronto, Canada); Nicola Quin (Cancer Council Victoria, Carlton, Australia); Nicole Mittmann (Cancer Care Ontario, Toronto, Canada); Robert Thomas (Department of Health and Human Services, Victoria, Melbourne, Australia); Sara Hiom (Cancer Research UK); Sri Navaratnam (Cancer Care Manitoba, Winnipeg, Canada); Sean Duffy (NHS England, London, UK); Chris Harrison (NHS England, London, UK); Søren Brostrøm (Danish Health and Medicines Authority, Copenhagen, Denmark); Stein Kaasa (University Hospital of Trondheim, Trondheim, Norway).

Competing Interests

None

Funding

For peer review only - http://bmjopen.bmj.com/site/about/guidelines.xhtml

This work was supported by: CancerCare Manitoba; Cancer Care Ontario; Canadian Partnership Against Cancer (CPAC); Cancer Council Victoria; Cancer Research Wales; Cancer Research UK; Danish Cancer Society; Danish Health and Medicines Authority; European Palliative Care Research Centre (PRC), Norwegian University of Science and Technology (NTNU); Guidelines Audit and Implementation Network (GAIN); Macmillan Cancer Support; National Cancer Action Team; NHS England; Northern Ireland Cancer Registry, funded by the Public Health Agency NI; Norwegian Directorate of Health; Research Centre for Cancer Diagnosis in Primary Care (CaP), Aarhus University, Denmark; Scottish Government; Swedish Association of Local Authorities and Regions; University College London and NIHR Biomedical Research Centre at University College London NHS Foundation Trust; University of Edinburgh; Victorian Department of Health and Human Services; Welsh Government. The funding bodies had no influence on the design of the study and collection, analysis, and interpretation of data, in writing the manuscript or whether to publish the results.

Availability of data and material

The data that support the findings of this study are available from the named authors from each ICBP jurisdiction, but restrictions apply to the availability of these data and so are not publicly available. Data are however available from the authors upon reasonable request and with permission of the ICBP Programme Board. Please contact the ICBP Programme Management team, based at Cancer Research UK, with any queries (icbp@cancer.org.uk).

Ethics approval and consent to participate

For each local data collection, there were specific procedures and approvals which included anonymised data transfer to University College London and Aarhus University (Supplementary file 5). Approvals were received from the following institutions: Cancer Council Victoria Human Research Ethics Committee [HREC 1125]; Health Research Ethics Board, University of Manitoba [HS15227 (H2012:105)]; Research Resource Ethics Committee, CancerCare Manitoba [RRIC#28-2012]; University of Toronto Research Ethics Board [27881]; The Danish Data Protection Agency [2013-41-2030]; Swedish Ethics Review Board, Uppsala [2013/306]; Norway Regional committees for medical and health research ethics [2013/136/REK nord]; England, Wales and Scotland, NRES Committee East Midlands – Derby 2, local R&D for each health board

For peer review only - http://bmjopen.bmj.com/site/about/guidelines.xhtml

[11/EM/0420]; Northern Ireland ORECNI Ethical approval, local governance for each health Trust [11/EM/0420].

For peer review only

For peer review only - http://bmjopen.bmj.com/site/about/guidelines.xhtml

References

1 Ferlay J, Soerjomataram I, Dikshit R, et al. Cancer incidence and mortality worldwide: sources, methods and major patterns in GLOBOCAN 2012. *International journal of cancer* 2015 Mar 1;136(5):E359-86. doi: 10.1002/ijc.29210

2 Brenner H, Chang–Claude J, Jansen L, et al. Reduced risk of colorectal cancer up to 10 years after screening, surveillance, or diagnostic colonoscopy. *Gastroenterology* 2014 Mar 31;146(3):709-17. doi: 10.1053/j.gastro.2013.09.001.

3 De Angelis R, Sant M, Coleman MP, et al. Cancer survival in Europe 1999–2007 by country and age: results of EUROCARE-5—a population-based study. *Lancet Oncol* 2014 Jan 31;15(1):23-34. doi: 10.1016/S1470-2045(13)70546-1

4 Coleman MP, Forman D, Bryant H, et al. Cancer survival in Australia, Canada, Denmark, Norway, Sweden, and the UK, 1995-2007 (the International Cancer Benchmarking Partnership): an analysis of population-based cancer registry data. *Lancet* 2011;377:127-38 doi:10.1016/S0140-6736(10)62231-3

5 Maringe C, Walters S, Rachet B, et al. Stage at diagnosis and colorectal cancer survival in six high-income countries: a population-based study of patients diagnosed during 2000-2007. *Acta Oncol* 2013;52(5):919-32. doi: 10.3109/0284186X.2013.764008

6 Butler J, Foot C, Bomb M, et al. The international cancer benchmarking partnership: an international collaboration to inform cancer policy in Australia, Canada, Denmark, Norway, Sweden and the United Kingdom. *Health Policy* 2013 Sep 30;112(1):148-55. doi:10.1016/j.healthpol.2013.03.021

7 Hamilton W. Cancer diagnosis in primary care. *Br J Gen Pract* 2010 Feb 1;60(571):121-8. doi: 10.3399/bjgp10X483175.

8 Elliss-Brookes L, McPhail S, Ives A, et al. Routes to diagnosis for cancer - determining the patient journey using multiple routine data sets. *Br J Cancer* 2012 Oct 9;107(8):1220-6. doi: 10.1038/bjc.2012.408.

9 Hansen RP, Vedsted P, Sokolowski I, et al. Time intervals from first symptom to treatment of cancer: a cohort study of 2,212 newly diagnosed cancer patients. *BMC Health Serv Res* 2011 Oct 25;11(1):1. doi: 10.1186/1472-6963-11-284.

10 Emery JD, Shaw K, Williams B, et al. The role of primary care in early detection and follow-up of cancer. *Nat Rev Clin Oncol* 2014 Jan;11(1):38-48. doi: 10.1038/nrclinonc.2013.212.

11 Vedsted P, Olsen F. Early diagnosis of cancer-the role of general practice. *Scand J Prim Health Care* 2009;27(4):193-4. doi: 10.3109/02813430903478623.

12 Jensen H, Tørring ML, Olesen F, et al. Cancer suspicion in general practice, urgent referral and time to diagnosis: a population-based GP survey and registry study. *BMC Cancer* 2014 Aug 30;14:636. doi: 10.1186/1471-2407-14-636. doi: 10.1186/1471-2407-14-636

13 Tørring ML, Frydenberg M, Hamilton W, et al. Diagnostic interval and mortality in colorectal cancer: U-shaped association demonstrated for three different datasets. *J Clin Epidemiol* 2012;65(6):669-78. doi: 10.1016/j.jclinepi.2011.12.006

14 Neal RD, Tharmanathan P, France B, et al. Is increased time to diagnosis and treatment in symptomatic cancer associated with poorer outcomes; Systematic review. *Br J Cancer* 2015 Mar 31;112:S92-107. doi: 10.1038/bjc.2015.48.

For peer review only - http://bmjopen.bmj.com/site/about/guidelines.xhtml

For peer review only

15 Rose PW, Rubin G, Perera-Salazar R, et al. Explaining variation in cancer survival between 11 jurisdictions in the International Cancer Benchmarking Partnership: a primary care vignette survey. *BMJ Open* 2015 May 1;5(5):e007212. doi: 10.1136/bmjopen-2014-007212

16 Brenner H, Bouvier AM, Foschi R, et al. Progress in colorectal cancer survival in Europe from the late 1980s to the early 21st century: the EUROCARE study. *International Journal of Cancer* 2012 Oct 1;131(7):1649-58. doi: 10.1002/ijc.26192

17 Cancer Research UK, http://www.cancerresearchuk.org/health-professional/cancer-statistics/statistics-by-cancer-type/bowel-cancer/diagnosis-and-treatment#heading-Zero, Accessed January 2018.

18 Weller D, Vedsted P, Anandan C, et al. An investigation of routes to cancer diagnosis in 10 international jurisdictions, as part of the International Cancer Benchmarking Partnership: survey development and implementation. *BMJ Open* 2016 Jul 1;6(7):e009641. doi: 10.1136/bmjopen-2015-009641

19 Weller D, Vedsted P, Rubin G, et al. The Aarhus statement: improving design and reporting of studies on early cancer diagnosis. *Br J Cancer* 2012 Mar 27;106(7):1262-7. doi: 10.1038/bjc.2012.68

20 World Health Organisation. International Classification of Diseases and Related Health Problems (ICD-10). WHO, Geneva, 2016

21 European Network of Cancer Registries. Standards and guidelines for cancer registration in Europe: the ENCR recommendations. Edited by Tyczynski JE, Démaret E, Parkin DM. (IARC technical publication; no. 40), Lyon, 2003

22 Sobin LH, Fleming ID. TNM classification of malignant tumors, (1997). *Cancer* 1997 Nov 1;80(9):1803-4

23 Jenkinson C, Coulter A, Wright L. Short form 36 (SF36) health survey questionnaire: normative data for adults of working age. *BMJ* 1993 May 29;306(6890):1437-40

24 Nicholson BD, Mant D, Neal RD, et al. International variation in adherence to referral guidelines for suspected cancer: a secondary analysis of survey data. *Br J Gen Pract* 2016 Feb 1;66(643):e106-13. doi: 10.3399/bjgp16X683449.

25 Koenker R, Bassett G. Regression quantiles. *Econometrica* 1978; 46:33-50

26 Miranda A. QCOUNT: Stata program to fit quantile regression models for count data. Boston: Boston College Department of Economics; 2006

27 Landis J, Koch G. The measurement of observer agreement for categorical data. *Biometrics* 1997;33(1): 159-174

28 Lin LI. A concordance correlation coefficient to evaluate reproducibility. *Biometrics* 1989;45((1) March):255–68

29 Murchie P, Campbell NC, Delaney EK, et al. Comparing diagnostic delay in cancer: a cross-sectional study in three European countries with primary care-led health care systems. *Family Practice* 2012, *29*(1), 69-78. doi: 10.1093/fampra/cmr044

30 Forbes LJ, Simon AE, Warburton F, et al. Differences in cancer awareness and beliefs between Australia, Canada, Denmark, Norway, Sweden and the UK (the International Cancer Benchmarking Partnership): do they contribute to differences in cancer survival? *Br J Cancer* 2013;108:292-300 doi:10.1038/bjc.2012.542

For peer review only

For peer review only - http://bmjopen.bmj.com/site/about/guidelines.xhtml

31 Parsonage RK, Hiscock J, Law RJ, et al. Patient perspectives on delays in diagnosis and treatment of cancer: a qualitative analysis of free-text data. *Br J Gen Pract* 2017; 67 (654): e49-e56. DOI: 10.3399/bjgp16X688357

32 Vedsted P, Weller D, Falborg AZ, et al. Diagnostic routes and time intervals for breast cancer in 10 jurisdictions. The international cancer benchmarking partnership (ICBP) 2018 (undergoing submission process)

33 Rubin G, Weller D. Improving survival in colorectal cancer: what role for general practice? *Br J Gen Pract* 2013 Apr 1;63(609):179-80

34 Rubin G, Berendsen A, Crawford SM, et al. The expanding role of primary care in cancer control. *Lancet Oncol* 2015 Sep 30;16(12):1231-72 - a

35 Vedsted P, Olesen F. Are the serious problems in cancer survival partly rooted in gatekeeper principles? An ecologic study. *Br J Gen Pract* 2011 Aug 1;61(589):e508-12

36 Rubin GP, Saunders CL, Abel GA, et al. Impact of investigations in general practice on timeliness of referral for patients subsequently diagnosed with cancer: analysis of national primary care audit data. British journal of cancer 2015 Feb 17;112(4):676-87 – b

37 Neal RD, Din NU, Hamilton W, et al. Comparison of cancer diagnostic intervals before and after implementation of NICE guidelines: analysis of data from the UK General Practice Research Database. *Br J Cancer* 2014 Feb 4;110(3):584-92.

38 Jensen H, Tørring ML, Olesen F, et al. Diagnostic intervals before and after implementation of cancer patient pathways–a GP survey and registry based comparison of three cohorts of cancer patients. *BMC cancer* 2015 Apr 23;15(1):1

39 Esteva M, Leiva A, Ramos M, et al. Factors related with symptom duration until diagnosis and treatment of symptomatic colorectal cancer. *BMC cancer* 2013 Feb 23;13(1):1

40 Astin M, Griffin T, Neal RD, et al. The diagnostic value of symptoms for colorectal cancer in primary care: a systematic review. *Br J Gen Pract* 2011 May 1;61(586):e231-43

41 Walter FM, Emery J, Mendonca S, et al. Symptoms and co-morbidities associated with diagnostic intervals for colorectal cancer: a prospective cohort study. *Eur J Cancer Care* 2015 Sep 1;24:49

42 Brown S, Castelli M, Hunter DJ, et al. How might healthcare systems influence speed of cancer diagnosis: a narrative review. *Soc Sci Med* 2014 Sep 30;116:56-63

43 Vedsted P, Olesen F. A differentiated approach to referrals from general practice to support early cancer diagnosis–the Danish three-legged strategy. *Br J Cancer* 2015 Mar 31;112(Suppl 1):S65.

44 Altobelli E, Lattanzi A, Paduano R, et al. Colorectal cancer prevention in Europe: burden of disease and status of screening programs. *Prev Med2014* May 31;62:132-41

For peer review only

For peer review only - http://bmjopen.bmj.com/site/about/guidelines.xhtml

Figure 1 – Survival differences for colorectal cancer demonstrated in the International Cancer Benchmarking Partnership Module 1.[4]

Figure 2 – Diagnosis and treatment of colorectal cancer: Illustration of key time points and intervals.[19]

Supplementary File 1 – Data rules

Supplementary File 2 – Sample CRC patient questionnaire

Supplementary File 3 – Sample CRC primary care questionnaire

Supplementary File 4 – Sample CRC specialist questionnaire

Supplementary File 5 – Ethical approval, Working and Academic Reference Groups

Supplementary File 6 – Regression analysis diagrams (based on Table 6)

For peer review only

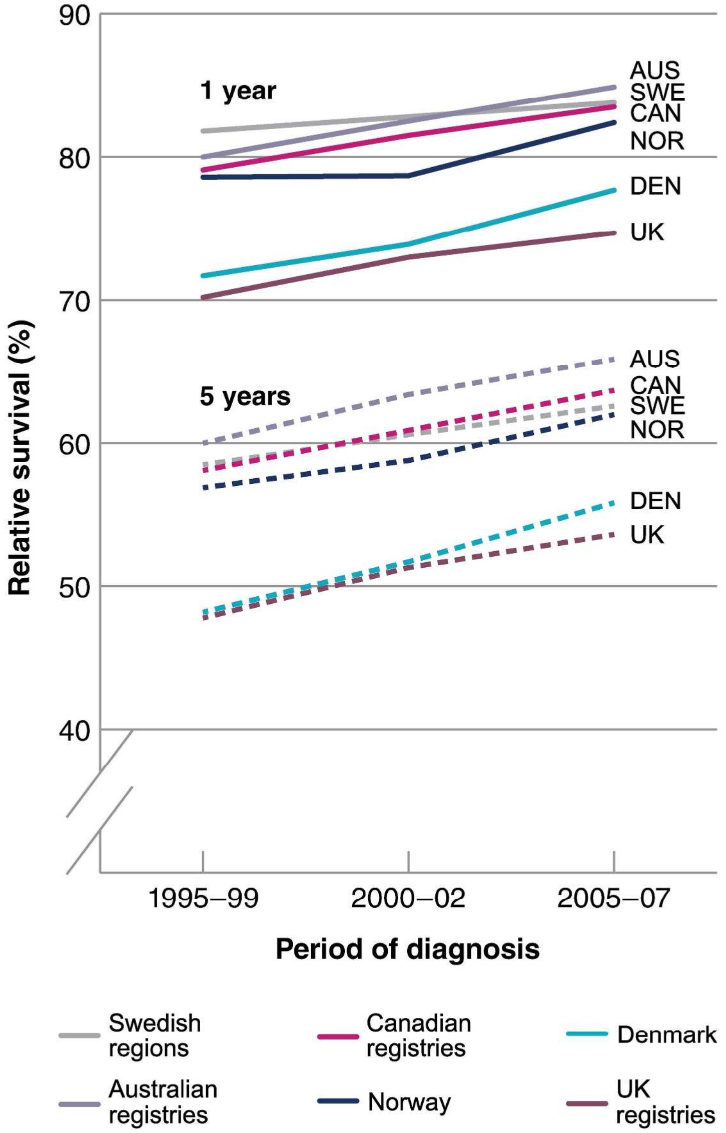

206x312mm (300 x 300 DPI)

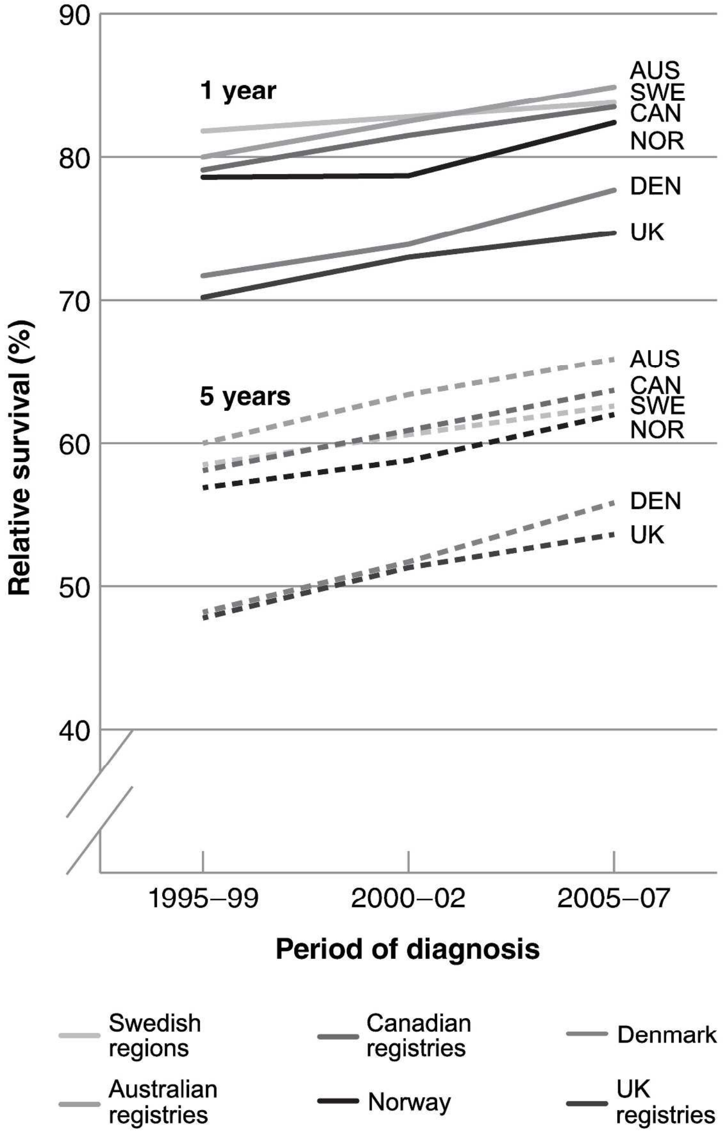

206x312mm (300 x 300 DPI)

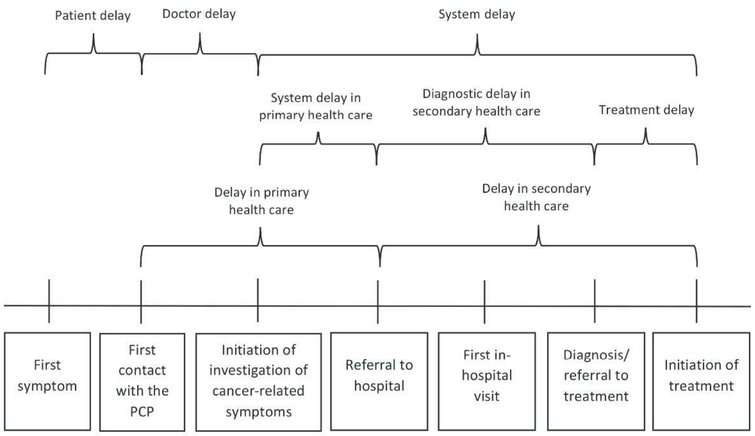

209x297mm (300 x 300 DPI)

Supplementary file 1: ICBPM4 Rules for missing, incomplete, multiple response and out of range data

1. Oversampling/Participation in local screening trials
   a) To handle oversampling in Ontario, include only the first 360 consecutive CRC patients;
   b) In jurisdictions with no national screen program: exclude patients participated in local screen trials.

2. Language/Participation in study/Presence of cancer
   Exclude patients who checked "No, I don't understand the language" or "I don't want to participate in this study" or "I don't have cancer".

3. Survey responders
   a) Exclude Patient/PCP/Specialist survey from the analysis, if it was not written by Patient/PCP/Specialist (example: a medical oncologist completed a PCP survey);
   b) In the case of duplicates, include only the first survey (example: 2 specialists completed surveys for the same patient).

4. Gender
   Exclude patients with unknown Gender.

5. Age
   a) Exclude patients with unknown age;
   b) Exclude patients younger 40 years;
   c) Use registry data, if Age is reported by both patient and registry.

6. No cancer or Previous cancer in the same organ
   a) Exclude patients with no cancer based on registry data;
   b) Exclude patients with previous cancer in the same organ based on data from registry or free-text for Presentation in the patient survey.

7. Date of consent
   Exclude patients with date of consent which is unknown, before 01.01.2013 or in the future.

8. Multiple responses to Dates
   If multiple responses were given to the dates (of first symptom; screening; first presentation to primary care; referral; diagnosis; treatment start), then use the earliest date.

9. Order of Dates
   The dates must be in the following order –
   a) First symptom; first presentation to Primary Care; referral; diagnosis; treatment start.
   b) Screening; diagnosis; treatment start.

   If not, check for mistakes.

10. Date of first symptom
    Date of first symptom is defined as date of first symptom from patient data.

11. Date of first presentation
    Date of first presentation to Primary Care is defined as (in the order of declining priority):
    a) date of first presentation to Primary Care from PCP data;
    b) date of first presentation to Primary Care and A&E from PCP data;
    c) date of first presentation to Primary Care from patient data.

For peer review only

12. Date of referral
Date of referral is defined as date of referral from PCP data.

13. Date of screening
Date of screening is defined as (in the order of declining priority):
a) date of screening from registry;
b) date of screening from patient data.

14. Date of diagnosis
Definition
a) If Registry reports both date of histological confirmation and date of confirming investigation, then use date of histological confirmation.
b) Date of diagnosis (based on patient data, PCP data, specialist data, registry data) is defined as (in the order of declining priority):
- date of diagnosis from registry;
- date of histological confirmation (from specialist data, PCP data);
- date of biopsy (from specialist data, PCP data);
- date of confirming investigation (from specialist data, PCP data);
- date of first hospital admission (from specialist data, PCP data);
- date of MDT confirmation (from specialist data, PCP data);
- date patient was told (from specialist data, PCP data);
- other date of diagnosis (from specialist data, PCP data, patient data);

Choose a Date from a lower level of hierarchy, if the Date from a higher level is after the Date of consent or more than 9 months (=271 days) before the Date of consent.

Exclusion criteria
a) Unknown date of diagnosis;
b) Date of diagnosis is after the date of consent;
c) Date of diagnosis is more than 9 months before the Date of consent.

15. Date of treatment start
a) Date of treatment start from patient data is defined as the earliest of the treatment dates for Surgery, Chemo, Radio and Other;
b) Date of treatment start (based on registry data, specialist data, patient data) is defined as (in the order of declining priority):
- date of treatment start from registry data,
- date of treatment start from specialist data,
- date of treatment start from patient data,
- anticipated date of treatment from patient data.

16. Imputation of missing day in the date
Imputation rules for missing day (given month and year are known):
a) Set missing day to '16';
b) Consider adjacent dates in a backwards order (from "Treatment" to "First symptom"). For each pair of such adjacent dates: If dates are not in a logical order (e.g. "Treatment" is before "Diagnosis"), but month and year are the same in both dates, and the day was imputed to '16' in one of the dates:
- Recode the day imputed earlier to '16' to the day from the adjacent date.

17. Considering time
If patient gave multiple answers to the "How long did you have symptoms before contacting a doctor?" question, then use the option with the shortest time interval.

18. Delay arranging appointment
If patient gave multiple answers to the "How long did it take to get an appointment with PCP?" question, then

For peer review only - http://bmjopen.bmj.com/site/about/guidelines.xhtml

use the option with the shortest time interval.

19. Duration of symptoms
If PCP gave multiple answers to the "Duration of symptoms" question, then use the option with the shortest time interval.

20. Definition of Presentation

A. Define Presentation within a Data Source (Patient, PCP)

1. Review the free-text for Presentation (Patient, PCP) and re-code, if possible.
2. If PCP reports 'VisitPCP and AE' or 'VisitPCP' as Presentation and no symptoms, then check Patient's records. If Patient reports 'Screening' and no symptoms, then re-code Presentation for this case as 'Screening'.
3. If PCP reports 'Screening' as Presentation and at least one symptom (or "Duration of Symptoms"), then re-code Presentation to 'Other non-screen-detected'-option.
4. If PCP reports 'Other' as Presentation and at least one symptom (or "Duration of Symptoms"), then re-code Presentation to 'Other non-screen-detected'-option.
5. If Patient reports 'Screening' as Presentation and at least one symptom (or date of first symptom), then re-code Presentation to 'Other non-screen-detected'- option.
6. If Patient reports 'Other' as Presentation and at least one symptom (or date of first symptom or "Considering time" or "Delay arranging appointment", then re-code Presentation to 'Other non-screen-detected'-option.
7. In the case of multiple Presentation responses (Patient, PCP sources) - use a single option (in the order of declining priority):

   a) 'VisitPCP and AE',
   b) 'VisitPCP´, 'AE´ (if both 'VisitPCP´ and 'AE´ are given, then re-code as 'VisitPCP and AE'),
   c) 'Other non-screen-detected',
   d) 'Screening',
   e) 'Investigation for another problem' ,
   f) 'Other"

B. Define Presentation from Alternative Data

If Presentation hasn't been reported in either of data sources, then define it as (in the order of declining priority):

1. 'Other non-screen-detected ', if PCP reports at least one symptom (or "Duration of symptoms");
2. 'Other non-screen-detected ', if Patient reports at least one symptom (or date of first symptom);
3. 'Other non-screen-detected ', if Patient reports "Considering time" or "Delay arranging appointment" and no screening date;
4. 'Screening', if Patient reports screening date and no symptoms and no date of first symptom;
5. 'Other non-screen-detected ', if jurisdiction=England, Age <58 or >76 years.

C. Define Presentation from Data Source Hierarchy

1. In Wales, England, Scotland, N Ireland and Manitoba: if Registry reports 'Screening' – use Presentation data from Registry data.

2. In Wales, England, Scotland, N Ireland and Manitoba: if Registry reports 'No Screening' – use Presentation data from (in the order of declining priority):
   a) PCP data;
   b) Patient data;

   If PCP (or Patient, in the case of PCP data is not available) reports 'Screening', then code Presentation as 'Other non-screen-detected'. If information from PCP and Patient datasets is missing, then code Presentation as 'Other non-screen-detected'.

3. In Wales, England, Scotland, N Ireland and Manitoba: if screening status from Registry is missing – use Presentation data from (in the order of declining priority):

For peer review only - http://bmjopen.bmj.com/site/about/guidelines.xhtml

a) PCP data;
b) Patient data;

4. For Denmark, Norway, Ontario and Victoria – use Presentation data from (in the order of declining priority):
   a) PCP data;
   b) Patient data.

5. In Sweden – use Presentation data from Patient data.

21. Patient interval
The Patient interval for non-screen-detected patients is defined as (in the order of declining priority):
a) "Date of first presentation to Primary Care" minus "Date of first symptom";
b) If the interval in (a) is unknown or negative: Calculate the interval as the low boundary of "Considering time" plus the low boundary of "Delay arranging appointment";
c) If the interval in (a) is unknown or negative and the interval in (b) is unknown: Calculate the interval as the low boundary of "Duration of symptoms interval".

22. Primary Care interval
The Primary Care interval for non-screen-detected is defined as "Date of referral" minus "Date of first presentation to Primary Care".

23. Diagnostic interval
a) The Diagnostic interval for non-screen-detected is defined as "Date of diagnosis" minus "Date of first presentation to Primary Care";
b) The Diagnostic interval for screen-detected patients is defined as "Date of diagnosis" minus "Date of screening".

24. Treatment interval
The Treatment interval is defined as "Date of treatment start" minus "Date of diagnosis".

25. Total interval
a) The Total interval for non-screen-detected patients is defined as "Date of treatment start" minus "Date of first symptom";
b) The Total interval for screen-detected patients is defined as "Date of treatment start" minus "Date of screening".

26. Range of Time intervals
The time intervals (Patient, Primary Care, Diagnosis, Treatment, Total) must be in range 0-1 year.

If > 1 year: set the interval to 365 days
If negative: set the interval to 0.

For each jurisdiction calculate the number of imputations due to:
a) unknown day in a date (given known month and year);
b) very large(>1 year) interval;
c) negative interval.

27. Type of treatment
If patient ticked both "Yes" and "No" as answers to the "Type of treatment (Surgery, Chemotherapy, Radiotherapy)" questions, then choose "Yes" answer.

28. Health state
If patient gave multiple answers to the "Health state" question, then use the option with a better health condition.

For peer review only - http://bmjopen.bmj.com/site/about/guidelines.xhtml

29. Comorbidity
    a) If patient ticked both “Yes” and “No” as answers to the “Comorbidity (Heart disease, Stroke, Lung disease, Diabetes)” questions, then choose “Yes” answer;
    b) If both patient and PCP report “Comorbidity”, then use the PCP Data.

30. Ethnicity
    a) If patient didn’t report “Ethnicity”, then use the information from (in the order of declining priority):
        - “Ethnicity_Other_Details”;
        - “Other main language spoken at home”;
        - “The main language spoken at home” (only for Victoria);
        - “The main language spoken at home is the chief one for this jurisdiction”=”Yes” given “Main language spoken at home is other than the main one for this jurisdiction”=”No”;
    b) Consider Ethnicity as unknown, if answers to the “Ethnicity” question are multiple and belong to different categories (‘white’, ‘Asian’, ‘black’, ‘other’).

31. Education
    If patient gave multiple answers to the “Education” question, then use the option with a higher level of education.

32. Smoking Current
    a) If patient ticked both “Yes” and “No” as answers to the “Smoking Current” question, then use “Yes” answer;
    b) If patient hasn’t ticked neither “Yes” nor “No, then consider this case as Unknown.

33. Smoking Number
    If patient reports “SmokingNumber” as text, then re-code using following rules:

    a) Where there is a number smoked /day – accept number;
    b) Where a range has been given – take the upper value;
    c) Where patient has put 10+ or 20+ - capture this as 11 or 21;
    d) Where number of cigarettes smoked in the past and currently being smoked are provided - average the numbers;
    e) Non entries code as “.” ;
    f) Non-smokers (eg, “nil”, “N/A“) are coded as “0”.

34. Smoked ever
    a) If patient ticked both “Yes” and “No” as answers to the “Smoking ever” question, then use “Yes” answer;
    b) If patient hasn’t ticked neither “Yes” nor “No”: consider it as “Yes”, if patient is a current smoker (“Smoking_Current=”Yes””) or has specified a number of cigarettes (“SmokingNumber”>0). Otherwise consider this case as Unknown.
    c) If patient has ticked “No”: recode it to “Yes”, if patient is a current smoker (“Smoking_Current=”Yes”).

35. Nature of referral
    a) Review free-text for “ Nature of referral” (PCP Data) and re-code, if possible;
    b) In the case of multiple responses, use a single option as (in the order of declining priority):

        - “Referral for immediate admission”;
        - “Urgent referral”;
        - “Less urgent referral”;
        - “General referral” ;
        - “No referral”;
        - “Other”.

For peer review only

36. Stage-TNM

a) If specialist gave multiple responses to the “Stage_TNM” question, then use the highest category;
b) If registry gave multiple responses to the “Stage_TNM”, then use a single option (in the order of declining priority):
   - stage at time of diagnosis
   - stage at surgery
   - stage at oncology
c) If “Stage_TNM” is reported by both the specialist and registry, then use the registry data;
d) If “Stage_TNM” is unknown or “not able to stage”, then use “Stage_Duke”.

37. Stage Dukes

a) If specialist gave multiple responses to the “Stage_Dukes’” question, then use the highest category;
b) If “Stage_Dukes” is reported by both the specialist and registry, then use the registry data.

For peer review only

ICBP
International Cancer
Benchmarking Partnership

International Cancer Benchmarking Partnership Module 4

Patient questionnaire

Colorectal Cancer

Thank you very much for taking the time to fill in this questionnaire – it should take about 20 minutes to complete. We are sending the questionnaire to a large sample of people who we understand have had a diagnosis of colorectal cancer. If this has been sent to you in error and you do not have cancer, please do not continue and return the documents in the prepaid envelope.

Sample

Our aim is to gain a better understanding of the process by which people have their cancer diagnosed. We would also like to find out more about the symptoms they experience (if any), and the pathway they follow from start of symptoms to treatment of their cancer. This will help in identifying ways in which cancers can be diagnosed quickly and effectively. Thank you once again for your time.

This information is confidential and will not be passed to anyone involved in your treatment.

Name: ______________________

Date of Birth: ______________________

Address: ______________________

____

____

Copyright property of the International Benchmarking Partnership (ICBP) and Module 4 collaborators. Usage must be authorised

International Cancer
Benchmarking Partnership

Consent form

Please read the consent form and sign your name and date **BELOW**.

If you require any clarification, please do not hesitate to ring the study team members. Their contact details are found on the information sheet.

Please be reassured that your responses are completely confidential and will not be passed to anyone involved in your treatment. For the purposes of the study it is important that you agree to consent to all the statements listed below.

- I confirm that I have read the attached information sheet and I understand why the research is being done.
- I am willing for the team to request information from my GP and hospital doctors which is relevant to the audit as described in the information sheet.
- I give permission for my details (name, address) to be given to the cancer registry (NHS Information Centre for Health and Social Care) for follow up.
- I agree for the information I have provided and any other relevant information from my medical records to be stored as described in the information sheet under the custodianship of University College London.
- I consent to sharing of coded data which contains no personal identifiers between researchers, some of whom are located outside the European Union.
- I consent for use of my data if I become mentally incapacitated during the course of the project.

I agree to all the statements listed and consent to participate in the study.

Name (Please print)

Signature: Date:

If we have any questions, may we phone you for clarification? (Please tick) ☐ **Yes** ☐ **No**

If **Yes**, please provide your telephone number:

Copyright property of the International Benchmarking Partnership (ICBP) and Module 4 collaborators. Usage must be authorised.

ICBP
International Cancer
Benchmarking Partnership

1. **Please can you confirm the details of your GP/GP practice (name, practice address – as best as you can remember): We appreciate that you may have more than one GP involved in your care – in which case, we are interested in the GP you would say provides the majority of your care, particularly relating to the cancer you've had diagnosed.**

Name of doctor

Name of practice

Address

Postcode

Town

Sample

Copyright property of the International Benchmarking Partnership (ICBP)
and Module 4 collaborators. Usage must be authorised

For peer review only - http://bmjopen.bmj.com/site/about/guidelines.xhtml

ICBP
International Cancer Benchmarking Partnership

2. Which of the following best describes the events which led to your diagnosis of cancer? (please tick only **ONE** answer)

	✓
I had symptoms/I noticed a bodily change and went to see a doctor (e.g. GP)	
I had symptoms/I noticed a bodily change and went/was taken to Accident and Emergency (A&E)	
I had seen a doctor/GP with symptoms, but went/was taken to Accident and Emergency (A&E) when things worsened	
I was being investigated by my doctor(s) for another problem during which time the cancer was discovered	
I had a cancer screening test as part of a colorectal screening programme (e.g. the NHS Bowel Cancer Screening Programme in England – NHS BCSP)	
Other (please describe):	

Sample

What date did you have this screening test? If you cannot remember the exact date, you can fill in the month and the year.

Day (optional), month, year

D	D	M	M	Y	Y	Y	Y

Other (please describe):

Copyright property of the International Benchmarking Partnership (ICBP) and Module 4 collaborators. Usage must be authorised

ICBP
International Cancer
Benchmarking Partnership

3. The following health concerns or symptoms are commonly experienced with colorectal cancer.

Rectal bleeding or blood in faeces (poo)
Change in bowel habit
Unexplained tiredness/fatigue
Unexplained weight loss
Pelvic or abdominal pain
Skin went yellow
Abdominal distension/increased abdominal size/persistent bloating

Sample

Please write down ALL health concern(s) or symptom(s) you may have had before contacting a doctor or taking part in screening. It does not matter if they are not included in the list above:

Please write your health concern(s) or symptom(s) in the boxes below:
1)
2)
3)
4)
5)
6)

	✓
This is not applicable to me (e.g. I did not have any symptoms), please tick	

Copyright property of the International Benchmarking Partnership (ICBP)
and Module 4 collaborators. Usage must be authorised

ICBP
International Cancer Benchmarking Partnership

4. Please write down your best estimate of the date you noticed the first of these health concern(s) or symptom(s). If you cannot remember the exact date, you can fill in the month and the year.

Day (optional), month, year

D	D	M	M	Y	Y	Y	Y

✓

This is not applicable to me (e.g. I had no symptoms), please tick	

5. Approximately how long did you have health concern(s) or symptom(s) before contacting a doctor? (Please think of the first visit to the doctor, not re-visits after that). Please tick only **ONE** answer.

✓

Less than 1 week	
1-2 weeks	
3-4 weeks	
5-7 weeks	
2-5 months	
6-12 months	
More than 12 months	

Sample

✓

This is not applicable to me (e.g. I had no symptoms), please tick	

Copyright property of the International Benchmarking Partnership (ICBP)
and Module 4 collaborators. Usage must be authorised

ICBP
International Cancer
Benchmarking Partnership

6a. Once you contacted a practice about your health concern(s) or symptom(s), how long did it take to get an appointment with a doctor? (Please think of the first visit to the doctor, to discuss your health concern(s) or symptom(s)).
Please tick only **ONE** answer.

	✓
Same day/next day	
Within 1 week	
1-2 weeks	
3-4 weeks	
Longer	

If there was no waiting time (e.g. you went/were taken to A&E), please tick this box	

This is not applicable to me (e.g. I had no symptoms), please tick	

Sample

6b. What was the date you first saw your doctor about your health concern(s) or symptom(s)? If you cannot remember the exact date, you can fill in the month and the year.

Day (optional), month, year

D	D	M	M	Y	Y	Y	Y

	✓
This is not applicable to me (e.g. I had no symptoms), please tick	

Copyright property of the International Benchmarking Partnership (ICBP) and Module 4 collaborators. Usage must be authorised

ICBP
International Cancer
Benchmarking Partnership

7. How many times did you visit the following for the investigation of your symptoms before your cancer was diagnosed?

	Please write down the number of visits
GP	
Hospital	
Consultant/specialist outside of a hospital	

	✓
This is not applicable to me (e.g. I had no symptoms)	

8a. After your doctor referred you to a specialist, how long did it take you to get an appointment? Please tick only **ONE** answer.

	✓
Less than 1 week	
1-2 weeks	
3-4 weeks	
5-7 weeks	
2-5 months	
6-12 months	
More than 12 months	

	✓
This is not applicable to me (eg my doctor did not refer me), please tick	

Sample

Copyright property of the International Benchmarking Partnership (ICBP) and Module 4 collaborators. Usage must be authorised

ICBP
International Cancer
Benchmarking Partnership

8b. What was the date of your first appointment with a doctor, involved in investigating and/or treating your cancer, to whom you were referred?
If you cannot remember the exact date, you can fill in the month and the year.

Day (optional), month, year

D	D	M	M	Y	Y	Y	Y

This is not applicable to me (e.g. my doctor did not refer me), please tick	

9. What was the date you were told you had cancer? If you cannot remember the exact date, you can fill in the month and the year.

Day (optional), month, year

D	D	M	M	Y	Y	Y	Y

Sample

Copyright property of the International Benchmarking Partnership (ICBP)
and Module 4 collaborators. Usage must be authorised

ICBP
International Cancer
Benchmarking Partnership

10. Have you had any of the following treatments for your cancer yet? If so, please can you estimate the date this treatment started? Please tick **ALL** that apply. If you cannot remember the exact date, you can fill in the month and the year.

	**Type of treatment**		**Date of treatment** (give first date if you had more than one)
a.	Surgery	☐ **Yes** ☐ **No**	Day (optional), month, year D D M M Y Y Y Y
b.	Chemotherapy	☐ **Yes** ☐ **No**	Day (optional), month, year D D M M Y Y Y Y
c.	Radiotherapy	☐ **Yes** ☐ **No**	Day (optional), month, year D D M M Y Y Y Y
d.	Other Please specify:	☐ **Yes** ☐ **No**	Day (optional), month, year D D M M Y Y Y Y
e.	Treatment not started yet	☐ **Yes**	

Sample

Copyright property of the International Benchmarking Partnership (ICBP) and Module 4 collaborators. Usage must be authorised

ICBP
International Cancer
Benchmarking Partnership

11. Who is the consultant doctor who has taken responsibility for diagnosing and or/treating your cancer?

Name of consultant:
Hospital name:
Hospital department:

Please can you answer some more general questions about your health?
It will help us in interpreting your responses to this questionnaire to know about your general health and other health problems you may have had in the past.

12. Looking back to the 2 years before you were diagnosed with cancer, would you say your general health was (Please tick only **ONE** answer.)**:**

Sample

Very good	
Good	
Fair	
Poor	
Very poor	

13. Have you been treated before for any of the conditions below?
Please tick 'yes' or 'no' for each condition:

Heart disease	☐ **Yes**	☐ **No**
Stroke	☐ **Yes**	☐ **No**
Lung disease	☐ **Yes**	☐ **No**
Diabetes	☐ **Yes**	☐ **No**

Copyright property of the International Benchmarking Partnership (ICBP)
and Module 4 collaborators. Usage must be authorised

ICBP
International Cancer
Benchmarking Partnership

Finally, a little more information about you. The information you provide below will help us to analyse the results of the survey in more detail.

14. Which of these best describes your ethnic group? (please tick one box, as appropriate). If you are descended from more than one ethnic or racial group, please tick the group you consider you belong to, or tick 'any other ethnic group'.

	✓		✓		✓		✓
White		Chinese		Black - Caribbean		Black - African	
Black - other		Indian		Pakistani		Bangladeshi	
Any other ethnic group, please specify							

Sample

15. What is the main language spoken in your home? Please tick

	✓
English	
Other, please specify:	

16. What is the highest level of education you have achieved?
Please tick only **ONE** answer.

	✓
Finished school at or before the age of fifteen	
Completed GCSEs, O-levels or equivalent	
Completed A Levels or equivalent	
Completed further education but not a degree	
Completed a Bachelor's degree / Masters degree / PhD	
Other, please specify:	

Copyright property of the International Benchmarking Partnership (ICBP)
and Module 4 collaborators. Usage must be authorised

ICBP
International Cancer
Benchmarking Partnership

17. **Have you ever smoked cigarettes, including hand-rolled ones, pipes or cigars?**

☐ Yes ☐ No

18. **Are you a current smoker, smoking either cigarettes, including hand-rolled ones, pipes or cigars?**

☐ Yes ☐ No

19. **If you are a current smoker or have smoked in the past, how many cigarettes, including hand-rolled ones, pipes or cigars on average do you smoke/have you smoked per day?**

Number per day:	

Sample

Copyright property of the International Benchmarking Partnership (ICBP)
and Module 4 collaborators. Usage must be authorised

ICBP
International Cancer
Benchmarking Partnership

20. Further comments

Please add anything else that you would like to tell us about your cancer diagnosis or treatment.

Sample

Thank you very much for taking the time to complete this questionnaire.

Copyright property of the International Benchmarking Partnership (ICBP)
and Module 4 collaborators. Usage must be authorised

ICBP
International Cancer
Benchmarking Partnership

International Cancer Benchmarking Partnership Module 4

Primary Care Audit
Colorectal Cancer

Thank you very much for agreeing to fill in this questionnaire. As part of an international study examining differences in cancer survival, we are sending the questionnaire to health care providers of a sample of consented patients with cancer.

Our aim is to gain a better understanding of the process by which people have their cancer diagnosed – the symptoms they experience, and the pathway they follow from onset of symptoms to treatment of their cancer. This will help in identifying ways in which cancers can be diagnosed and treated quickly and effectively. Thank you once again for your time.

Please can you refer to your patient's notes in completing the questionnaire as this will help in obtaining accurate data on time points.

If you would prefer to return this questionnaire without the patient details, please tear off along the dotted line

ID-number: Jurisdiction-ID + Patient-ID:

Copyright property of the International Benchmarking Partnership (ICBP) and Module 4 collaborators. Usage must be authorised

Sample
For peer review only

ICBP
International Cancer Benchmarking Partnership

Sample

For peer review only

Patient information

ID-number: Jurisdiction-ID + Patient-ID:

Full name:

Address:

Postcode:

Date of birth: D D M M Y Y Y Y

Copyright property of the International Benchmarking Partnership (ICBP)
and Module 4 collaborators. Usage must be authorised

ICBP
International Cancer
Benchmarking Partnership

1. Duration of symptoms

Please estimate how long your patient had symptom(s), attributable to colorectal cancer, before attending your practice (or other health service).

We appreciate that identifying a 'date of first symptom' is not always straightforward – particularly when there are multiple and/or chronic symptoms. Nevertheless, we hope you can provide a 'best estimate'.

**Estimate of symptom duration** (please tick **one**):	✓	**What were the symptoms?** Please describe:
Less than 1 week		
1 to 4 weeks		
5 to 7 weeks		
2-5 months		
6-12 months		
More than 12 months		
Not possible to estimate		
No symptoms (e.g. screen detected cancers)		

Sample
For peer review only

Copyright property of the International Benchmarking Partnership (ICBP) and Module 4 collaborators. Usage must be authorised

ICBP
International Cancer
Benchmarking Partnership

2. Pathway of presentation

2.1 Through what route did the patient first present? Please tick **ONE**.

Your patient first presented to primary care (either in-hours or out-of-hours)		Please can you provide your best approximation of the date of this primary care visit<br>D D M M Y Y Y Y
Your patient presented straight to A&E (with or without your involvement)		
Your patient first presented to primary care, but then at a later date presented to A&E as an emergency (with or without your involvement)		Please can you provide your best approximation of the date of this primary care visit<br>D D M M Y Y Y Y
Your patient's colorectal cancer was diagnosed through an organised screening programme (e.g. not as a result of investigation of symptoms)		
Other – please describe:		

Sample

For peer review only

Copyright property of the International Benchmarking Partnership (ICBP)
and Module 4 collaborators. Usage must be authorised

ICBP
International Cancer
Benchmarking Partnership

3. Date you ordered any tests/investigations in response to symptom(s).

We are interested in any kind of tests/investigations (e.g. imaging etc) that you may have ordered. Please only consider the tests/investigations that you ordered yourself. Please tick **ALL** that apply and put in the date that the test/investigation was ordered:

	✓	
Blood test		D D M M Y Y Y Y
Faecal occult blood test (FOBT)		D D M M Y Y Y Y
Colonoscopy		D D M M Y Y Y Y
Sigmoidoscopy		D D M M Y Y Y Y
Double contrast barium enema (DCBE)		D D M M Y Y Y Y
Digital Rectal Exam (DRE)		D D M M Y Y Y Y
Virtual colonoscopy (computerised tomographic colonography)		D D M M Y Y Y Y
Other (please specify):		D D M M Y Y Y Y

4. Date of referral to specialist medical services

At what date did you **first** refer the patient to hospital or another specialist transferring the responsibility for on-going investigation/treatment to other medical services?

D D M M Y Y Y Y

Sample
For peer review only

Copyright property of the International Benchmarking Partnership (ICBP) and Module 4 collaborators. Usage must be authorised

For peer review only - http://bmjopen.bmj.com/site/about/guidelines.xhtml

ICBP
International Cancer
Benchmarking Partnership

5. Nature of this referral

5.1 Do you know the date that the patient was seen for this referral?

☐ **Yes**, please provide the date: D D M M Y Y Y Y

☐ **No**

5.2 If you did make a referral to specialist services, which of the following best describes the nature/characteristics of this referral? Please tick **one**.

	✓
Emergency admission: a referral to A&E (or equivalent) for immediate admission	
An urgent referral for assessment of cancer symptoms/signs/test results (Note this will be within 2 weeks for England/Wales)	
A less urgent referral in which cancer is raised as a possibility (Note this will be greater than 2 weeks for England/Wales)	
A more general referral for investigation and assessment without cancer mentioned	
No referral was made	
Other – please describe	

5.3 Would you say this patient's diagnostic pathway was conducted predominantly in the public or private system? Please tick **one**.

	✓
Public healthcare system	
Private healthcare system	

Sample

For peer review only

Copyright property of the International Benchmarking Partnership (ICBP) and Module 4 collaborators. Usage must be authorised

ICBP
International Cancer
Benchmarking Partnership

6. Date of colorectal cancer diagnosis

This can be decided in different ways. Please provide whichever of the following dates you have to hand. Please tick **all** that apply.

	✓	
Date of histological confirmation [ideal]		D D M M Y Y Y Y
Date results of investigation (histological or other) confirming cancer received		D D M M Y Y Y Y
Date patient was told		D D M M Y Y Y Y
Date biopsy undertaken		D D M M Y Y Y Y
Date patient was first admitted to hospital because of the malignancy		D D M M Y Y Y Y
Other (please specify)		D D M M Y Y Y Y

Sample

For peer review only

Copyright property of the International Benchmarking Partnership (ICBP) and Module 4 collaborators. Usage must be authorised

ICBP
International Cancer
Benchmarking Partnership

7. Additional information

Finally, we are interested to know what other conditions your patient has, and the severity/impact of these conditions

Have you and/or any of your partners treated this patient (or has the patient been to hospital) for any of the following conditions? Please tick **all** that apply:

Cardiovascular disease	☐ Yes ☐ No
Stroke	☐ Yes ☐ No
Lung disease	☐ Yes ☐ No
Diabetes	☐ Yes ☐ No

Are there any other comments you would like to make about this patient?

Sample

Name (and title):

Signature:

Date:

Thank you very much for taking the time to complete this questionnaire.

Copyright property of the International Benchmarking Partnership (ICBP)
and Module 4 collaborators. Usage must be authorised

For peer review only - http://bmjopen.bmj.com/site/about/guidelines.xhtml

For peer review only

ICBP
International Cancer Benchmarking Partnership

International Cancer Benchmarking Partnership Module 4

Specialist Care Audit
Colorectal Cancer

Thank you very much for agreeing to fill in this questionnaire – it should take about 10 minutes to complete. As part of an international study examining differences in cancer survival, we are sending the questionnaire to health care providers of a sample of patients with cancer.

Our aim is to gain a better understanding of the process by which people have their cancer diagnosed – the symptoms they experience, and the pathway they follow from onset of symptoms to treatment of their cancer. We hope you can help us with information on this patient's cancer journey once they were referred to specialist cancer services. This will help in identifying ways in which cancers can be diagnosed and treated quickly and effectively.

Thank you once again for your time

Please can you refer to your patient's notes in completing the questionnaire, as this will help in obtaining accurate data on time points.

If you would prefer to return this questionnaire without the patient details, please tear off along the dotted line.

Your patient ______________________________

is participating in the study.

Copyright property of the International Benchmarking Partnership (ICBP) and Module 4 collaborators. Usage must be authorised

Sample
For peer review only

ICBP
International Cancer
Benchmarking Partnership

Sample

For peer review only

Patient information

ID-number: Jurisdiction-ID + Patient-ID:

Full name:

Address:

Postcode:

Date of birth: D D M M Y Y Y Y

Copyright property of the International Benchmarking Partnership (ICBP)
and Module 4 collaborators. Usage must be authorised

ICBP
International Cancer
Benchmarking Partnership

1. Date patient first attended hospital/specialist services related to their cancer diagnosis. We appreciate this date can at times be difficult to identify, particularly when there have been multiple visits in the lead up to a definitive diagnosis. Put another way, it's the date that the hospital/specialist service assumed responsibility for on-going investigation/treatment for your patient.

Day (optional), month, year

D	D	M	M	Y	Y	Y	Y

2. How was the patient referred to the hospital/specialist services related to their cancer diagnosis? Please tick.

Was it through a:

	✓		✓
GP referral		Screening	
Referral from general surgery clinic		Medical specialist/ Consultant referral	
Other referral – please specify:			

3. Where did this first contact/appointment happen? Please tick.
Which of the following best describes where this first contact/appointment took place?

	✓		✓
Emergency department ('A&E')		Medical outpatient department, please specify which department	
Oncology general outpatient department		Surgical outpatient department, please specify which department	
Other – please specify:			

Copyright property of the International Benchmarking Partnership (ICBP) and Module 4 collaborators. Usage must be authorised

Sample

For peer review only

ICBP
International Cancer
Benchmarking Partnership

4. Date of diagnosis

This can be decided in different ways.
Please tick and complete as many of the following dates as possible.

	✓	
Date of histological confirmation (ideal)		Day (optional), month, year<br>D D M M Y Y Y Y
Date results of investigation confirming cancer received		Day (optional), month, year<br>D D M M Y Y Y Y
Date patient was told		Day (optional), month, year<br>D D M M Y Y Y Y
Date of biopsy		Day (optional), month, year<br>D D M M Y Y Y Y
Date patient was first admitted to hospital because of the malignancy		Day (optional), month, year<br>D D M M Y Y Y Y
Date of MDT confirmation of diagnosis		Day (optional), month, year<br>D D M M Y Y Y Y
Other (please specify):		Day (optional), month, year<br>D D M M Y Y Y Y

Sample

For peer review only

Copyright property of the International Benchmarking Partnership (ICBP)
and Module 4 collaborators. Usage must be authorised

ICBP
International Cancer
Benchmarking Partnership

5. Date treatment for the cancer commenced

Based on your records, when would you say that any treatment specifically targeting the patient's cancer started?

Day (optional), month, year

D	D	M	M	Y	Y	Y	Y

6. Additional information

Please can you provide further information on the patient's cancer:

**TNM**, please tick as appropriate:		**Duke's**, please tick as appropriate:	
0		A	
I		B	
IIA		C	
IIB		D	
IIC			
IIIA			
IIIB			
IIIC			
IV			
Not able to stage			

Sample

Copyright property of the International Benchmarking Partnership (ICBP)
and Module 4 collaborators. Usage must be authorised

For peer review only - http://bmjopen.bmj.com/site/about/guidelines.xhtml

ICBP
International Cancer
Benchmarking Partnership

6.1 Histological subtype:

	✓
Adenocarcinoma	
Mucinous (colloid) adenocarcinoma	
Signet-ring cell carcinoma	
Other (please specify):	

Sample

For peer review only

Copyright property of the International Benchmarking Partnership (ICBP)
and Module 4 collaborators. Usage must be authorised

ICBP
International Cancer
Benchmarking Partnership

Further comments

Sample
For peer review only

Copyright property of the International Benchmarking Partnership (ICBP)
and Module 4 collaborators. Usage must be authorised

ICBP
International Cancer
Benchmarking Partnership

Name (and title): ______

Signature: ______

Date: ______

Sample

Are you a ... (please tick below): ✓

Surgeon	
Medical Oncologist	
Clinical Oncologist	
Clinical Nurse Specialist	
Other (please specify):	

Thank you very much for taking the time to complete this questionnaire.

Copyright property of the International Benchmarking Partnership (ICBP)
and Module 4 collaborators. Usage must be authorised

For peer review only

Supplementary File 5 – Ethical approvals, recruitment practices, ICBP M4 working group and ARG

Section 1 – Ethical and other approvals obtained in each Module 4 participating jurisdiction

	Date of Ethics Approval	Approvals obtained	Reference
**Victoria**	4 September 2012	Cancer Council Victoria Human Research Ethics Committee	HREC 1125
**Manitoba**	7 March 2013<br><br>15 April 2013	Health Research Ethics Board, University of Manitoba<br><br>Research Resource Ethics Committee, CancerCare Manitoba	HS15227 (H2012:105)<br><br>RRIC#28-2012
**Ontario**	7 November 2013<br>28 January 2014	University of Toronto Research Ethics Board	27881
**Denmark**	6 August 2013<br><br>19 June 2013	The Danish Data Protection Agency<br><br>According to Danish law and the Central Denmark Region Committees on Health Research Ethics, approval by the National Committee on Health Research Ethics was not required as no biomedical intervention was performed.	2013-41-2030<br><br>1-10-72-20-13
**Sweden**	23 October 2013	Ethics Review Board, Uppsala	2013/306
**Norway**	04 April 2013	Regional committees for medical and health research ethics	2013/136/REK nord
**Wales**	16 November 2012	NRES Committee East Midlands – Derby 2, local R&D for each health board	11/EM/0420
**Scotland**	16 November 2012	NRES Committee East Midlands – Derby 2,<br>R&D for each health board,<br>Privacy Advisory Committee,<br>CHI Advisory Group	11/EM/0420
**N Ireland**	1 June 2012	ORECNI Ethical approval, local governance for each health Trust	12/NI/0053
**England**	16 November 2012	NRES Committee East Midlands – Derby 2<br>R&D for each Clinical Research Network	11/EM/0420

For peer review only

Section 2 – Local recruitment practice in each Module 4 participating jurisdiction

	Recruitment practice variation
**Victoria**	The relevant healthcare professional confirmed eligibility prior to questionnaire mail-out to patients. Additional patients were recruited (above the required 200 symptomatic CRC patients) to meet the needs of a local study.
**Manitoba**	The data from cancer treatment specialists was not available.
**Ontario**	Additional patients were recruited (above the required 200 symptomatic CRC patients) to meet the needs of a local study.
**Denmark**	The cancer treatment specialist data were completed using clinical databases instead of through a survey.
**Sweden**	Only patients answered the survey – no primary care or cancer treatment specialist data available.
**Norway**	Some patients received and completed their surveys up to 9 months post diagnosis; their data were included (although flagged for subsequent analysis of any resulting sampling bias).
**Wales**	No variation.
**Scotland**	No variation.
**N Ireland**	The cancer treatment specialist data were collected directly from registries instead of through a survey. Some screen-detected cancer patients were excluded in the identification process.
**England**	No variation.

For peer review only

For peer review only - http://bmjopen.bmj.com/site/about/guidelines.xhtml

Section 3 – ICBP Module 4 Working Group

Alina Zalounina Falborg, Research Unit for General Practice, Department of Public Health, Aarhus University, Bartholins Allé 2, 8000 Aarhus C, Denmark

Andriana Barisic, Department of Prevention and Cancer Control, Cancer Care Ontario, 620 University Avenue, Toronto, Ontario, M5G 2L7, Canada

Anna Gavin, Northern Ireland Cancer Registry, Centre for Public Health, Queen's University Belfast, Mulhouse Building, Mulhouse Road, Belfast, BT12 6DP, United Kingdom

Anne Kari Knudsen, European Palliative Care Research Centre (PRC), Department of Oncology, Oslo University Hospital and Institute of Clinical Medicine, University of Oslo, Oslo, N-0424 Oslo, Norway

Breann Hawryluk, Department of Patient Navigation, Cancer Care Manitoba, 675 McDermot Street, Winnipeg, Manitoba, MB R3E 0V9, Canada

Chantelle Anandan, Centre for Population Health Sciences, University of Edinburgh, Doorway 1, Medical Quad Teviot Place, Edinburgh, EH8 9DX, United Kingdom

Conan Donnelly, Centre for Public Health, Queen's University Belfast, Mulhouse Building, Mulhouse Road, Belfast, BT12 6DP, United Kingdom

David H Brewster, Scottish Cancer Registry, Information Services Division, NHS National Services Scotland, Gyle Square, 1 South Gyle Crescent, Edinburgh, EH12 9EB, United Kingdom; Centre for Population Health Sciences, University of Edinburgh, Teviot Place, Edinburgh, EH8 9DX, United Kingdom

David Weller, Centre for Population Health Sciences, University of Edinburgh, Doorway 1, Medical Quad Teviot Place, Edinburgh, EH8 9DX, United Kingdom

Donna Turner, Population Oncology, Cancer Care Manitoba, 675 McDermot Street, Winnipeg, Manitoba, MB R3E 0V9, Canada

Elizabeth Harland, Department of Epidemiology and Cancer Registry, CancerCare Manitoba, 675 McDermot Street, Winnipeg, MB R3E 0V9, Manitoba

Eva Grunfeld, Knowledge Translation Research Network Health Services Research Program, Ontario Institute for Cancer Research; Professor and Vice Chair Research Department of Family and Community Medicine, University of Toronto, 500 University Avenue, Toronto, Ontario, M5G 1V7, Canada

Evangelia Ourania Fourkala, Gynaecological Cancer Research Centre, Women's Cancer, Institute for Women's Health, University College London, London, WC1E 6BT, United Kingdom

Henry Jensen, Research Unit for General Practice, Department of Public Health, Aarhus University, Bartholins Allé 2, 8000 Aarhus C, Denmark

For peer review only

For peer review only - http://bmjopen.bmj.com/site/about/guidelines.xhtml

Irene Reguilon, International Cancer Benchmarking Partnership, Cancer Research UK, London, EC1V 4AD, United Kingdom

Jackie Boylan, Centre for Public Health, Queen's University Belfast, Mulhouse Building, Mulhouse Road, Belfast, BT12 6DP, United Kingdom

Jacqueline Kelly, Northern Ireland Cancer Registry, Centre for Public Health, Queen's University Belfast, Mulhouse Building, Mulhouse Road, Belfast, BT12 6DP, United Kingdom

Jatinderpal Kalsi, Gynaecological Cancer Research Centre, Women's Cancer, Institute for Women's Health, University College London, London, WC1E 6BT, United Kingdom

John Butler, The Royal Marsden, Fulham Road, London, SW3 6JJ, United Kingdom

Kerry Moore, Centre for Public Health, Queen's University Belfast, Mulhouse Building, Mulhouse Road, Belfast, BT12 6DP, United Kingdom

Maria Rejmyr Davis, Southern Sweden Regional Cancer Center, Medicon Village, Scheelevägen 8, building 404, 223 81 Lund, Sweden

Martin Malmberg, Department of Oncology, Lund University Hospital, SE-221 85 Lund, Sweden

Mats Lambe, Regional Cancer Center Uppsala and Department of Medical Epidemiology and Biostatics, Karolinska Institutet, SE-171 77 Stockholm, Sweden

Oliver Bucher, Department of Epidemiology and Cancer Registry, CancerCare Manitoba, 675 McDermot Street, Winnipeg, MB R3E 0V9, Manitoba

Peter Vedsted, Research Unit for General Practice, Department of Public Health, Aarhus University, Bartholins Allé 2, 8000 Aarhus C, Denmark

Rebecca-Jane Law, North Wales Centre for Primary Care Research, Bangor University, Cambrian House 2, Wrexham Technology Park, Wrexham, LL13 7YP, United Kingdom

Rebecca Bergin, Centre for Behavioural Research in Cancer, 615 St Kilda Rd, Melbourne, Victoria, 3004, Australia; Department of General Practice, University of Melbourne, 200 Berkeley St, Victoria, 3053, Australia

Richard D Neal, North Wales Centre for Primary Care Research, Bangor University, Cambrian House 2, Wrexham Technology Park, Wrexham, LL13 7YP, United Kingdom; Academic Unit of Primary Care, Leeds Institute of Health Sciences, University of Leeds, Leeds, LS2 9NL, United Kingdom

Samantha Harrison, Early Diagnosis and International Cancer Benchmarking Partnership, Policy and Information, Cancer Research UK, London, EC1V 4AD, United Kingdom

For peer review only - http://bmjopen.bmj.com/site/about/guidelines.xhtml

Sigrun Saur Almberg, Department of Cancer Research and Molecular Medicine, Faculty of Medicine, Norwegian University of Science and Technology (NTNU), N-7491 Trondheim, Norway

Therese Kearney, Northern Ireland Cancer Registry, Centre for Public Health, Queen's University Belfast, Mulhouse Building, Mulhouse Road, Belfast, BT12 6DP, United Kingdom

Victoria Cairnduff, Northern Ireland Cancer Registry, Centre for Public Health, Queen's University Belfast, Mulhouse Building, Mulhouse Road, Belfast, BT12 6DP, United Kingdom

Victoria Hammersley, Centre for Population Health Sciences, University of Edinburgh, Doorway 1, Medical Quad Teviot Place, Edinburgh, EH8 9DX, United Kingdom

Victoria White, Centre for Behavioural Research in Cancer, Cancer Council Victoria, 615 St Kilda Road, Melbourne, Victoria, 3004, Australia; School of Psychology Deakin University, Geelong, Victoria, 3217, Australia

Usha Menon, Gynaecological Cancer Research Centre, Women's Cancer, Institute for Women's Health, University College London, London, WC1E 6BT, United Kingdom

Yulan Lin, European Palliative Care Research Centre (PRC), Department of Oncology, Oslo University Hospital and Institute of Clinical Medicine, University of Oslo, Oslo, N-0424 Oslo, Norway

For peer review only

Section 4 – ICBP Module 4 Academic Reference Group:

Prof Jan Willem Coebergh, Professor of Cancer Surveillance, Department of Public Health, Erasmus Universiteit Rotterdam, Rotterdam, the Netherlands

Jon Emery, Professor of Primary Care Cancer Research, University of Melbourne and Clinical Professor of General Practice, University of Western Australia, Australia

Dr Stefan Bergström, Senior consultant oncologist, Department of Oncology, Gävle, Sweden

Dr Monique E van Leerdam, Erasmus MC University Medical Centre, the Netherlands

Prof Marie-Louise Essink-Bot, Academic Medical Centre, Amsterdam University, the Netherlands

Prof Una MacLeod, Senior Lecturer in General Practice and Primary Care, Hull-York Medical School, United Kingdom

For peer review only

For peer review only - http://bmjopen.bmj.com/site/about/guidelines.xhtml

Supplementary File 6 – graphs of regression analysis for symptomatic patients (based on Table 6). The difference in the length of jurisdiction's intervals are shown compared to the reference Wales (days).

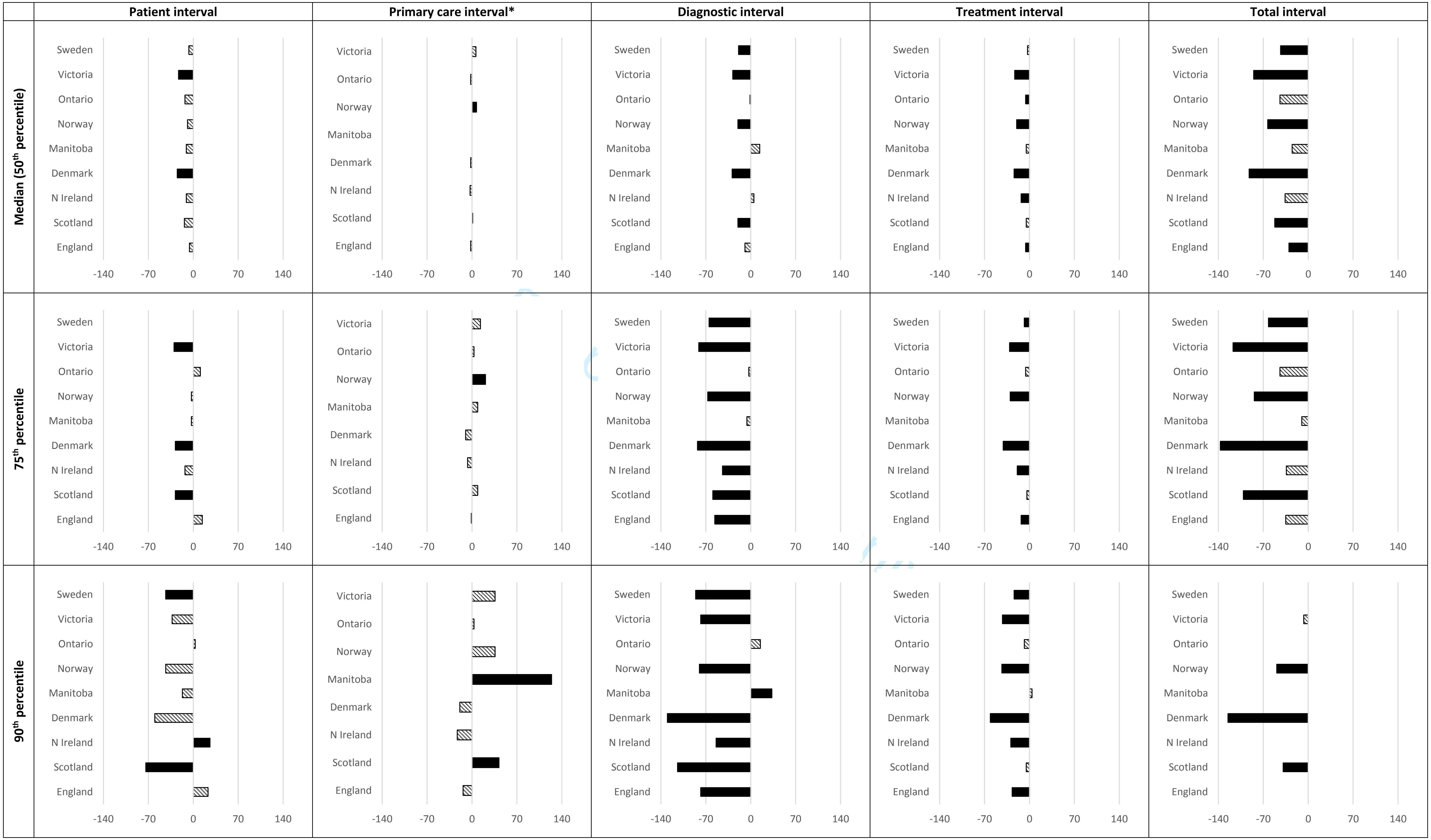

* Sweden did not provide any data for the primary care interval, and so has not been included in these graphs.

Differences in interval lengths (in days) are shown for the median, 75th and 90th percentiles compared to the reference used for the regression analyses, Wales. Wales is represented by the axis, with jurisdictions with shorter intervals shown to the left of the axis, and jurisdictions with longer intervals shown to the right of the axis for each graph. Statistically significant results are shown in solid bars, whilst non-significant results are shown with a pattern fill.

BMJ Open

Diagnostic routes and time intervals for colorectal cancer patients in ten international jurisdictions; findings from a cross-sectional study from the International Cancer Benchmarking Partnership (ICBP)

Journal:	*BMJ Open*
Manuscript ID	bmjopen-2018-023870.R1
Article Type:	Research
Date Submitted by the Author:	23-Jul-2018
Complete List of Authors:	Weller, David; University of Edinburgh, Centre for Population Health Sciences<br>Menon, Usha; University College London,<br>Zalounina Falborg, Alina; Research Unit for General Practice, Department of Public Health, Aarhus University<br>Jensen, Henry; Research Unit for General Practice, Department of Public Health, Aarhus University<br>Barisic, Andriana; Cancer Care Ontario, Renal Network<br>Knudsen, Anne Kari; Oslo University Hospital , European Palliative Care Research Centre (PRC), Department of Oncology<br>Bergin, Rebecca; Cancer Council Victoria, Centre for Behavioral Research in Cancer; University of Melbourne, Department of General Practice<br>Brewster, David; University of Edinburgh, Centre for Population Health Sciences; NHS National Services Scotland, Scottish Cancer Registry, Information Services Division<br>Cairnduff, Victoria; Northern Ireland Cancer Registry<br>Gavin, Anna; Queen's University Belfast, Northern Ireland Cancer Registry<br>Grunfeld, Eva; University of Toronto, Department of Family and Community Medicine<br>Harland, Elizabeth; CancerCare Manitoba, Department of Epidemiology and Cancer Registry<br>Lambe, Mats; Karolinska Institutet<br>Law, Rebecca-Jane; Bangor University, North Wales Centre for Primary Care Research<br>Lin, Yulan; Oslo University Hospital , European Palliative Care Research Centre (PRC), Department of Oncology<br>Malmberg, Martin; Skanes universitetssjukhus Lund, Department of Oncology<br>Turner, Donna<br>Neal, RD; Bangor University, North Wales Centre for Primary Care Research; University of Leeds, Academic Unit of Primary Care<br>White, Victoria; Cancer Council Victoria, Centre for Behavioral Research in Cancer; Deakin University School of Psychology<br>Harrison, Samantha; Cancer Research UK, Policy and Information<br>Reguilon, Irene; Cancer Research UK, Policy and Information<br>Working Group, ICBP; Cancer Research UK, Policy and Information<br>Vedsted, Peter; Research Unit for General Practice, Department of Public Health, Aarhus University

**Primary Subject Heading**:	Epidemiology
Secondary Subject Heading:	General practice / Family practice, Health policy, Health services research, Oncology
Keywords:	Health policy < HEALTH SERVICES ADMINISTRATION & MANAGEMENT, Organisation of health services < HEALTH SERVICES ADMINISTRATION & MANAGEMENT, International health services < HEALTH SERVICES ADMINISTRATION & MANAGEMENT, Gastrointestinal tumours < ONCOLOGY, Epidemiology < ONCOLOGY

SCHOLARONE™
Manuscripts

For peer review only

Diagnostic routes and time intervals for colorectal cancer patients in ten international jurisdictions; findings from a cross-sectional study from the International Cancer Benchmarking Partnership (ICBP).

Corresponding author:
David Weller
University of Edinburgh, Medical Quad, Teviot Row, Edinburgh EH8 9DX
david.weller@ed.ac.uk
+44 131 6509518

1. David Weller*, Centre for Population Health Sciences, University of Edinburgh, Edinburgh, United Kingdom
2. Usha Menon*, Institute for Women's Health, University College London, London, United Kingdom
3. Alina Zalounina Falborg, Research Unit for General Practice, Aarhus University, Aarhus, Denmark
4. Henry Jensen, Research Unit for General Practice, Aarhus University, Aarhus, Denmark
5. Andriana Barisic, Ontario Renal Network, Cancer Care Ontario, Toronto, Canada
6. Anne Kari Knudsen, European Palliative Care Research Centre (PRC), Department of Oncology, Oslo University Hospital and Institute of Clinical Medicine, University of Oslo, Oslo, Norway
7. Rebecca J Bergin, Centre for Behavioural Research in Cancer, Cancer Council Victoria, Victoria, Australia; Department of General Practice, University of Melbourne, Victoria, Australia
8. David H Brewster, Scottish Cancer Registry, Information Services Division, NHS National Services Scotland, Edinburgh, United Kingdom; Centre for Population Health Sciences, University of Edinburgh, Edinburgh, United Kingdom
9. Victoria Cairnduff, Northern Ireland Cancer Registry, Queen's University Belfast, Belfast, United Kingdom
10. Anna T Gavin, Northern Ireland Cancer Registry, Queen's University Belfast, Belfast, United Kingdom
11. Eva Grunfeld, Health Services Research Program, Ontario Institute for Cancer Research, Toronto, Canada
12. Elizabeth Harland, Department of Epidemiology and Cancer Registry, CancerCare Manitoba, Winnipeg, Canada
13. Mats Lambe, Regional Cancer Center Uppsala and Department of Medical Epidemiology and Biostatics, Karolinska Institutet, Stockholm, Sweden
14. Rebecca-Jane Law, North Wales Centre for Primary Care Research, Bangor University, Wrexham, United Kingdom
15. Yulan Lin, European Palliative Care Research Centre (PRC), Department of Oncology, Oslo University Hospital and Institute of Clinical Medicine, University of Oslo, Oslo, Norway
16. Martin Malmberg, Department of Oncology, Lund University Hospital, Lund, Sweden
17. Donna Turner, Population Oncology, Cancer Care Manitoba, Winnipeg, Canada
18. Richard D Neal, North Wales Centre for Primary Care Research, Bangor University, United Kingdom; Academic Unit of Primary Care, University of Leeds, Leeds, United Kingdom
19. Victoria White, Centre for Behavioral Research in Cancer, Cancer Council Victoria, Victoria, Australia; School of Psychology Deakin University, Victoria, Australia
20. Samantha Harrison, Policy and Information, Cancer Research UK, London, United Kingdom
21. Irene Reguilon, Policy and Information, Cancer Research UK, London, United Kingdom
22. ICBP Module 4 Working Group
23. Peter Vedsted*, Research Unit for General Practice, Aarhus University, Aarhus, Denmark

* Equal contribution

Word count: 4,319

For peer review only - http://bmjopen.bmj.com/site/about/guidelines.xhtml

Keywords: colorectal cancer, routes to diagnosis, time intervals, international health systems, symptomatic presentation

For peer review only

For peer review only - http://bmjopen.bmj.com/site/about/guidelines.xhtml

ABSTRACT

Objective: International differences in colorectal cancer (CRC) survival and stage at diagnosis have been reported previously. They may be linked to differences in time intervals and routes to diagnosis. The International Cancer Benchmarking Partnership Module 4 (ICBP M4) reports the first international comparison of routes to diagnosis for CRC patients and the time intervals from symptom onset until the start of treatment. Data came from patients in ten jurisdictions across six countries (Canada, the UK, Norway, Sweden, Denmark and Australia).

Design: CRC patients were identified via cancer registries. Data on symptomatic and screened patients were collected; questionnaire data from patients' primary care physicians and specialists, as well as information from treatment records or databases, supplemented patient data from the questionnaires. Routes to diagnosis and the key time intervals were described, as were between-jurisdiction differences in time intervals, using quantile regression.

Participants: A total of 14,664 eligible CRC patients diagnosed between 2013 and 2015 were identified, of which 2,866 were included in the analyses.

Primary and secondary outcome measures: Interval lengths in days (primary), reported patient symptoms (secondary).

Results: The main route to diagnosis for patients was symptomatic presentation and the most commonly reported symptom was 'bleeding/blood in stool'. The median intervals between jurisdictions ranged from: 21 to 49 days (patient); 0 to 12 days (primary care); 27 to 76 days (diagnostic); and 77 to 168 days (total, from first symptom to treatment start). Including screen-detected cases did not significantly alter the overall results.

Conclusion: ICBP M4 demonstrates important differences in time intervals between ten jurisdictions internationally. The differences may justify efforts to reduce intervals in some jurisdictions.

Strengths and limitations of this study

- This is the first international study of this scale to use standarised survey methods to systematically examine key intervals from patients first noticing symptoms or bodily changes until the start of treatment for their colorectal cancer
- Questionnaire data were enriched and validated with registry data (cancer registry and screening programmes) and data rules were applied consistentlyto ensure validity
- As with all questionnaire based studies, there may be some response differences due to participant interpretation, cohort characteristics and sampling strategy, but we did not find obvious differences between study participants which could bias our results.
- While our analyses adjusted for age, gender and comorbidity, we were unable to adjust for ethnicity and education due to different classification systems in participating countries
- Understanding variations in diagnostic and treatment intervals for colorectal cancer patients may, in jurisdictions with longer intervals, signal the need for improvements in service configuration and patient pathways.

For peer review only - http://bmjopen.bmj.com/site/about/guidelines.xhtml

For peer review only

BACKGROUND

Colorectal cancer (CRC) is a leading cause of morbidity and mortality and places a major burden on health systems; worldwide 1.36 million new cases are diagnosed every year.[1] CRC is the second most common cause of death due to cancer in Europe after female breast cancer, accounting for more than 200,000 deaths per year.[2] Prognosis strongly depends on stage at diagnosis, and the disease can mostly be cured if diagnosed at an early stage. Survival has increased over the last several years in Europe.[3] However, there remains substantial international variation in both 1- and 5-year survival, with countries such as the United Kingdom and Denmark having significantly poorer survival than other countries such as Sweden, Canada and Australia (Figure 1).[4] Some of the variation derives from differences in stage at diagnosis which in turn is a result of the pathway to diagnosis and treatment.[5] Therefore, it is crucial to investigate international differences in this pathway for CRC.

FIGURE 1

The International Cancer Benchmarking Partnership (ICBP) aims to not only quantify survival differences in comparable countries but to explore factors which may impact on observed differences.[6] Module 4 (ICBP M4) focusses on the routes to diagnosis and length of diagnostic and treatment intervals as a means of understanding differences in cancer prognosis between countries. This may help shape policy and practice interventions in participating jurisdictions. [6]

Diagnosis of CRC can be difficult; the symptoms are often vague (e.g. fatigue and non-specific abdominal pain), and this poses a significant diagnostic challenge for primary care, where most patients with CRC present.[7-12] There is growing evidence that prolonged diagnostic and treatment intervals are associated with poorer outcomes in CRC.[13,14] Access to investigations such as flexible sigmoidoscopy and colonoscopy is a further key issue; open access may expedite diagnosis and effect short diagnostic intervals.[12,15]

Many countries have implemented screening – typically faecal occult blood test (FOBT)-based – which can make a significant contribution to improved CRC outcomes.[16] However, currently, the large majority of CRC diagnoses are based on symptomatic presentation – for example, seeking help in primary care or attending emergency services.[7,17]

This study aims to systematically compare the diagnostic routes and time intervals from first noticing symptoms to start of treatment in CRC patients in ten healthcare systems with broadly similar access to high quality treatment and valid cancer registration.[6]

For peer review only - http://bmjopen.bmj.com/site/about/guidelines.xhtml

METHODS

The methods for ICBP M4 have been described.[18] In brief, we recruited patients through cancer registries in ten jurisdictions: Victoria (Australia); Manitoba and Ontario (Canada) Denmark; , Norway; Sweden; England, Scotland, Northern Ireland and Wales (United Kingdom). The target was to recruit 200 symptomatic recently diagnosed CRC patients per jurisdiction and to measure the patient, primary care, diagnostic, treatment and total intervals (Figure 2).

FIGURE 2

In defining these intervals we used principles articulated in the Aarhus Statement.[19] Data were collected from patients, their primary care physician (PCP) and their cancer treatment specialists (CTSs) as well as cancer registries. When calculating the route and time intervals we used predefined rules including a data 'hierarchy' around these information sources (Supplementary File 1). Based on a standardised protocol, teams within each jurisdiction established data collection processes with registries; survey logistics and data management were adapted to each local setting.

Data were transferred in anonymised format to the analysis team at Aarhus University – all data sources were combined into a single database.

Identification of study population

Eligible patients were consecutive patients aged 40 years or more with a first-diagnosis of CRC, ICD10 coded as C18.0-C18.9, C20.0 and C20.9.[20] Patients who had had another non-index cancer earlier were eligible, but those with synchronous different primary cancers were excluded.[18]

Each jurisdiction used a registry-based identification to enhance validity. We aimed to recruit patients 3 – 6 months after diagnosis; this avoided approaching patients too soon after diagnosis, while minimising recall bias from a long period post-diagnosis.

Recruitment was via cancer registries; either through 1) sending a letter to the relevant healthcare professional, requesting a pre-addressed envelope be forwarded to the patient on confirmation the person was aware of the diagnosis, or 2) the local research team or registry directly sending a letter to the patient.[18] Consent was required from all patients prior to surveys being sent to PCPS and CTS and for data transfer.

For peer review only - http://bmjopen.bmj.com/site/about/guidelines.xhtml

Data sources

Data from three questionnaires of eligible patients, their PCP and CTSs (Supplementary Files 2-4) were combined with information from participating cancer registries. Development, validation and implementation of these surveys is explained elsewhere.[18]

1. Survey data

Questionnaires were developed collaboratively with all jurisdictions. For consented patients, based on practice lists or the patient's response, a questionnaire was sent to the PCP with whom they were listed or who had been primarily involved in the diagnostic pathway. The patients and PCPs were asked about milestones, symptoms and route to cancer diagnosis. A questionnaire was sent to the CTSs who were first involved in the treatment. Jurisdictional differences in local recruitment processes are detailed in Supplementary File 5.

2. Registry data

To enhance complete and valid data on date of diagnosis, stage and screening status, data were collected through cancer registries wherever possible. Date of diagnosis was defined based on an established International Agency for Research on Cancer (IARC) hierarchy and stage was preferably given in tumour, node and metastasis (TNM) and Duke's.[21,22]

Data handling

Local teams entered data and questionnaire responses. The records were checked for obvious errors (eg. dates in future) and queries were discussed and resolved with local contacts, who checked repsones against original survey. All survey data underwent cleaning centrally (Aarhus University) to ensure that the same explicit rules were applied on the full dataset. Patients where age, date of diagnosis or date of consent were unknown were excluded.

As described the data rules allowed the combination of data from different sources in a standardised way that ensured reproducibility and transparency (Supplementary File 1). The rules, based on the Aarhus Statement,[19] employed a 'hierarchy' principle in terms of the order in which data sources (patient, PCP, CTS, registry) should take precedence where responses between sources differed, and included imputation rules based on the available data. The exact rule was guided by the measure in question – for example, patient interval was collected primarily from the patient questionnaire whereas primary care time-points were collected from the PCP questionnaire. All the measures were further validated using algorithms for outliers and out of range responses (e.g. negative time intervals).

For peer review only - http://bmjopen.bmj.com/site/about/guidelines.xhtml

Although the protocol mandated contacting patients within a 3-6-month time window after diagnosis, some local registries needed to extend this period, primarily due to delays in recording the cancer diagnosis.

Measures of routes to diagnosis

We defined routes to diagnosis for CRC using categories derived from the Aarhus Statement check-list – the following categories were used in the analysis:[19]

- Screening
- Symptomatic:
  - Visit PCP
  - Visit PCP and Accident and Emergency (A&E)
  - A&E
  - Investigation for another problem
- Other/unknown routes to diagnosis

Measures of time intervals

To ensure international comparison the time interval definition was adapted from the Aarhus Statement and included the following time-points:[19]

- First onset of symptoms: the time-point when first bodily change(s) and/or symptom(s) are noticed by the patient.
- First presentation to healthcare: the time-point at which it would be at least possible for the clinician seeing the patient to have started investigating.
- First referral to secondary care: the time-point at which the PCP refers the patient (and responsibility of the patient) to secondary/specialist care.
- Date of diagnosis: date the definite diagnosis was made, defined by the IARC hierarchy.[21]
- Date of start of treatment: the date where the patient started curative or palliative treatment or a decision not to treat.

The time intervals were calculated as the number of days between these time-points (Figure 2). For screen detected CRC, the patient and primary care interval were not applicable, with other intervals calculated using screening date as the first time-point. All time-points were validated manually and negative intervals were set to 0 days. Missing day was imputed based on specific rules to ensure that the direction of a possible misclassification bias was known (Supplementary File 1).

For peer review only - http://bmjopen.bmj.com/site/about/guidelines.xhtml

Establishing screening status

CRC patients were categorised using data rules as 'screen-detected', 'symptomatic' or 'other presentation'. In some jurisdictions it was possible to identify screen-detected cancers from registries; in others this categorisation depended on questionnaire responses. Due to differences in the understanding and registration of screening across jurisdictions, we specified symptom-based detection should include all patients who reported symptoms or A&E/primary care presentation, even if the patient had indicated 'screening' as the diagnostic route (unless their PCP or CTS specified a screening route). For UK countries the distinction between a screen-detected and non-screen-detected CRC was validated using registry data on screen-detected through public programs.

Covariates

Health status was measured using the self-reported general health item from the 36-Item Short Form Health Survey (SF36).[23] Comorbidity was assessed from the patient survey as presence of four diseases (stroke, diabetes, lung or heart diseases) and categorised into: 'none', 'medium' (one or two) or 'high' (three or four). Educational level was categorised as 'low' (vocational school or lower) and 'high' (university or higher). Symptoms reported were divided into two categories: 'a CRC specific symptom' or 'other symptoms'. This was based on a symptom coding done independently by two PCP-authors (DW and PV) with the aim of identifying symptoms where clinical suspicion could be raised.[24]

Statistical analysis

Quantile regression was used to estimate differences in intervals between all jurisdictions.[25] We compared the 50th, 75th and 90th percentiles. Wales was used as the reference jurisdiction as it had the lowest CRC survival according to the ICBP Module 1 cancer survival benchmark.[4] Quantile regression allows a comparison on the interval scale with optimal information on differences. Counting days, we used the 'qcount' procedure proposed by Miranda (2006).[26] Parameters were calculated with 1000 jittered samples. The differences (in days) in intervals between jurisdictions (using Wales as a reference) were calculated as marginal effects after quantile regression by setting the continuous covariate age to its mean value and the categorical covariates (gender and comorbidity) to their modes. Significance level was set to 0.05 or less, and 95% confidence intervals (95% CI) were calculated when appropriate. Statistical analyses were carried out using STATA v14 software.

Sensitivity and validity analyses

All analyses were undertaken using the 6 and 9 months cut-off criteria for allowable interval from diagnosis to questionnaire completion. To estimate the effect of using patient reported intervals only, a sensitivity

For peer review only - http://bmjopen.bmj.com/site/about/guidelines.xhtml

analysis based solely on patient data was performed. The effect of excluding patients for whom at least one time interval hadn't been reported was also investigated.

Kappa coefficient and overall agreement percentage assessed the agreement on routes to diagnosis (screening and symptomatic presentation) between the different data sources. Kappa coefficients were interpreted using Landis' and Koch's criteria:[27] 0.00 – 0.20 = slight, 0.21 – 0.40 = fair, 0.41 – 0.60 =moderate, 0.61 – 0.80 = substantial, above 0.80 = almost perfect.

Agreement between the different data sources was also assessed by Lin's concordance correlation coefficient (CCC).[28] The ICBP M4 definition of screening-status was validated against registry data on screening where available, and assessed by Kappa and overall agreement percentage.

Patient involvement

The research questions for this survey drew on an extensive literature elating diagnosis and treatment delays leading to negative patient experiences. While patient experience was not a primary outcome measure for this study, patients were given the opportunity to comment on their experience through questionnaire free-text response options (under separate analysis). Patients were involved in the piloting of study instruments to ascertain if recruitment and questionnaire content and dissemination strategies were appropriate, described elsewhere.[18] Each jurisdiction has committed to communicating the findings and local implications of this study to organisations representing their study participants.

RESULTS

Patient characteristics and participation

Of 14,664 eligible patients, 3,881 returned completed questionnaires (a 31% response rate, ranging from 19% in Norway to 69% in Denmark). Of these, 2,866 (95%) were included in the analyses after application of inclusion and exclusion criteria. The study flow with identification, exclusion and responses for each jurisdiction is seen in Table 1.

For peer review only - http://bmjopen.bmj.com/site/about/guidelines.xhtml

Table 1: Patient flow from identification to analyses for all ten jurisdictions and totally.

Jurisdiction	Wales		England		Scotland		N Ireland		Denmark		Manitoba		Norway		Sweden		Ontario		Victoria		Total	
Start date	04-10-2013		01-11-2013		01-12-2013		06-08-2013		28-10-2013		01-05-2013		01-09-2014		01-02-2014		30-04-2014		01-07-2013			
	n	(%)	n	(%)	n	(%)	n	(%)	n	(%)	n	(%)	n	(%)	n	(%)	n	(%)	n	(%)	n	(%)
**Eligible patients [a, b]**	**1274**	**(100%)**	**1314**	**(100%)**	**1852**	**(92.4%)**	**568**	**(45.0%)**	**490**	**(79.9%)**	**1288**	**(84.6%)**	**1860**	**(95.5%)**	**537**	**(85.8%)**	**5585**	**(71.8%)** [i]	**1170**	**(58.7%)**	**14664**	**(76.9%)**
**Packs sent to PCP [c]**	**1274**	**(100%)**	**1198**	**(91.2%)**	**1070**	**(57.8%)**															**3542**	**(79.8%)**
- Pack not forwarded by PCP	*211*	*(16.6%)*	*87*	*(7.3%)*	*103*	*(9.6%)*															401	(11.3%)
- Unsure if pack forwarded by PCP	*333*	*(26.1%)*	*362*	*(30.2%)*	*209*	*(19.5%)*															904	(25.5%)
- Pack forwarded by PCP	*730*	*(57.3%)*	*749*	*(62.5%)*	*758*	*(70.8%)*															2237	(63.2%)
**Patients contacted by PCP [c, d]**	**1063**	**(83.4%)**	**1111**	**(92.7%)**	**967**	**(90.4%)**															**3141**	**(88.7%)**
**Patients approached directly [c]**							**555**	**(97.7%)**	**490**	**(100%)**	**761**	**(59.1%)**	**1860**	**(100%)**	**537**	**(100%)**	**5099**	**(91.3%)**	**1049**	**(89.7%)**	**10351**	**(70.6%)**
- Patient died											*49*	*(3.2%)*					139	(1.8%)			188	(1.8%)
- Other							*13*	(1.0%)			*26*	(1.7%)					368	(4.7%)			407	(3.9%)
- No address											*11*	(0.7%)					309	(4.0%)			320	(3.1%)
**Patient responses (% of eligible patients) [c]**	**314**	**(24.6%)**	**285**	**(21.7%)**	**337**	**(18.2%)**	**283**	**(49.8%)**	**340**	**(69.4%)**	**274**	**(21.3%)**	**358**	**(19.2%)**	**319**	**(59.4%)**	**899**	**(16.1%)**	**472**	**(40.3%)**	**3881**	**(26.5%)**
**Patient responses (% of contacted) [e]**	**314**	**(29.5%)**	**285**	**(25.7%)**	**337**	**(34.9%)**	**283**	**(52.2%)**	**340**	**(69.4%)**	**274**	**(40.6%)**	**358**	**(19.2%)**	**319**	**(59.4%)**	**899**	**(21.0%)**	**472**	**(45.0%)**	**3881**	**(30.9%)**
- Did not fulfil eligibility criteria	7	(2.2%)	1	(0.4%)			1	(0.4%)			1	(0.4%)									*10*	*(0.3%)*
- Received after submission							20	(7.1%)											55	(11.7%)	*75*	*(2.1%)*
- Other	7	(2.2%)			57	(16.9%)	6	(2.1%)	16	(4.7%)	11	(4.0%)					45	(5.0%)	127	(26.9%)	*269*	*(7.6%)*
**Patient surveys forwarded for analyses [f]**	**300**	**(95.5%)**	**284**	**(99.6%)**	**280**	**(83.1%)**	**256**	**(90.5%)**	**324**	**(95.3%)**	**262**	**(95.6%)**	**358**	**(100%)**	**319**	**(100%)**	**854**	**(95.0%)**	**290**	**(61.4%)**	**3527**	**(90.9%)**
Excluded for analyses – total	*17*	*(5.7%)*	*10*	*(3.5%)*	*7*	*(2.5%)*	*3*	*(1.2%)*	*7*	*(2.2%)*	*4*	*(1.5%)*	*72*	*(20.1%)*	*8*	*(2.5%)*	*532*	*(62.3%)*	*1*	*(0.3%)*	*661*	*(18.7%)*
- Not sampled	*0*	*(0%)*	*0*	*(0%)*	*0*	*(0%)*	*0*	*(0%)*	*0*	*(0%)*	*0*	*(0%)*	*0*	*(0%)*	*0*	*(0%)*	*494*	*(57.8%)*	*0*	*(0%)*	*494*	*(14%)*
- Previous cancer	*0*	*(0%)*	*3*	*(1.1%)*	*0*	*(0%)*	*0*	*(0%)*	*0*	*(0%)*	*0*	*(0%)*	*0*	*(0%)*	*0*	*(0%)*	*1*	*(0.1%)*	*0*	*(0%)*	*4*	*(0.1%)*
- Unknown date of consent	*0*	*(0%)*	*0*	*(0%)*	*0*	*(0%)*	*0*	*(0%)*	*0*	*(0%)*	*0*	*(0%)*	*3*	*(0.8%)*	*6*	*(1.9%)*	*0*	*(0%)*	*0*	*(0%)*	*9*	*(0.3%)*
- Unknown date of diagnosis	*0*	*(0%)*	*0*	*(0%)*	*0*	*(0%)*	*0*	*(0%)*	*0*	*(0%)*	*1*	*(0.4%)*	*6*	*(1.7%)*	*0*	*(0%)*	*0*	*(0%)*	*0*	*(0%)*	*7*	*(0.2%)*
- Consent too late/too early	*17*	*(5.7%)*	*7*	*(2.5%)*	*7*	*(2.5%)*	*3*	*(1.2%)*	*0*	*(0%)*	*3*	*(1.1%)*	*60*	*(16.8%)*	*1*	*(0.3%)*	*37*	*(4.3%)*	*1*	*(0.3%)*	*136*	*(3.9%)*
- Other	*0*	*(0%)*	*0*	*(0%)*	*0*	*(0%)*	*0*	*(0%)*	*7*	*(2.2%)*	*0*	*(0%)*	*3*	*(0.8%)*	*1*	*(0.3%)*	*0*	*(0%)*	*0*	*(0%)*	*11*	*(0.3%)*
**Patient surveys analysed (% of forwarded surveys)**	**283**	**(94.3%)**	**274**	**(96.5%)**	**273**	**(97.5%)**	**253**	**(98.8%)**	**317**	**(97.8%)**	**258**	**(98.5%)**	**286**	**(79.9%)**	**311**	**(97.5%)**	**322**	**(89.4%)**	**289**	**(99.7%)**	**2866**	**(94.5%)** [k]
**PCP surveys analysed (% of analysed patients)**	**234**	**(82.7%)**	**225**	**(82.1%)**	**224**	**(82.1%)**	**213**	**(84.2%)**	**241**	**(76.0%)**	**148**	**(57.4%)**	**169**	**(59.1%)**	**n/a**	**n/a**	**121**	**(37.7%)**	**199**	**(68.9%)**	**1774**	**(69.5%)** [l]
**Specialist surveys analysed (% of analysed patients)**	**164**	**(58.0%)**	**156**	**(56.9%)**	**179**	**(65.6%)**	**n/a** [g]	**n/a**	**187** [g]	**(59%)**	**n/a** [h]	**n/a**	**64**	**(22.4%)**	**n/a** [h]	**n/a**	**89**	**(27.7%)**	**99**	**(34.3%)**	**938**	**(45.9%)** [m]

[a] Eligible according to protocol: i.e. woman, 40 years or more, alive, consented to participate within nine months of diagnosis, diagnosed with breast cancer (ICD-10: C50.0-C50.9), behaviour code ICD-O-3=3 and without prior history of cancer of the breast or synchronous primary breast cancer. [b] In some jurisdictions some 'eligible' patients had pre-opted out of being contacted and in a small number PCP information was not available. [c] Percentages of eligible patients. [d] Maximum of potentially contacted patients, i.e. sum of packs forwarded by PCP and packs unsure if forwarded by PCP. [e] Percentages of patients contacted by PCP (see note d) for Wales, England and Scotland or percentages of patients contacted directly by a registry excl. non-accessible patients (all other jurisdictions). [f] Percentages of patient responses. [g] Data obtained from registries instead: N Ireland from from the N Ireland Cancer Registry, supported by extracts from clinical datasets; Denmark from the Danish Colorectal Cancer Group (DCCG). [h] Data not collected in this jurisdiction. [i] initially 1458 additional cases were eligible but excluded for this study as urban sample size was reached. [j] Additionally 92 cases were ineligible based on registry-criteria exclusions and a further 108 excluded after approaching the treating doctor. [k] Denominator = total number of forwarded cases excl. patients not included in analytic sample in Ontario. [l] Denominator = total number of analysed cases excl. patients from Sweden. [m] Denominator = total number of analysed cases excl. patients from Sweden, Manitoba & N Ireland.

For peer review only

The characteristics of the included patients are detailed in Table 2. The patient questionnaire was completed at a median of 5 months following diagnosis. For tumour stage the proportion of missing stage was high in Norway due to lack of registry data.

Table 2. *The characteristics of the 2866 patients aged 40 or over with a first diagnosis of CRC included in the analyses (% if nothing else stated)*

	**Wales (N=283)**	**England (N=274)**	**Scotland (N=273)**	**N Ireland (N=253)**	**Denmark (N=317)**	**Manitoba (N=258)**	**Norway (N=286)**	**Sweden (N=311)**	**Ontario (N=322)**	**Victoria (N=289)**	**Total (N=2866)**
Median (range) interval for diagnosis to questionnaire completion in months	5 (3,9)	5 (1,9)	5 (3,9)	4 (2,6)	5 (0.5,8)	6 (3,9)	7 (0.03,9)	4 (0.5,7)	6 (1,9)	6 (3,9)	5 (0.03,9)
**Age** years											
Median (IQI)	71 (65,79)	71 (64,78)	70 (61,77)	67 (60,74)	71 (65,77)	69 (59,77)	70 (62,77)	72 (65,79)	69 (61,77)	66 (58,76)	70 (62,77)
**Age** years (Symptomatic patients)	(N=208)	(N=212)	(N=192)	(N=214)	(N=311)	(N=176)	(N=264)	(N=307)	(N=257)	(N=220)	(N=2361)
Median (IQI)	72 (64,81)	72 (64,80)	72 (61,80)	67 (59,76)	71 (65,77)	73 (60,81)	70 (62,77)	72 (65,79)	69 (61,77)	69 (57,77)	71 (62,78)
**Age** years (Screen-detected patients)	(N=73)	(N=58)	(N=78)	(N=35)	(N=0)	(N=81)	(N=18)	(N=0)	(N=55)	(N=62)	(N=460)
Median (IQI)	67 (65,73)	68 (65,74)	66 (62,72)	68 (63,70)	n/a	65 (57,70)	69 (61,74)	n/a	68 (63,72)	65 (60,69)	67 (63,72)
**Gender**											
Male	59.0	56.2	58.6	58.9	59.0	56.6	51.4	51.1	56.5	56.1	56.3
**Health State**											
Good	80.6	84.7	84.6	75.5	82.3	88.0	74.1	78.8	85.1	82.4	81.6
Fair	13.8	11.7	10.6	17.4	13.6	9.3	22.0	16.1	11.5	12.1	13.8
Poor	5.0	3.3	3.3	6.7	1.6	1.9	2.8	3.2	2.5	4.2	3.4
Missing	0.7	0.4	15	0.4	2.5	0.8	1.1	1.9	0.9	1.4	1.2
**Comorbidity** [1]											
No	52.7	58.8	57.1	55.3	50.2	63.2	61.9	58.5	53.7	61.9	57.2
Medium	44.2	38.7	41.8	43.5	46.7	34.9	35.0	35.1	31.4	35.6	38.6
High	2.5	2.6	0.7	1.2	1.9	1.6	3.2	4.5	1.9	1.7	2.2
Missing	0.7	0.0	0.4	0.0	1.3	0.4	0.0	1.9	13.0	0.7	2.0
**Education**											
Low	76.7	80.7	74.7	77.5	76.0	80.2	75.2	78.8	73.3	77.5	77.0
High	15.6	14.2	19.8	13.0	12.0	17.8	18.9	20.3	23.9	21.5	17.8
Missing	7.8	5.1	5.5	9.5	12.0	1.9	5.9	1.0	2.8	1.0	5.2
**Ethnicity**											
White	99.9	98.5	98.5	99.6	95.9	93.4	99.7	99.4	92.6	94.5	97.1
Asian	0.4	0.4	0.7	0.0	0.3	1.9	0.0	0.3	5.9	2.4	1.3
Black	0.0	0.0	0.4	0.0	0.0	0.4	0.0	0.0	0.6	0.0	0.1
Other	0.0	0.0	0.0	0.0	0.0	3.5	0.0	0.0	0.3	0.0	0.4
Missing	0.0	1.1	0.4	0.4	3.8	0.8	0.3	0.3	0.6	3.1	1.1
**Smoking**											
Currently	4.2	2.6	8.1	9.1	11.4	8.9	7.0	4.8	4.4	4.8	6.5
In the past	55.5	54.7	51.3	49.0	55.2	50.8	56.3	52.7	59.3	51.6	53.8
Never	39.9	41.2	40.7	39.5	31.6	39.9	36.4	42.4	35.4	42.6	38.8
Missing	0.4	1.5	0.0	2.4	1.9	0.4	0.3	0.0	0.9	1.0	0.9
**Tumour stage – TNM & Duke's**											
0	0.4	0.4	0.0	0.0	0.0	0.0	0.0	0.0	0.6	0.0	0.1

For peer review only

For peer review only - http://bmjopen.bmj.com/site/about/guidelines.xhtml

	Wales (N=283)	England (N=274)	Scotland (N=273)	N Ireland (N=253)	Denmark (N=317)	Manitoba (N=258)	Norway (N=286)	Sweden (N=311)	Ontario (N=322)	Victoria (N=289)	Total (N=2866)
I	19.4	20.8	18.7	18.2	20.5	17.1	3.9	16.7	27.0	26.0	19.0
II	25.4	29.6	32.6	29.6	36.0	28.3	4.9	26.1	32.0	35.6	28.1
III	40.3	30.7	35.9	37.6	24.6	39.5	7.0	31.5	28.0	29.1	30.1
IV	9.5	16.1	12.1	13.4	13.9	11.6	4.6	17.4	6.5	8.3	11.3
Missing	5.0	2.6	0.7	1.2	5.0	3.5	79.7	8.4	5.9	1.0	11.4
**Tumour stage – TNM & Duke’s** (Symptomatic patients)	(N=208)	(N=212)	(N=192)	(N=214)	(N=311)	(N=176)	(N=264)	(N=307)	(N=256)	(N=220)	(N=2360)
0	0.5	0.5	0.0	0.0	0.0	0.0	0.0	0.0	0.4	0.0	0.1
I	16.4	17.0	13.5	14.0	20.9	15.3	3.8	16.6	22.6	20.9	16.2
II	26.4	30.7	34.9	31.8	35.1	29.6	4.2	26.4	33.5	36.8	28.6
III	39.9	31.6	36.5	38.3	25.1	36.9	7.2	31.6	29.6	30.9	29.9
IV	11.5	17.0	14.1	14.5	13.8	13.1	4.9	17.3	8.2	10.0	12.4
Missing	5.3	3.3	1.0	1.4	5.1	5.1	79.9	8.1	5.8	1.4	12.8
**Tumour stage – TNM & Duke’s** (Screen-detected patients)	(N=73)	(N=58)	(N=78)	(N=35)	(N=0)	(N=81)	(N=18)	(N=0)	(N=55)	(N=62)	(N=460)
0	0.0	0.0	0.0	0.0	n/a	0.0	0.0	n/a	1.8	0.0	0.2
I	28.8	36.2	30.8	40.0	n/a	21.0	5.6	n/a	47.3	47	33.3
II	23.3	24.1	28.2	17.1	n/a	24.7	16.7	n/a	21.8	29.0	24.4
III	41.1	27.6	33.3	34.3	n/a	45.7	5.6	n/a	21.8	21.0	32.0
IV	2.7	12.1	7.7	8.6	n/a	8.6	0.0	n/a	0.0	3.2	5.9
Missing	4.1	0.0	0.0	0.0	n/a	0.0	72.2	n/a	7.3	0.0	4.4

[1] Comorbidity coded as no=none reported, medium=1-2 reported and high=3+ reported
Abbreviations: IQI=inter-quartile interval; n/a=not applicable

Routes to diagnosis

Routes to diagnosis were broadly similar, except for screening; of all patients, 16.1% had a screen detected CRC, ranging from 6.3% in Norway to 31.4% in Manitoba (Table 3). In Denmark and Sweden CRC-screening had not been implemented at the time of study and screening status in Norway was determined by information from local screening trials. The proportion of screen-detected cancers in Northern Ireland is lower as most were excluded in the recruitment process, with the local team actively including symptomatic patients in order to reach the target of 200 symptomatic patients. Overall (excluding Northern Ireland), most (82.2%) respondents presented with a symptomatic CRC. A high level of agreement was found between ICBP and registry data for screening status (Table 3). PCP data indicated that percentage of the symptomatic patients urgently referred with a suspicion of cancer was less than 50% in Ontario, Denmark, Manitoba, Norway (37.8%, 39.6%, 46.8%, 47.8%, correspondingly) and larger than 50% in Scotland, Northern Ireland, Victoria, England, Wales (51.5%, 57.1%, 57.6%, 67.6%, 69.3%, correspondingly).

For peer review only - http://bmjopen.bmj.com/site/about/guidelines.xhtml

Table 3. *The overall route (symptomatic or screened) for CRC in each jurisdiction (%) and place of initial presentation for symptomatic patients*

	Wales (N=283)	England (N=274)	Scotland (N=273)	N Ireland[1] (N=253)	Denmark (N=317)	Manitoba (N=258)	Norway[2] (N=286)	Sweden (N=311)	Ontario (N=322)	Victoria (N=289)	Total (N=2866)
**Symptomatic**	**73.5**	**77.3**	**70.3**	**84.6**	**98.1**	**68.2**	**92.3**	**99.0**	**79.8**	**76.1**	**82.4**
Visit PCP, Visit PCP and A&E[3]	*77.4*	*82.6*	*92.3*	*83.2*	*82.3*	*67.1*	*78.4*	*51.8*	*62.3*	*73.2*	*73.2*
A&E[3]	*7.2*	*3.8*	*0.5*	*6.5*	*3.2*	*9.1*	*4.6*	*11.7*	*8.2*	*8.6*	*6.7*
Investigation for another problem[3]	*6.7*	*4.7*	*4.8*	*3.3*	*9.3*	*9.7*	*9.9*	*32.9*	*11.3*	*7.3*	*11.1*
Other[3]	*8.7*	*9.0*	*2.4*	*7.0*	*5.1*	*14.2*	*7.2*	*3.6*	*18.3*	*10.9*	*8.9*
**Screening**	**25.8**	**21.2**	**28.6**	**13.8**	**0.0**	**31.4**	**6.3**	**0.0**	**17.1**	**21.5**	**16.1**
**Other**	**0.7**	**1.5**	**1.1**	**1.6**	**1.9**	**1.0**	**1.4**	**1.0**	**3.1**	**2.4**	**1.5**

Agreement between the ICBP M4 Presentation-rule (without using registry data) and registry information to define if a CRC case was screen detected:

Jurisdiction	Number of cases	Agreement on screening-status between ICBP4 and Registry %	Kappa	(95%CI)
Wales	277	92	0.78	(0.68-0.86)
England	259	95	0.84	(0.74-0.92)
Scotland	270	93	0.83	(0.75-0.90)
N Ireland	251	94	0.75	(0.61-0.87)

[1] In N Ireland the proportion of screen detected CRC cases was lower as these patients were primarily excluded from the eligible group
[2] The reporting of screening status in Norway was based on local screening trials.
[3] Percentage of Symptomatic-route

For peer review only

For peer review only - http://bmjopen.bmj.com/site/about/guidelines.xhtml

Symptom prompting concern

The proportion (%) of patients and PCPs reporting symptoms are shown in Table 4 (for Northern Ireland and Sweden, only patient data were collected). The most common symptom reported by PCP respondents was rectal bleeding (40% of respondents), followed by change in bowel habit. While every third patient indicated fatigue as a key symptom, it was rarely reported by PCPs.

Table 4. *The symptoms experienced by patients before presentation and the presenting symptoms seen by the PCP for the 2,361 patients aged 40 or over with a first diagnosis of CRC who had a symptom based diagnosis. All figures are %*

	Wales	England	Scotland	N Ireland	Denmark	Manitoba	Norway	Sweden	Ontario	Victoria	Total
**First symptom (reported by patient)**	(N=208)	(N=212)	(N=192)	(N=214)	(N=311)	(N=176)	(N=264)	(N=307)	(N=257)	(N=220)	(N=2361)
Bleeding/blood in stool	43.8	41.0	37.5	43.0	34.7	42.0	51.9	47.2	50.6	37.3	43.1
Bowel habit change	42.3	33.5	33.9	42.1	31.8	27.3	28.4	35.8	48.3	31.4	35.5
Fatigue	31.3	33.5	34.4	37.9	19.3	31.3	24.6	30.0	31.5	29.6	29.7
Abdominal pain	24.0	25.0	28.7	27.6	19.3	26.7	22.0	19.2	22.2	28.2	23.7
Weight loss	18.8	19.3	22.4	17.3	14.2	14.7	14.0	18.2	15.6	16.4	16.9
Other	40.4	35.9	37.0	42.5	27.3	34.7	31.1	40.1	18.7	30.5	33.4
No symptoms	6.7	3.8	9.9	4.7	9.7	14.8	7.6	9.5	5.8	8.2	8.0
Missing	1.4	1.9	2.6	1.9	10.3	3.4	3.0	1.0	1.2	0.9	3.0
**Number of symptoms per patient**											
Median (IQI)	2(1,3)	2(1,3)	2(1,3)	2(1,3)	1(1,2)	2(1,3)	2(1,2)	2(1,3)	2(1,3)	2(1,3)	2(1,3)
**Presenting symptom (reported by PCP)**	(N=140)	(N=151)	(N=132)	(N=0)	(N=212)	(N=77)	(N=134)	(N=0)	(N=82)	(N=118)	(N=1046)
Bleeding/blood in stool	37.1	33.8	33.3	n/a	29.1	26.0	29.1	n/a	32.9	33.9	31.9
Bowel habit change	33.6	27.8	21.2	n/a	26.9	16.9	17.9	n/a	19.5	21.2	24.1
Fatigue	4.3	1.3	3.8	n/a	7.6	5.2	4.5	n/a	9.8	8.5	5
Abdominal pain	20.7	15.2	17.4	n/a	18.9	16.9	12.7	n/a	15.9	26.3	18.1
Weight loss	8.6	6.6	8.3	n/a	7.6	1.3	3.0	n/a	3.7	6.8	6.2
Other	36.4	30.5	36.4	n/a	34.9	27.3	33.6	n/a	18.3	39.8	33.2
No symptoms	3.6	4.6	6.8	n/a	0.5	15.6	0.0	n/a	3.7	1.7	3.7
Missing	8.6	8.6	9.1	n/a	16.5	26.0	8.2	n/a	17.1	11.0	12.4
**Cancer-specificity of symptom presented**											
Cancer-specific symptom	67.9	66.2	56.1	n/a	57.6	46.8	53.0	n/a	48.8	63.6	58.6
Non-specific symptom	20.0	20.5	28.0	n/a	25.5	11.7	38.8	n/a	30.5	23.7	25.2
No symptoms /missing	12.1	13.3	15.9	n/a	17.0	41.6	8.2	n/a	20.7	12.7	16.2

Abbreviations: IQI=inter-quartile interval; n/a=not applicable

For peer review only - http://bmjopen.bmj.com/site/about/guidelines.xhtml

Time intervals

The median patient interval varied from 21 days (Denmark) to 49 days (Wales) (Table 5). Table 6 shows the adjusted patient median interval was 25 days shorter in Denmark than in Wales; none were longer compared to Wales (Supplementary File 6).

For peer review only

For peer review only - http://bmjopen.bmj.com/site/about/guidelines.xhtml

Table 5. *The time intervals (days) for each of the ten jurisdictions depicted as median, 75th and 90th percentiles. In Sweden no data on the primary care interval was available*

		Wales	England	Scotland	N Ireland	Denmark	Manitoba	Norway	Sweden	Ontario	Victoria
Patient Interval (Symptomatic patients)	Number	(n=195)	(n=199)	(n=175)	(n=199)	(n=292)	(n=134)	(n=240)	(n=266)	(n=230)	(n=199)
	Median	49	34	30	35	21	34	36	31	31	22
	75th percentile	92	118	73	88	62	92	92	92	96	63
	90th percentile	249	346	181	312	180	215	218	201	304	234
Primary Care interval (Symptomatic patients)	Number	(n=157)	(n=152)	(n=127)	(n=160)	(n=207)	(n=72)	(n=124)	(n=0)	(n=77)	(n=117)
	Median	3	2	4	0	1	4	12	n/a	1	9
	75th percentile	20	21	28	14	10	30	39	n/a	23	32
	90th percentile	78	54	93	54	51	210	82	n/a	70	128
Diagnostic interval (Symptomatic patients)	Number	(n=194)	(n=196)	(n=174)	(n=190)	(n=290)	(n=133)	(n=229)	(n=249)	(n=218)	(n=197)
	Median	60	48	38	64	27	76	37	36	54	28
	75th percentile	155	86	91	111	66	162	85	82	146	66
	90th percentile	284	201	164	238	129	365	222	196	312	200
Diagnostic interval (Screen-detected patients)	Number	(n=69)	(n=56)	(n=76)	(n=35)	(n=0)	(n=25)	(n=14)	(n=0)	(n=50)	(n=38)
	Median	35	25	36	0	n/a	66	22	n/a	3	40
	75th percentile	65	46	49	0	n/a	111	48	n/a	43	64
	90th percentile	99	70	76	0	n/a	206	84	n/a	120	122
Diagnostic interval (All patients)	Number	(n=263)	(n=252)	(n=250)	(n=225)	(n=290)	(n=158)	(n=243)	(n=249)	(n=268)	(n=235)
	Median	52	43	37	47	27	72	36	36	44	28
	75th percentile	120	76	72	101	66	139	85	82	128	64
	90th percentile	242	176	151	207	129	320	212	196	278	178
Treatment interval (Symptomatic patients)	Number	(n=197)	(n=206)	(n=185)	(n=208)	(n=306)	(n=161)	(n=258)	(n=281)	(n=248)	(n=209)
	Median	39	31	33	25	14	34	18	35	33	14
	75th percentile	59	47	56	40	19	59	29	52	54	29
	90th percentile	83	60	79	58	28	97	45	65	79	47
Treatment interval (Screen-detected patients)	Number	(n=72)	(n=58)	(n=78)	(n=34)	(n=0)	(n=79)	(n=17)	(n=0)	(n=52)	(n=60)
	Median	44	39	49	38	n/a	38	19	n/a	40	17
	75th percentile	68	46	71	52	n/a	61	27	n/a	54	35
	90th percentile	80	62	91	61	n/a	83	43	n/a	88	44
Treatment interval (All patients)	Number	(n=271)	(n=268)	(n=266)	(n=246)	(n=312)	(n=240)	(n=279)	(n=284)	(n=310)	(n=276)
	Median	41	34	37	27	14	35	18	36	34	15
	75th percentile	63	47	63	42	19	60	28	53	54	29
	90th percentile	80	61	87	59	27	88	43	65	82	44
Total interval (Symptomatic patients)	Number	(n=154)	(n=165)	(n=147)	(n=175)	(n=249)	(n=123)	(n=210)	(n=238)	(n=214)	(n=168)
	Median	168	145	120	138	77	154	108	127	124	90
	75th percentile	304	248	184	235	146	307	203	224	251	182
	90th percentile	365	365	326	365	248	365	312	365	365	357
Total interval (All patients)	Number	(n=222)	(n=221)	(n=223)	(n=209)	(n=249)	(n=148)	(n=224)	(n=238)	(n=262)	(n=205)
	Median	128	112	103	111	77	136	102	127	105	74
	75th percentile	239	201	159	211	146	266	194	224	230	153
	90th percentile	365	365	253	365	248	365	307	365	365	320

For peer review only - http://bmjopen.bmj.com/site/about/guidelines.xhtml

Table 6. *Analyses of the differences in intervals (days) between Wales as the reference and the other nine jurisdictions.*

		Wales	England	Scotland	N Ireland	Denmark	Manitoba	Norway	Sweden	Ontario	Victoria
Patient Interval (Symptomatic patients)	Number	(n=195)	(n=199)	(n=175)	(n=199)	(n=292)	(n=134)	(n=240)	(n=266)	(n=230)	(n=199)
	Median	49 (ref.)	-6 (-25,13)	-14 (-29,2)	-11 (-42,20)	**-25** (-38,-11)	-11 (-30,9)	-9 (-46,27)	-7 (-21,7)	-13 (-30,4)	**-23** (-32,-15)
	75th percentile	92 (ref.)	14 (-9,38)	**-28** (-44,-12)	-13 (-32,6)	**-28** (-47,-9)	1 (-28,30)	-3 (-22,16)	0 (-29,29)	11 (-8,29)	**-30** (-51,-9)
	90th percentile	249 (ref.)	23 (-14,60)	**-74** (-124,-24)	**26** (10,42)	-60 (-174,55)	-17 (-65,31)	-43 (-85,0)	**-43** (-60,-27)	3 (-109,115)	-33 (-87,21)
Primary Care interval (Symptomatic patients)	Number	(n=157)	(n=152)	(n=127)	(n=160)	(n=207)	(n=72)	(n=124)	(n=0)	(n=77)	(n=117)
	Median	3 (ref.)	-2 (-5,2)	1 (-4,6)	-3 (-5,0)	-2 (-5,1)	0 (-8,8)	**7** (3,12)	n/a	-2 (-5,1)	6 (0,13)
	75th percentile	20 (ref.)	-1 (-14,12)	9 (-6,25)	-7 (-20,6)	-10 (-25,4)	9 (-3,21)	**21** (3,39)	n/a	3 (-8,15)	13 (-6,31)
	90th percentile	78 (ref.)	-14 (-31,3)	**42** (11,73)	-23 (-64,17)	-19 (-91,53)	**124** (63,186)	36 (0,71)	n/a	3 (-43,49)	36 (-16,88)
Diagnostic interval (Symptomatic patients)	Number	(n=194)	(n=196)	(n=174)	(n=190)	(n=290)	(n=133)	(n=229)	(n=249)	(n=218)	(n=197)
	Median	60 (ref.)	**-11** (-18,-4)	**-20** (-27,-13)	5 (-6,16)	**-29** (-35,-24)	14 (-1,29)	**-20** (-28,-12)	**-19** (-35,-3)	-1 (-11,8)	**-28** (-35,-21)
	75th percentile	155 (ref.)	**-56** (-119,7)	**-59** (-113,-5)	**-44** (-81,-7)	**-83** (-110,-55)	-6 (-60,47)	**-67** (-93,-42)	**-65** (-117,-13)	-3 (-52,46)	**-81** (-131,-32)
	90th percentile	284 (ref.)	**-78** (-116,-40)	**-114** (-132,-96)	**-54** (-89,-19)	**-130** (-161,-99)	**33** (9,56)	**-80** (-114,-46)	**-86** (-116,-56)	15 (-2,32)	**-78** (-103,-53)
Diagnostic interval (Screen-detected patients)	Number	(n=69)	(n=56)	(n=76)	(n=35)	(n=0)	(n=25)	(n=14)	(n=0)	(n=50)	(n=38)
	Median	35 (ref.)	**-8** (-13,-4)	3 (-2,8)	**-32** (-36,-28)	n/a	**31** (19,43)	**-15** (-24,-5)	n/a	**-25** (-34,-15)	1 (-8,10)
	75th percentile	65 (ref.)	**-18** (-28,-8)	-12 (-29,4)	**-63** (-70,-55)	n/a	**36** (6,66)	**-24** (-42,-6)	n/a	**-17** (-26,-8)	6 (-20,33)
	90th percentile	99 (ref.)	**-17** (-28,-6)	12 (-16,41)	**-98** (-101,-96)	n/a	**90** (32,147)	**-35** (-43,-27)	n/a	**40** (30,51)	**32** (25,40)
Diagnostic interval (All patients)	Number	(n=263)	(n=252)	(n=250)	(n=225)	(n=290)	(n=158)	(n=243)	(n=249)	(n=268)	(n=235)
	Median	52 (ref.)	**-9** (-15,-4)	**-13** (-19,-7)	-2 (-12,7)	**-22** (-28,-17)	**21** (13,29)	**-14** (-24,-3)	**-13** (-21,-4)	-4 (-11,2)	**-21** (-26,-15)
	75th percentile	120 (ref.)	**-34** (-49,-18)	**-38** (-53,-23)	-10 (-31,10)	**-44** (-56,-32)	**22** (4,41)	**-29** (-50,-9)	**-27** (-38,-17)	**18** (2,34)	**-46** (-57,-34)
	90th percentile	242 (ref.)	**-73** (-101,-45)	**-91** (-118,-64)	**-41** (-57,-24)	**-106** (-127,-85)	**50** (7,94)	**-53** (-88,-18)	**-59** (-88,-30)	**44** (27,61)	**-54** (-82,-26)
Treatment interval (Symptomatic patients)	Number	(n=197)	(n=206)	(n=185)	(n=208)	(n=306)	(n=161)	(n=258)	(n=281)	(n=248)	(n=209)
	Median	39 (ref.)	**-6** (-11,-1)	-5 (-11,0)	**-13** (-19,-8)	**-24** (-27,-21)	-5 (-14,5)	**-20** (-23,-16)	-3 (-8,2)	**-6** (-10,-1)	**-23** (-28,-19)
	75th percentile	59 (ref.)	**-13** (-18,-8)	-4 (-11,4)	**-19** (-24,-13)	**-41** (-48,-34)	0 (-9,10)	**-30** (-37,-24)	**-8** (-16,-1)	-6 (-12,0)	**-31** (-38,-25)
	90th percentile	83 (ref.)	**-27** (-38,-16)	-5 (-20,11)	**-29** (-42,-17)	**-61** (-72,-50)	4 (-13,20)	**-43** (-59,-27)	**-24** (-35,-14)	-8 (-21,4)	**-42** (-52,-31)
Treatment interval (Screen-detected patients)	Number	(n=72)	(n=58)	(n=78)	(n=34)	(n=0)	(n=79)	(n=17)	(n=0)	(n=52)	(n=60)
	Median	44 (ref.)	-4 (-14,5)	3 (-6,12)	-5 (-15,5)	n/a	-8 (-17,2)	**-28** (-41,-16)	n/a	**-8** (-14,-2)	**-26** (-36,-16)
	75th percentile	68 (ref.)	**-22** (-26,-19)	0 (-5,5)	**-22** (-25,-18)	n/a	**-13** (-22,-3)	**-45** (-49,-42)	n/a	**-23** (-26,-20)	**-37** (-45,-29)
	90th percentile	80 (ref.)	**-21** (-32,-9)	6 (-3,14)	**-23** (-31,-15)	n/a	-6 (-15,3)	**-37** (-45,-30)	n/a	11 (-1,22)	**-42** (-51,-33)
Treatment interval (All patients)	Number	(n=271)	(n=268)	(n=266)	(n=246)	(n=312)	(n=240)	(n=279)	(n=284)	(n=310)	(n=276)
	Median	41 (ref.)	**-6** (-10,-2)	-2 (-9,5)	**-13** (-18,-9)	**-26** (-30,-21)	-4 (-10,1)	**-22** (-27,-16)	-5 (-10,0)	**-6** (-11,-1)	**-24** (-29,-20)
	75th percentile	63 (ref.)	**-15** (-21,-8)	2 (-10,14)	**-19** (-27,-11)	**-42** (-50,-35)	0 (-7,7)	**-32** (-39,-24)	**-9** (-16,-1)	-8 (-17,2)	**-32** (-46,-17)
	90th percentile	80 (ref.)	**-24** (-31,-17)	3 (-12,18)	**-25** (-34,-17)	**-59** (-66,-51)	4 (-4,12)	**-41** (-54,-29)	**-22** (-30,-15)	-4 (-19,11)	**-40** (-50,-30)
Total interval (Symptomatic patients)	Number	(n=154)	(n=165)	(n=147)	(n=175)	(n=249)	(n=123)	(n=210)	(n=238)	(n=214)	(n=168)
	Median	168 (ref.)	**-30** (-55,-6)	**-52** (-99,-5)	-36 (-93,21)	**-92** (-106,-78)	-25 (-86,35)	**-63** (-74,-52)	**-43** (-63,-24)	-44 (-98,9)	**-85** (-97,-73)
	75th percentile	304 (ref.)	-35 (-78,8)	**-101** (-130,-71)	-34 (-91,22)	**-137** (-177,-97)	-10 (-24,4)	**-84** (-129,-38)	**-62** (-92,-32)	-44 (-91,2)	**-117** (-137,-97)
	90th percentile	365 (ref.)	0 (-1,0)	**-39** (-47,-32)	0 (-1,0)	**-125** (-134,-116)	0 (-1,0)	**-49** (-58,-41)	0 (-1,0)	0 (-1,0)	-7 (-16,1)
Total interval (All patients)	Number	(n=222)	(n=221)	(n=223)	(n=209)	(n=249)	(n=148)	(n=224)	(n=238)	(n=262)	(n=205)
	Median	128 (ref.)	-13 (-35,9)	**-22** (-36,-8)	-14 (-38,10)	**-46** (-62,-30)	10 (-13,32)	**-19** (-34,-5)	-1 (-16,15)	**-18** (-34,-2)	**-44** (-70,-18)
	75th percentile	239 (ref.)	**-32** (-63,-1)	**-81** (-126,-35)	-26 (-64,12)	**-89** (-137,-41)	10 (-33,52)	**-43** (-70,-17)	-18 (-59,23)	-16 (-80,49)	**-88** (-119,-57)
	90th percentile	365 (ref.)	-1 (-2,0)	**-108** (-129,-88)	-1 (-3,1)	**-118** (-127,-109)	0 (-2,2)	**-56** (-80,-32)	0 (-1,0)	0 (-2,1)	**-46** (-54,-37)

For peer review only - http://bmjopen.bmj.com/site/about/guidelines.xhtml

The differences for the 50th (median), 75th and 90th percentiles are calculated as marginal effects after quantile regression by setting the continuous covariate age to its mean value and categorical covariates (gender and comorbidity) to their modes. The actual number of days are included for Wales in Table 5.

For peer review only

For peer review only - http://bmjopen.bmj.com/site/about/guidelines.xhtml

The median primary care interval was 12 days in Norway (Table 5), statistically significantly longer than Wales (Table 6). For the 10% of patients waiting longest for referral, the longest intervals were observed in Manitoba, Victoria and Scotland (210, 128 and 93 days, respectively) (Table 5). This interval at the 90th percentile was either 4 months (Manitoba) or 1 month (Victoria and Scotland) longer than in Wales (Table 6).

The median diagnostic interval for symptomatic patients ranged from 27-28 days in Denmark and Victoria to 76 days in Manitoba. At the 90th percentile it ranged from 4 months in Denmark to 10 months in Ontario (Table 5). All jurisdictions had shorter median diagnostic intervals compared to Wales, except Northern Ireland and Manitoba, where the intervals were 5 and 14 days longer, respectively (Table 6).

The shortest median treatment intervals for all patients (about 2 weeks) were observed in Denmark, Victoria and Norway. In other jurisdictions this interval was 1 month or more (Table 5). All jurisdictions had shorter treatment intervals compared to Wales, except Scotland and Manitoba (Table 6).

The median total interval (from first symptom to treatment start) for all CRC patients was between 74 days (Victoria) and 136 days (Manitoba) (Table 5). In Scotland, Denmark, Norway and Victoria, this interval was statistically significantly shorter than in Wales (Table 6).

Sensitivity and validity analyses

Changing the cut-off survey completion date from 9 months post-diagnosis to the per-protocol 6 months changed the number of included patients. However, the estimates of routes to diagnosis and time intervals were not significantly altered and the trend was the same as in the main analyses (results not shown). Sensitivity analysis based solely on patient data for those whose reporting on all time intervals was complete did not change the trend (results not shown). Comparing patient and PCP reported routes (screening and symptomatic presentation) and registry data on screening showed that agreement ranged from moderate to almost perfect (Kappa 0.59-0.86).

Comparing the dates between the different data sources showed a high agreement between all data sources for all categories of dates (CCC $\geq$ 0.95 for date of diagnosis, CCC = 0.94 for date of treatment and CCC = 0.92 for date of first presentation to primary care). The analysis of the ICBP M4 definition of screening route compared with registry data showed an almost perfect agreement (Kappa>0.80) in two jurisdictions and substantial (Kappa>0.70) in two jurisdictions (Table 3).

For peer review only - http://bmjopen.bmj.com/site/about/guidelines.xhtml

DISCUSSION

Our study showed marked variation in the proportion of screen-detected cancers, lengths of diagnostic and treatment (and total) intervals between jurisdictions. Patient intervals were shortest in Denmark and longest in Wales; longer primary care intervals were present in Norway, Scotland, Manitoba and Victoria. Differences in primary care intervals do not necessarily reflect PCP delay - they may arise from PCPs undertaking more investigations prior to referral. Overall, the differences are marked and suggest the need, in some jurisdictions, for revised diagnostic pathways to reduce the time taken for patients to be diagnosed and treated.

The interval differences did not show an obvious association with earlier reported survival differences – jurisdictions with poorer survival did not consistently show longer intervals, and vice versa.[4]] While this may question the validity of our findings, and/or the relationship between diagnostic intervals and survival, it is important to note these analyses were several years apart, and there may have been significant health system changes since the survival comparisons. Nevertheless, our study adds to a growing body of evidence on routes to diagnosis and time intervals; there are few similar examples involving multiple countries in the literature.[29]

Many factors underpin the differences observed between jurisdictions, such as structural differences in healthcare delivery (e.g. care pathways, availability and accessibility of diagnostic and treatment facilities, etc.). Differences in routes to diagnosis were influenced by the presence or absence of screening programmes (in Denmark and Sweden CRC-screening had not been implemented at the time of study). Patient interval variation may reflect differences in symptom awareness or health-seeking behaviour. However, a study which compared awareness of cancer symptoms, attitudes towards cancer and barriers to attending a PCP did not demonstrated statistically significant differences.[30] It is possible that other factors, such as culture, rurality, economic or patient-specific barriers and facilitators, influence this important part of the pathway.[31]

There were significant differences in primary care intervals; overall these intervals were much longer than those found in our breast cancer analyses.[32] This raises important questions about diagnostic processes within primary care. There are widespread calls for PCPs to play a greater role in improving outcomes in CRC.[33] Indeed, it is now widely acknowledged that primary care has a major role in cancer control at all stages of the patient journey.[34] Nevertheless, there is some evidence based on observational associations that prolonged intervals might be associated with stronger 'gatekeeper' systems.[35] Further, independent of the nature of symptoms, investigation in primary care has been noted to be associated with later referral for specialist assessment for CRC and other cancers - so a long primary care interval may mean that PCPs are doing more before they refer[36]. Hence, PCP access to and use of investigations and differing national

For peer review only - http://bmjopen.bmj.com/site/about/guidelines.xhtml

cancer referral guidance may influence primary care intervals. There were differences in diagnostic intervals, suggesting that once patients have been referred to secondary care there is considerable variation in their experiences; differences in treatment intervals were less marked. These variations suggest there is room for improvement in reducing the total interval and its various sub-intervals, and that guidance on optimal pathways should be better implemented. Each participating jurisdiction will likely draw unique conclusions about the most appropriate response to our findings.

Strengths and weaknesses

A major strength of this study is its use of standardised survey methods in a broad range of jurisdictions to systematically examine the various components of these intervals and to describe and compare, between countries, patient journeys to a cancer diagnosis and treatment. To ensure comparability across jurisdictions, our surveys drew on existing instruments and went through an extensive process of cognitive testing, piloting and translation and adaptation.[18]

Data quality was enriched by information from national cancer registries and our algorithms showed very good agreement for jurisdictions where validation was possible. Using validated identification of CRC patients minimised the risk of missing cancer cases during inclusion and of selection bias. Further, the use of registries made it possible to exclude patients with previous cancer in the same site, providing a homogeneous group of newly diagnosed CRC patients in need of diagnostic work-up.

It was evident that there were subtle differences in the understanding of 'screening' between jurisdictions. Patients do not always distinguish between tests for screening and those for symptom-based diagnosis. Including data from registries and triangulating patient and PCP data enhanced the validity of 'screen-detected versus non-screen-detected' information, but the underlying factors varied between jurisdictions – for example, in Australia PCPs often provide screening FOBTs during consultations whereas this is rare in the UK and Scandinavia. To counter these inconsistencies, we applied our validated data rules which showed a high agreement with screening registries.

There are inevitable differences in questionnaire interpretation, characteristics of non-responders and availability of supplementary data for validation purposes. There are always considerations with questionnaire interpretation but the methodology and analysis of data sought to minimise or account for this as much as possible. Further, we used triangulation and comprehensive data rules to ensure validity, consistency and preserve statistical precision.[18] We included registry data where possible (screening, stage, date of diagnosis) and developed reliable rules for imputation based on these registry data. To minimise misclassification from data entry and handling, data entry was internally audited by local teams and data interpretation was reduced to an absolute minimum and only performed centrally. Recall bias was

For peer review only - http://bmjopen.bmj.com/site/about/guidelines.xhtml

minimised by the triangulation of different data sources and by ensuring that the patients received the questionnaire with a limited time window after the cancer diagnosis.

The overall response of 31% for the patient survey varied between jurisdictions. There were likely differences in the selection bias in individual jurisdictions; Our patient sampling strategy will have led to some differences in the composition of our samples, as some patients were included directly from registries, some via PCPs and, in Northern Ireland, research nurses checked lists of potentially eligible patients to confirm eligibility. We have no mechanism to examine the direction of such possible selection bias. However, comparison of participating patients on a number of variables (including comorbidity, self-assessed health, smoking, stage, presenting symptom) did not show obvious differences with potential to bias our results. We also compared age, sex and stage of cancer amongst participants versus eligible patients and found no significant differences. There were different classification systems for ethnicity and education across jurisdictions which would lead to biased estimates if included in the regression model, even if mapped or harmonised – hence, they were excluded. There were few respondents from minority ethnic groups, limiting the generalisability of our findings; further work should target these groups as they are likely to have unique characteristics in their routes to diagnosis.

Confounding from aspects related to the diagnostic route for CRC was diminished by adjusting for age, gender and comorbidity. It is possible that there is some residual confounding which can bias the results in different directions. The statistical precision of the study was high as we were able to show clinically significant differences of one week in time intervals.

Comparison with other studies

Other studies have examined symptoms and routes to diagnosis for patients with CRC – although rarely in more than a single setting. A UK study on patients diagnosed in 2001-2002 and 2007-2008 reported median diagnostic intervals of 100 and 80 days respectively.[37] A Danish study showed median diagnostic intervals for CRC of between 31 and 55 days, depending on the timing of measurement in relation to implementation of pathway guidance.[38] The difference between the present study and the former may stem from methodology issues, especially data sources (i.e. databases vs. surveys).

A study in Spain showed a symptom to diagnosis interval for CRC of 128 days and symptom to treatment interval of 155 days – these authors found that nature of symptoms, perceived seriousness of symptoms by patients, and place of first presentation influenced diagnostic and treatment intervals.[39] Sampling strategies and survey differences will have influenced the results, making it difficult to compare these studies; nevertheless, they confirm that our results are broadly consistent with previous, single-jurisdiction studies.

For peer review only - http://bmjopen.bmj.com/site/about/guidelines.xhtml

The pattern of symptoms in ICBP participants was similar to other studies.[40] CRC is known to be a cancer that clinically presents with either 'alarm' symptoms, or more vague symptoms; there is evidence that doctors and patients respond less promptly to some symptoms of CRC than others – and that this can be influenced by the presence of co-morbid conditions.[41] However, the minor differences in symptom patterns seen in Table 4 are insufficient to explain the between-jurisdiction variation we have demonstrated in routes to diagnosis and diagnostic, treatment and total intervals.

Explaining observed differences between jurisdictions

The variation we see between jurisdictions mostly derives from differences in the extent to which healthcare systems support expedited CRC diagnosis and treatment; indeed, some health system characteristics, such as access and patient mobility between healthcare providers, may influence cancer outcomes - although these factors require further exploration.[42] In Denmark there have been a number of reforms specifically designed to reduce diagnostic intervals.[43] This study indicates a potential to optimise diagnostic routes for CRC in some jurisdictions. This should ideally be in conjunction with screening efforts which is gaining traction across many Western countries in response to policy and guideline initiatives.[44]

CONCLUSION

This study demonstrates considerable absolute and relative differences between jurisdictions in time intervals from first symptom until treatment for CRC. These differences do not demonstrate an obvious relationship with existing ICBP survival differences between the jurisdictions. ICBP phase 2 will report survival estimates to 2014, at which point it may be possible to explore the relationship between interval lengths and survival estimates in the population. Further work is ongoing to explore the outcome of patients included in this ICBP M4 study alongside the interval lengths observed.

The median total interval, which varied between 74 and 136 days, indicates that there is unrealised potential to optimise pathways for CRC. The main differences were found for structural parts of the pathway (e.g. those not relating to patient behaviours/actions). Further, there is a 'tail' of patients waiting many months longer to start treatment for their cancer which may affect their outcomes. While our study highlights important international differences in routes to diagnosis, further research is needed to understand these differences, and elucidate the contribution of patient pathway guidance and implementation, and health system structures. Nevertheless, the data provide important prompts for jurisdictions and suggest considerable room for improvement in some areas; they will also serve as a benchmark for measuring the effectiveness of future interventions.

For peer review only - http://bmjopen.bmj.com/site/about/guidelines.xhtml

For peer review only

For peer review only - http://bmjopen.bmj.com/site/about/guidelines.xhtml

List of abbreviations

ICBP M4 – International Cancer Benchmarking Partnership Module 4

PCP – primary care physician

CTS – cancer treatment specialist

CRC – colorectal cancer

Acknowledgements

We thank our colleagues and participating patients, primary care physicians and cancer treatment specialists in all ICBP jurisdictions. Catherine Foot, Martine Bomb, Brad Groves, Irene Reguilon and Samantha Harrison of Cancer Research UK for managing the programme. John Butler, Royal Marsden NHS Foundation Trust and ICBP Clinical Lead, for his advice. The ICBP M4 academic reference group (Supplementary File 2) for providing independent peer review and advice for the study protocol and analysis plan development. Throughout the study invaluable guidance was provide by the ICBP Programme Board. Membership of this board: Aileen Keel (Scottish Government, Edinburgh, Scotland); Anna Boltong (Cancer Council Victoria, Carlton, Australia); Anna Gavin (Northern Ireland Cancer Registry, Queens University, Belfast, UK); David Currow (Cancer Institute New South Wales, Sydney, Australia); Gareth Davies (Wales Cancer Network, Cardiff, UK); Gunilla Gunnarsson (Swedish Association of Local Authorities and Regions, Stockholm, Sweden); Heather Bryant (Canadian Partnership Against Cancer, Toronto, Canada); Jane Hanson (Welsh Cancer National Specialist Advisory Group, Cardiff, UK); Kathryn Whitfield (Department of Health, Victoria Australia); Linda Rabeneck (Cancer Care Ontario, Toronto, Canada); Michael A Richards (Care Quality Commission, London, UK); Michael Sherar (Cancer Care Ontario, Toronto, Canada); Nicola Quin (Cancer Council Victoria, Carlton, Australia); Nicole Mittmann (Cancer Care Ontario, Toronto, Canada); Robert Thomas (Department of Health and Human Services, Victoria, Melbourne, Australia); Sara Hiom (Cancer Research UK); Sean Duffy (NHS England, London, UK); Chris Harrison (NHS England, London, UK); Søren Brostrøm (Danish Health and Medicines Authority, Copenhagen, Denmark); Stein Kaasa (University Hospital of Trondheim, Trondheim, Norway).

Contributorship statement

DW, PV, UM, AZF, HJ planned the study design, data collection, carried out the analyses and wrote the draft manuscript. DW, PV, UM, HJ, AB, AKK, RJB, DHB, VC, ATG, EG, EH, ML, RJW, YL, MM, DT, RDN, VW, IR and SH were responsible for local data collection (alongside the ICBP Module 4 Working Group), management and interpretation, and have participated in writing and approving the final manuscript version.

Competing Interests

None

Funding

This work was supported by: CancerCare Manitoba; Cancer Care Ontario; Canadian Partnership Against Cancer (CPAC); Cancer Council Victoria; Cancer Research Wales; Cancer Research UK; Danish Cancer

For peer review only - http://bmjopen.bmj.com/site/about/guidelines.xhtml

Society; Danish Health and Medicines Authority; European Palliative Care Research Centre (PRC), Norwegian University of Science and Technology (NTNU); Northern Ireland Guidelines Audit and Implementation Network (GAIN); Macmillan Cancer Support; National Cancer Action Team; NHS England; Northern Ireland Cancer Registry, funded by the Public Health Agency NI; Norwegian Directorate of Health; Research Centre for Cancer Diagnosis in Primary Care (CaP), Aarhus University, Denmark; Scottish Government; Swedish Association of Local Authorities and Regions; University College London and NIHR Biomedical Research Centre at University College London NHS Foundation Trust; University of Edinburgh; Victorian Department of Health and Human Services; Welsh Government. The funding bodies had no influence on the design of the study and collection, analysis, and interpretation of data, in writing the manuscript or whether to publish the results.

Availability of data and material

The data that support the findings of this study are available from the named authors from each ICBP jurisdiction, but restrictions apply to the availability of these data and so are not publicly available. Data are however available from the authors upon reasonable request and with permission of the ICBP Programme Board. Please contact the ICBP Programme Management team, based at Cancer Research UK, with any queries (icbp@cancer.org.uk).

Ethics approval and consent to participate

For each local data collection, there were specific procedures and approvals which included anonymised data transfer to University College London and Aarhus University (Supplementary file 5). Approvals were received from the following institutions: Cancer Council Victoria Human Research Ethics Committee [HREC 1125]; Health Research Ethics Board, University of Manitoba [HS15227 (H2012:105)]; Research Resource Ethics Committee, CancerCare Manitoba [RRIC#28-2012]; University of Toronto Research Ethics Board [27881]; The Danish Data Protection Agency [2013-41-2030]; Swedish Ethics Review Board, Uppsala [2013/306]; Norway Regional committees for medical and health research ethics [2013/136/REK nord]; England, Wales and Scotland, NRES Committee East Midlands – Derby 2, local R&D for each health board [11/EM/0420]; Northern Ireland ORECNI Ethical approval, local governance for each health Trust [11/EM/0420].

For peer review only - http://bmjopen.bmj.com/site/about/guidelines.xhtml

References

1 Ferlay J, Soerjomataram I, Dikshit R, et al. Cancer incidence and mortality worldwide: sources, methods and major patterns in GLOBOCAN 2012. *International journal of cancer* 2015 Mar 1;136(5):E359-86. doi: 10.1002/ijc.29210

2 Brenner H, Chang–Claude J, Jansen L, et al. Reduced risk of colorectal cancer up to 10 years after screening, surveillance, or diagnostic colonoscopy. *Gastroenterology* 2014 Mar 31;146(3):709-17. doi: 10.1053/j.gastro.2013.09.001.

3 De Angelis R, Sant M, Coleman MP, et al. Cancer survival in Europe 1999–2007 by country and age: results of EUROCARE-5—a population-based study. *Lancet Oncol* 2014 Jan 31;15(1):23-34. doi: 10.1016/S1470-2045(13)70546-1

4 Coleman MP, Forman D, Bryant H, et al. Cancer survival in Australia, Canada, Denmark, Norway, Sweden, and the UK, 1995-2007 (the International Cancer Benchmarking Partnership): an analysis of population-based cancer registry data. *Lancet* 2011;377:127-38 doi:10.1016/S0140-6736(10)62231-3

5 Maringe C, Walters S, Rachet B, et al. Stage at diagnosis and colorectal cancer survival in six high-income countries: a population-based study of patients diagnosed during 2000-2007. *Acta Oncol* 2013;52(5):919-32. doi: 10.3109/0284186X.2013.764008

6 Butler J, Foot C, Bomb M, et al. The international cancer benchmarking partnership: an international collaboration to inform cancer policy in Australia, Canada, Denmark, Norway, Sweden and the United Kingdom. *Health Policy* 2013 Sep 30;112(1):148-55. doi:10.1016/j.healthpol.2013.03.021

7 Hamilton W. Cancer diagnosis in primary care. *Br J Gen Pract* 2010 Feb 1;60(571):121-8. doi: 10.3399/bjgp10X483175.

8 Elliss-Brookes L, McPhail S, Ives A, et al. Routes to diagnosis for cancer - determining the patient journey using multiple routine data sets. *Br J Cancer* 2012 Oct 9;107(8):1220-6. doi: 10.1038/bjc.2012.408.

9 Hansen RP, Vedsted P, Sokolowski I, et al. Time intervals from first symptom to treatment of cancer: a cohort study of 2,212 newly diagnosed cancer patients. *BMC Health Serv Res* 2011 Oct 25;11(1):1. doi: 10.1186/1472-6963-11-284.

10 Emery JD, Shaw K, Williams B, et al. The role of primary care in early detection and follow-up of cancer. *Nat Rev Clin Oncol* 2014 Jan;11(1):38-48. doi: 10.1038/nrclinonc.2013.212.

11 Vedsted P, Olsen F. Early diagnosis of cancer-the role of general practice. *Scand J Prim Health Care* 2009;27(4):193-4. doi: 10.3109/02813430903478623.

12 Jensen H, Tørring ML, Olesen F, et al. Cancer suspicion in general practice, urgent referral and time to diagnosis: a population-based GP survey and registry study. *BMC Cancer* 2014 Aug 30;14:636. doi: 10.1186/1471-2407-14-636. doi: 10.1186/1471-2407-14-636

13 Tørring ML, Frydenberg M, Hamilton W, et al. Diagnostic interval and mortality in colorectal cancer: U-shaped association demonstrated for three different datasets. *J Clin Epidemiol* 2012;65(6):669-78. doi: 10.1016/j.jclinepi.2011.12.006

14 Neal RD, Tharmanathan P, France B, et al. Is increased time to diagnosis and treatment in symptomatic cancer associated with poorer outcomes; Systematic review. *Br J Cancer* 2015 Mar 31;112:S92-107. doi: 10.1038/bjc.2015.48.

For peer review only - http://bmjopen.bmj.com/site/about/guidelines.xhtml

15 Rose PW, Rubin G, Perera-Salazar R, et al. Explaining variation in cancer survival between 11 jurisdictions in the International Cancer Benchmarking Partnership: a primary care vignette survey. *BMJ Open* 2015 May 1;5(5):e007212. doi: 10.1136/bmjopen-2014-007212

16 Brenner H, Bouvier AM, Foschi R, et al. Progress in colorectal cancer survival in Europe from the late 1980s to the early 21st century: the EUROCARE study. *International Journal of Cancer* 2012 Oct 1;131(7):1649-58. doi: 10.1002/ijc.26192

17 Cancer Research UK, http://www.cancerresearchuk.org/health-professional/cancer-statistics/statistics-by-cancer-type/bowel-cancer/diagnosis-and-treatment#heading-Zero, Accessed January 2018.

18 Weller D, Vedsted P, Anandan C, et al. An investigation of routes to cancer diagnosis in 10 international jurisdictions, as part of the International Cancer Benchmarking Partnership: survey development and implementation. *BMJ Open* 2016 Jul 1;6(7):e009641. doi: 10.1136/bmjopen-2015-009641

19 Weller D, Vedsted P, Rubin G, et al. The Aarhus statement: improving design and reporting of studies on early cancer diagnosis. *Br J Cancer* 2012 Mar 27;106(7):1262-7. doi: 10.1038/bjc.2012.68

20 World Health Organisation. International Classification of Diseases and Related Health Problems (ICD-10). WHO, Geneva, 2016

21 European Network of Cancer Registries. Standards and guidelines for cancer registration in Europe: the ENCR recommendations. Edited by Tyczynski JE, Démaret E, Parkin DM. (IARC technical publication; no. 40), Lyon, 2003

22 Sobin LH, Fleming ID. TNM classification of malignant tumors, (1997). *Cancer* 1997 Nov 1;80(9):1803-4

23 Jenkinson C, Coulter A, Wright L. Short form 36 (SF36) health survey questionnaire: normative data for adults of working age. *BMJ* 1993 May 29;306(6890):1437-40

24 Nicholson BD, Mant D, Neal RD, et al. International variation in adherence to referral guidelines for suspected cancer: a secondary analysis of survey data. *Br J Gen Pract* 2016 Feb 1;66(643):e106-13. doi: 10.3399/bjgp16X683449.

25 Koenker R, Bassett G. Regression quantiles. *Econometrica* 1978; 46:33-50

26 Miranda A. QCOUNT: Stata program to fit quantile regression models for count data. Boston: Boston College Department of Economics; 2006

27 Landis J, Koch G. The measurement of observer agreement for categorical data. *Biometrics* 1997;33(1): 159-174

28 Lin LI. A concordance correlation coefficient to evaluate reproducibility. *Biometrics* 1989;45((1) March):255–68

29 Murchie P, Campbell NC, Delaney EK, et al. Comparing diagnostic delay in cancer: a cross-sectional study in three European countries with primary care-led health care systems. *Family Practice* 2012, *29*(1), 69-78. doi: 10.1093/fampra/cmr044

30 Forbes LJ, Simon AE, Warburton F, et al. Differences in cancer awareness and beliefs between Australia, Canada, Denmark, Norway, Sweden and the UK (the International Cancer Benchmarking Partnership): do they contribute to differences in cancer survival? *Br J Cancer* 2013;108:292-300 doi:10.1038/bjc.2012.542

For peer review only

31 Parsonage RK, Hiscock J, Law RJ, et al. Patient perspectives on delays in diagnosis and treatment of cancer: a qualitative analysis of free-text data. *Br J Gen Pract* 2017; 67 (654): e49-e56. DOI: 10.3399/bjgp16X688357

32 Vedsted P, Weller D, Falborg AZ, et al. Diagnostic routes and time intervals for breast cancer in 10 jurisdictions. The international cancer benchmarking partnership (ICBP) 2018 (undergoing submission process)

33 Rubin G, Weller D. Improving survival in colorectal cancer: what role for general practice? *Br J Gen Pract* 2013 Apr 1;63(609):179-80

34 Rubin G, Berendsen A, Crawford SM, et al. The expanding role of primary care in cancer control. *Lancet Oncol* 2015 Sep 30;16(12):1231-72 - a

35 Vedsted P, Olesen F. Are the serious problems in cancer survival partly rooted in gatekeeper principles? An ecologic study. *Br J Gen Pract* 2011 Aug 1;61(589):e508-12

36 Rubin GP, Saunders CL, Abel GA, et al. Impact of investigations in general practice on timeliness of referral for patients subsequently diagnosed with cancer: analysis of national primary care audit data. British journal of cancer 2015 Feb 17;112(4):676-87 – b

37 Neal RD, Din NU, Hamilton W, et al. Comparison of cancer diagnostic intervals before and after implementation of NICE guidelines: analysis of data from the UK General Practice Research Database. *Br J Cancer* 2014 Feb 4;110(3):584-92.

38 Jensen H, Tørring ML, Olesen F, et al. Diagnostic intervals before and after implementation of cancer patient pathways–a GP survey and registry based comparison of three cohorts of cancer patients. *BMC cancer* 2015 Apr 23;15(1):1

39 Esteva M, Leiva A, Ramos M, et al. Factors related with symptom duration until diagnosis and treatment of symptomatic colorectal cancer. *BMC cancer* 2013 Feb 23;13(1):1

40 Astin M, Griffin T, Neal RD, et al. The diagnostic value of symptoms for colorectal cancer in primary care: a systematic review. *Br J Gen Pract* 2011 May 1;61(586):e231-43

41 Walter FM, Emery J, Mendonca S, et al. Symptoms and co-morbidities associated with diagnostic intervals for colorectal cancer: a prospective cohort study. *Eur J Cancer Care* 2015 Sep 1;24:49

42 Brown S, Castelli M, Hunter DJ, et al. How might healthcare systems influence speed of cancer diagnosis: a narrative review. *Soc Sci Med* 2014 Sep 30;116:56-63

43 Vedsted P, Olesen F. A differentiated approach to referrals from general practice to support early cancer diagnosis–the Danish three-legged strategy. *Br J Cancer* 2015 Mar 31;112(Suppl 1):S65.

44 Altobelli E, Lattanzi A, Paduano R, et al. Colorectal cancer prevention in Europe: burden of disease and status of screening programs. *Prev Med 2014* May 31;62:132-41

For peer review only - http://bmjopen.bmj.com/site/about/guidelines.xhtml

Figure 1 – Survival differences for colorectal cancer demonstrated in the International Cancer Benchmarking Partnership Module 1.[4]

Figure 2 – Diagnosis and treatment of colorectal cancer: Illustration of key time points and intervals.[19]

Supplementary File 1 – Data rules

Supplementary File 2 – Sample CRC patient questionnaire

Supplementary File 3 – Sample CRC primary care questionnaire

Supplementary File 4 – Sample CRC specialist questionnaire

Supplementary File 5 – Ethical approval, Working and Academic Reference Groups

Supplementary File 6 – Regression analysis diagrams (based on Table 6)

For peer review only

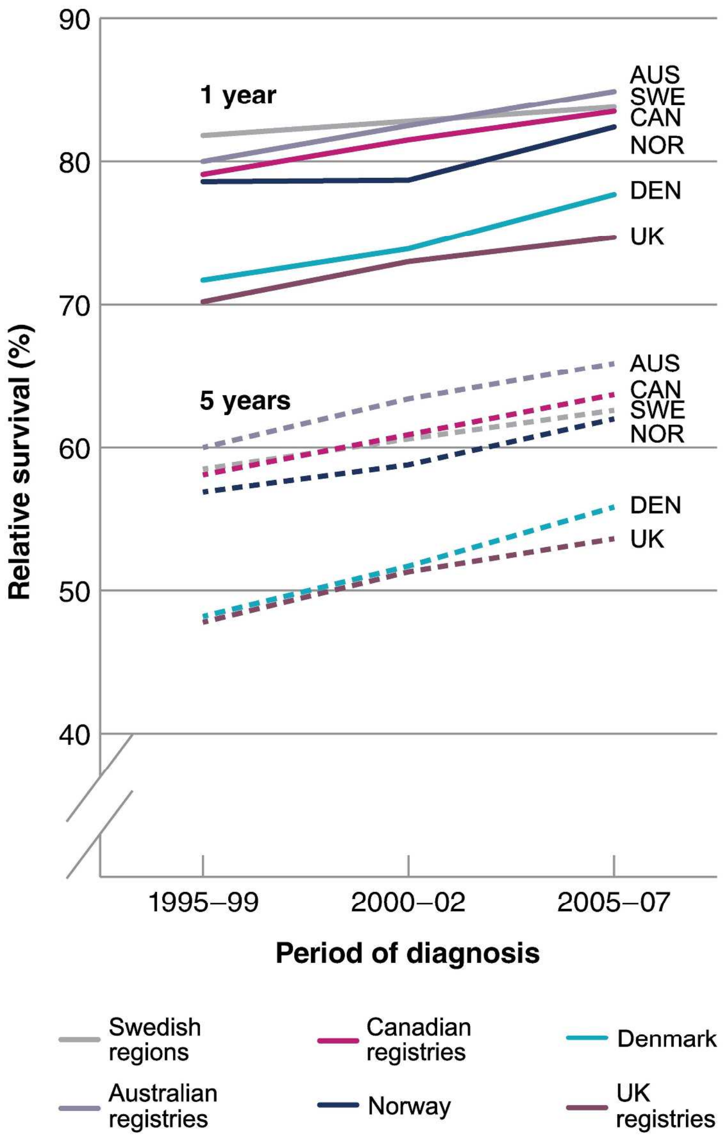

206x312mm (300 x 300 DPI)

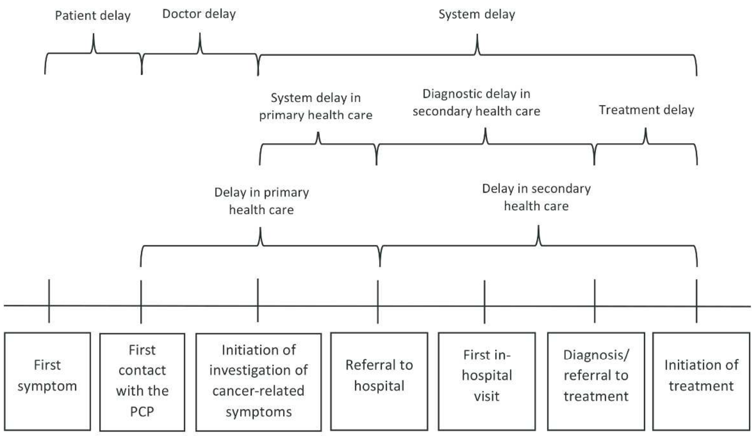

209x297mm (300 x 300 DPI)

Supplementary file 1: ICBPM4 Rules for missing, incomplete, multiple response and out of range data

1. Oversampling/Participation in local screening trials
   a) To handle oversampling in Ontario, include only the first 360 consecutive CRC patients;
   b) In jurisdictions with no national screen program: exclude patients participated in local screen trials.

2. Language/Participation in study/Presence of cancer
   Exclude patients who checked "No, I don't understand the language" or "I don't want to participate in this study" or "I don't have cancer".

3. Survey responders
   a) Exclude Patient/PCP/Specialist survey from the analysis, if it was not written by Patient/PCP/Specialist (example: a medical oncologist completed a PCP survey);
   b) In the case of duplicates, include only the first survey (example: 2 specialists completed surveys for the same patient).

4. Gender
   Exclude patients with unknown Gender.

5. Age
   a) Exclude patients with unknown age;
   b) Exclude patients younger 40 years;
   c) Use registry data, if Age is reported by both patient and registry.

6. No cancer or Previous cancer in the same organ
   a) Exclude patients with no cancer based on registry data;
   b) Exclude patients with previous cancer in the same organ based on data from registry or free-text for Presentation in the patient survey.

7. Date of consent
   Exclude patients with date of consent which is unknown, before 01.01.2013 or in the future.

8. Multiple responses to Dates
   If multiple responses were given to the dates (of first symptom; screening; first presentation to primary care; referral; diagnosis; treatment start), then use the earliest date.

9. Order of Dates
   The dates must be in the following order –
   a) First symptom; first presentation to Primary Care; referral; diagnosis; treatment start.
   b) Screening; diagnosis; treatment start.

   If not, check for mistakes.

10. Date of first symptom
    Date of first symptom is defined as date of first symptom from patient data.

11. Date of first presentation
    Date of first presentation to Primary Care is defined as (in the order of declining priority):
    a) date of first presentation to Primary Care from PCP data;
    b) date of first presentation to Primary Care and A&E from PCP data;
    c) date of first presentation to Primary Care from patient data.

For peer review only

12. Date of referral
Date of referral is defined as date of referral from PCP data.

13. Date of screening
Date of screening is defined as (in the order of declining priority):
a) date of screening from registry;
b) date of screening from patient data.

14. Date of diagnosis
Definition
a) If Registry reports both date of histological confirmation and date of confirming investigation, then use date of histological confirmation.
b) Date of diagnosis (based on patient data, PCP data, specialist data, registry data) is defined as (in the order of declining priority):
- date of diagnosis from registry;
- date of histological confirmation (from specialist data, PCP data);
- date of biopsy (from specialist data, PCP data);
- date of confirming investigation (from specialist data, PCP data);
- date of first hospital admission (from specialist data, PCP data);
- date of MDT confirmation (from specialist data, PCP data);
- date patient was told (from specialist data, PCP data);
- other date of diagnosis (from specialist data, PCP data, patient data);

Choose a Date from a lower level of hierarchy, if the Date from a higher level is after the Date of consent or more than 9 months (=271 days) before the Date of consent.

Exclusion criteria
a) Unknown date of diagnosis;
b) Date of diagnosis is after the date of consent;
c) Date of diagnosis is more than 9 months before the Date of consent.

15. Date of treatment start
a) Date of treatment start from patient data is defined as the earliest of the treatment dates for Surgery, Chemo, Radio and Other;
b) Date of treatment start (based on registry data, specialist data, patient data) is defined as (in the order of declining priority):
- date of treatment start from registry data,
- date of treatment start from specialist data,
- date of treatment start from patient data,
- anticipated date of treatment from patient data.

16. Imputation of missing day in the date
Imputation rules for missing day (given month and year are known):
a) Set missing day to '16';
b) Consider adjacent dates in a backwards order (from "Treatment" to "First symptom"). For each pair of such adjacent dates: If dates are not in a logical order (e.g. "Treatment" is before "Diagnosis"), but month and year are the same in both dates, and the day was imputed to '16' in one of the dates:
- Recode the day imputed earlier to '16' to the day from the adjacent date.

17. Considering time
If patient gave multiple answers to the "How long did you have symptoms before contacting a doctor?" question, then use the option with the shortest time interval.

18. Delay arranging appointment
If patient gave multiple answers to the "How long did it take to get an appointment with PCP?" question, then

For peer review only - http://bmjopen.bmj.com/site/about/guidelines.xhtml

use the option with the shortest time interval.

19. Duration of symptoms

If PCP gave multiple answers to the "Duration of symptoms" question, then use the option with the shortest time interval.

20. Definition of Presentation

A. *Define Presentation within a Data Source (Patient, PCP)*

1. Review the free-text for Presentation (Patient, PCP) and re-code, if possible.
2. If PCP reports 'VisitPCP and AE' or 'VisitPCP' as Presentation and no symptoms, then check Patient's records. If Patient reports 'Screening' and no symptoms, then re-code Presentation for this case as 'Screening'.
3. If PCP reports 'Screening' as Presentation and at least one symptom (or "Duration of Symptoms"), then re-code Presentation to 'Other non-screen-detected'-option.
4. If PCP reports 'Other' as Presentation and at least one symptom (or "Duration of Symptoms"), then re-code Presentation to 'Other non-screen-detected'-option.
5. If Patient reports 'Screening' as Presentation and at least one symptom (or date of first symptom), then re-code Presentation to 'Other non-screen-detected'- option.
6. If Patient reports 'Other' as Presentation and at least one symptom (or date of first symptom or "Considering time" or "Delay arranging appointment", then re-code Presentation to 'Other non-screen-detected'-option.
7. In the case of multiple Presentation responses (Patient, PCP sources) - use a single option (in the order of declining priority):

   a) 'VisitPCP and AE',
   b) 'VisitPCP´, 'AE´ (if both 'VisitPCP´ and 'AE´ are given, then re-code as 'VisitPCP and AE'),
   c) 'Other non-screen-detected',
   d) 'Screening',
   e) 'Investigation for another problem' ,
   f) 'Other"

For peer review only

B. *Define Presentation from Alternative Data*

If Presentation hasn't been reported in either of data sources, then define it as (in the order of declining priority):

1. 'Other non-screen-detected ', if PCP reports at least one symptom (or "Duration of symptoms");
2. 'Other non-screen-detected ', if Patient reports at least one symptom (or date of first symptom);
3. 'Other non-screen-detected ', if Patient reports "Considering time" or "Delay arranging appointment" and no screening date;
4. 'Screening', if Patient reports screening date and no symptoms and no date of first symptom;
5. 'Other non-screen-detected ', if jurisdiction=England, Age <58 or >76 years.

C. *Define Presentation from Data Source Hierarchy*

1. In Wales, England, Scotland, N Ireland and Manitoba: if Registry reports 'Screening' – use Presentation data from Registry data.

2. In Wales, England, Scotland, N Ireland and Manitoba: if Registry reports 'No Screening' – use Presentation data from (in the order of declining priority):
   a) PCP data;
   b) Patient data;

   If PCP (or Patient, in the case of PCP data is not available) reports 'Screening', then code Presentation as 'Other non-screen-detected'. If information from PCP and Patient datasets is missing, then code Presentation as 'Other non-screen-detected'.

3. In Wales, England, Scotland, N Ireland and Manitoba: if screening status from Registry is missing – use Presentation data from (in the order of declining priority):

a) PCP data;
b) Patient data;

4. For Denmark, Norway, Ontario and Victoria – use Presentation data from (in the order of declining priority):
   a) PCP data;
   b) Patient data.

5. In Sweden – use Presentation data from Patient data.

21. Patient interval

The Patient interval for non-screen-detected patients is defined as (in the order of declining priority):

a) "Date of first presentation to Primary Care" minus "Date of first symptom";
b) If the interval in (a) is unknown or negative: Calculate the interval as the low boundary of "Considering time" plus the low boundary of "Delay arranging appointment";
c) If the interval in (a) is unknown or negative and the interval in (b) is unknown: Calculate the interval as the low boundary of "Duration of symptoms interval".

22. Primary Care interval

The Primary Care interval for non-screen-detected is defined as "Date of referral" minus "Date of first presentation to Primary Care".

23. Diagnostic interval

a) The Diagnostic interval for non-screen-detected is defined as "Date of diagnosis" minus "Date of first presentation to Primary Care";
b) The Diagnostic interval for screen-detected patients is defined as "Date of diagnosis" minus "Date of screening".

24. Treatment interval

The Treatment interval is defined as "Date of treatment start" minus "Date of diagnosis".

25. Total interval

a) The Total interval for non-screen-detected patients is defined as "Date of treatment start" minus "Date of first symptom";
b) The Total interval for screen-detected patients is defined as "Date of treatment start" minus "Date of screening".

26. Range of Time intervals

The time intervals (Patient, Primary Care, Diagnosis, Treatment, Total) must be in range 0-1 year.

If > 1 year: set the interval to 365 days
If negative: set the interval to 0.

For each jurisdiction calculate the number of imputations due to:

a) unknown day in a date (given known month and year);
b) very large(>1 year) interval;
c) negative interval.

27. Type of treatment

If patient ticked both "Yes" and "No" as answers to the "Type of treatment (Surgery, Chemotherapy, Radiotherapy)" questions, then choose "Yes" answer.

28. Health state

If patient gave multiple answers to the "Health state" question, then use the option with a better health condition.

For peer review only - http://bmjopen.bmj.com/site/about/guidelines.xhtml

29. Comorbidity
 a) If patient ticked both "Yes" and "No" as answers to the "Comorbidity (Heart disease, Stroke, Lung disease, Diabetes)" questions, then choose "Yes" answer;
 b) If both patient and PCP report "Comorbidity", then use the PCP Data.

30. Ethnicity
 a) If patient didn't report "Ethnicity", then use the information from (in the order of declining priority):
   - "Ethnicity_Other_Details";
   - "Other main language spoken at home";
   - "The main language spoken at home" (only for Victoria);
   - "The main language spoken at home is the chief one for this jurisdiction"="Yes" given "Main language spoken at home is other than the main one for this jurisdiction"="No";
 b) Consider Ethnicity as unknown, if answers to the "Ethnicity" question are multiple and belong to different categories ('white', 'Asian', 'black', 'other').

31. Education
If patient gave multiple answers to the "Education" question, then use the option with a higher level of education.

32. Smoking Current
 a) If patient ticked both "Yes" and "No" as answers to the "Smoking Current" question, then use "Yes" answer;
 b) If patient hasn't ticked neither "Yes" nor "No, then consider this case as Unknown.

33. Smoking Number
If patient reports "SmokingNumber" as text, then re-code using following rules:

a) Where there is a number smoked /day – accept number;
b) Where a range has been given – take the upper value;
c) Where patient has put 10+ or 20+ - capture this as 11 or 21;
d) Where number of cigarettes smoked in the past and currently being smoked are provided - average the numbers;
e) Non entries code as "." ;
f) Non-smokers (eg, "nil", "N/A") are coded as "0".

34. Smoked ever
 a) If patient ticked both "Yes" and "No" as answers to the "Smoking ever" question, then use "Yes" answer;
 b) If patient hasn't ticked neither "Yes" nor "No": consider it as "Yes", if patient is a current smoker ("Smoking_Current="Yes"") or has specified a number of cigarettes ("SmokingNumber">0). Otherwise consider this case as Unknown.
 c) If patient has ticked "No": recode it to "Yes", if patient is a current smoker ("Smoking_Current="Yes").

35. Nature of referral
 a) Review free-text for " Nature of referral" (PCP Data) and re-code, if possible;
 b) In the case of multiple responses, use a single option as (in the order of declining priority):

   - "Referral for immediate admission";
   - "Urgent referral";
   - "Less urgent referral";
   - "General referral" ;
   - "No referral";
   - "Other".

For peer review only

36. Stage-TNM

a) If specialist gave multiple responses to the “Stage_TNM” question, then use the highest category;
b) If registry gave multiple responses to the “Stage_TNM”, then use a single option (in the order of declining priority):
   - stage at time of diagnosis
   - stage at surgery
   - stage at oncology
c) If “Stage_TNM” is reported by both the specialist and registry, then use the registry data;
d) If “Stage_TNM” is unknown or “not able to stage”, then use “Stage_Duke”.

37. Stage Dukes

a) If specialist gave multiple responses to the “Stage_Dukes’” question, then use the highest category;
b) If “Stage_Dukes” is reported by both the specialist and registry, then use the registry data.

For peer review only

ICBP
International Cancer
Benchmarking Partnership

International Cancer Benchmarking Partnership Module 4

Patient questionnaire

Colorectal Cancer

Thank you very much for taking the time to fill in this questionnaire – it should take about 20 minutes to complete. We are sending the questionnaire to a large sample of people who we understand have had a diagnosis of colorectal cancer. If this has been sent to you in error and you do not have cancer, please do not continue and return the documents in the prepaid envelope.

Sample

Our aim is to gain a better understanding of the process by which people have their cancer diagnosed. We would also like to find out more about the symptoms they experience (if any), and the pathway they follow from start of symptoms to treatment of their cancer. This will help in identifying ways in which cancers can be diagnosed quickly and effectively. Thank you once again for your time.

This information is confidential and will not be passed to anyone involved in your treatment.

Name:

Date of Birth:

Address:

Copyright property of the International Benchmarking Partnership (ICBP) and Module 4 collaborators. Usage must be authorised

ICBP
International Cancer
Benchmarking Partnership

Consent form

Please read the consent form and sign your name and date **BELOW**.

If you require any clarification, please do not hesitate to ring the study team members. Their contact details are found on the information sheet.

Please be reassured that your responses are completely confidential and will not be passed to anyone involved in your treatment. For the purposes of the study it is important that you agree to consent to all the statements listed below.

- I confirm that I have read the attached information sheet and I understand why the research is being done.
- I am willing for the team to request information from my GP and hospital doctors which is relevant to the audit as described in the information sheet.
- I give permission for my details (name, address) to be given to the cancer registry (NHS Information Centre for Health and Social Care) for follow up.
- I agree for the information I have provided and any other relevant information from my medical records to be stored as described in the information sheet under the custodianship of University College London.
- I consent to sharing of coded data which contains no personal identifiers between researchers, some of whom are located outside the European Union.
- I consent for use of my data if I become mentally incapacitated during the course of the project.

I agree to all the statements listed and consent to participate in the study.

Sample

Name (Please print)

Signature: Date:

If we have any questions, may we phone you for clarification?
(Please tick) ☐ **Yes** ☐ **No**

If **Yes**, please provide your telephone number:

Copyright property of the International Benchmarking Partnership (ICBP)
and Module 4 collaborators. Usage must be authorised

ICBP
International Cancer
Benchmarking Partnership

1. Please can you confirm the details of your GP/GP practice (name, practice address – as best as you can remember): We appreciate that you may have more than one GP involved in your care – in which case, we are interested in the GP you would say provides the majority of your care, particularly relating to the cancer you've had diagnosed.

Name of doctor

Name of practice

Address

Postcode

Town

Sample

Copyright property of the International Benchmarking Partnership (ICBP)
and Module 4 collaborators. Usage must be authorised

International Cancer Benchmarking Partnership

2. Which of the following **best describes** the events which led to your diagnosis of cancer? (please tick only **ONE** answer)

	✓
I had symptoms/I noticed a bodily change and went to see a doctor (e.g. GP)	
I had symptoms/I noticed a bodily change and went/was taken to Accident and Emergency (A&E)	
I had seen a doctor/GP with symptoms, but went/was taken to Accident and Emergency (A&E) when things worsened	
I was being investigated by my doctor(s) for another problem during which time the cancer was discovered	
I had a cancer screening test as part of a colorectal screening programme (e.g. the NHS Bowel Cancer Screening Programme in England – NHS BCSP)	
Other (please describe):	

Sample

What date did you have this screening test? If you cannot remember the exact date, you can fill in the month and the year.

Day (optional), month, year

D	D	M	M	Y	Y	Y	Y

Other (please describe):

Copyright property of the International Benchmarking Partnership (ICBP) and Module 4 collaborators. Usage must be authorised

ICBP
International Cancer
Benchmarking Partnership

3. The following health concerns or symptoms are commonly experienced with colorectal cancer.

Rectal bleeding or blood in faeces (poo)
Change in bowel habit
Unexplained tiredness/fatigue
Unexplained weight loss
Pelvic or abdominal pain
Skin went yellow
Abdominal distension/increased abdominal size/persistent bloating

Sample

Please write down ALL health concern(s) or symptom(s) you may have had before contacting a doctor or taking part in screening. It does not matter if they are not included in the list above:

Please write your health concern(s) or symptom(s) in the boxes below:
1)
2)
3)
4)
5)
6)

	✓
This is not applicable to me (e.g. I did not have any symptoms), please tick	

Copyright property of the International Benchmarking Partnership (ICBP)
and Module 4 collaborators. Usage must be authorised

ICBP
International Cancer
Benchmarking Partnership

4. Please write down your best estimate of the date you noticed the first of these health concern(s) or symptom(s). If you cannot remember the exact date, you can fill in the month and the year.

Day (optional), month, year

D	D	M	M	Y	Y	Y	Y

	✓
This is not applicable to me (e.g. I had no symptoms), please tick	

5. Approximately how long did you have health concern(s) or symptom(s) before contacting a doctor? (Please think of the first visit to the doctor, not re-visits after that). Please tick only **ONE** answer.

	✓
Less than 1 week	
1-2 weeks	
3-4 weeks	
5-7 weeks	
2-5 months	
6-12 months	
More than 12 months	

Sample

	✓
This is not applicable to me (e.g. I had no symptoms), please tick	

Copyright property of the International Benchmarking Partnership (ICBP) and Module 4 collaborators. Usage must be authorised

ICBP
International Cancer
Benchmarking Partnership

6a. Once you contacted a practice about your health concern(s) or symptom(s), how long did it take to get an appointment with a doctor? (Please think of the first visit to the doctor, to discuss your health concern(s) or symptom(s)).
Please tick only **ONE** answer.

	✓
Same day/next day	
Within 1 week	
1-2 weeks	
3-4 weeks	
Longer	

If there was no waiting time (e.g. you went/were taken to A&E), please tick this box	

This is not applicable to me (e.g. I had no symptoms), please tick	

Sample

6b. What was the date you first saw your doctor about your health concern(s) or symptom(s)? If you cannot remember the exact date, you can fill in the month and the year.

Day (optional), month, year

D	D	M	M	Y	Y	Y	Y

	✓
This is not applicable to me (e.g. I had no symptoms), please tick	

Copyright property of the International Benchmarking Partnership (ICBP)
and Module 4 collaborators. Usage must be authorised

ICBP
International Cancer
Benchmarking Partnership

7. How many times did you visit the following for the investigation of your symptoms before your cancer was diagnosed?

	Please write down the number of visits
GP	
Hospital	
Consultant/specialist outside of a hospital	

	✓
This is not applicable to me (e.g. I had no symptoms)	

8a. After your doctor referred you to a specialist, how long did it take you to get an appointment? Please tick only **ONE** answer.

Sample

	✓
Less than 1 week	
1-2 weeks	
3-4 weeks	
5-7 weeks	
2-5 months	
6-12 months	
More than 12 months	

	✓
This is not applicable to me (eg my doctor did not refer me), please tick	

Copyright property of the International Benchmarking Partnership (ICBP)
and Module 4 collaborators. Usage must be authorised

ICBP
International Cancer Benchmarking Partnership

8b. What was the date of your first appointment with a doctor, involved in investigating and/or treating your cancer, to whom you were referred?
If you cannot remember the exact date, you can fill in the month and the year.

Day (optional), month, year

D	D	M	M	Y	Y	Y	Y

This is not applicable to me (e.g. my doctor did not refer me), please tick	

9. What was the date you were told you had cancer? If you cannot remember the exact date, you can fill in the month and the year.

Day (optional), month, year

D	D	M	M	Y	Y	Y	Y

Sample

Copyright property of the International Benchmarking Partnership (ICBP) and Module 4 collaborators. Usage must be authorised

ICBP
International Cancer
Benchmarking Partnership

10. Have you had any of the following treatments for your cancer yet? If so, please can you estimate the date this treatment started? Please tick **ALL** that apply. If you cannot remember the exact date, you can fill in the month and the year.

	Type of treatment		Date of treatment (give first date if you had more than one)
a.	Surgery	☐ Yes ☐ No	Day (optional), month, year D D M M Y Y Y Y
b.	Chemotherapy	☐ Yes ☐ No	Day (optional), month, year D D M M Y Y Y Y
c.	Radiotherapy	☐ Yes ☐ No	Day (optional), month, year D D M M Y Y Y Y
d.	Other Please specify:	☐ Yes ☐ No	Day (optional), month, year D D M M Y Y Y Y
e.	Treatment not started yet	☐ Yes	

Sample

Copyright property of the International Benchmarking Partnership (ICBP) and Module 4 collaborators. Usage must be authorised

ICBP
International Cancer Benchmarking Partnership

11. Who is the consultant doctor who has taken responsibility for diagnosing and or/treating your cancer?

Name of consultant:

Hospital name:

Hospital department:

Please can you answer some more general questions about your health?
It will help us in interpreting your responses to this questionnaire to know about your general health and other health problems you may have had in the past.

12. Looking back to the 2 years before you were diagnosed with cancer, would you say your general health was (Please tick only **ONE** answer.)**:**

Sample

Very good	
Good	
Fair	
Poor	
Very poor	

13. Have you been treated before for any of the conditions below?
Please tick 'yes' or 'no' for each condition:

Heart disease	**Yes**	**No**
Stroke	**Yes**	**No**
Lung disease	**Yes**	**No**
Diabetes	**Yes**	**No**

Copyright property of the International Benchmarking Partnership (ICBP) and Module 4 collaborators. Usage must be authorised

ICBP
International Cancer
Benchmarking Partnership

Finally, a little more information about you. The information you provide below will help us to analyse the results of the survey in more detail.

14. Which of these best describes your ethnic group? (please tick one box, as appropriate). If you are descended from more than one ethnic or racial group, please tick the group you consider you belong to, or tick 'any other ethnic group'.

	✓		✓		✓		✓
White		Chinese		Black - Caribbean		Black - African	
Black - other		Indian		Pakistani		Bangladeshi	
Any other ethnic group, please specify							

Sample

15. What is the main language spoken in your home? Please tick

	✓
English	
Other, please specify:	

16. What is the highest level of education you have achieved?
Please tick only **ONE** answer.

	✓
Finished school at or before the age of fifteen	
Completed GCSEs, O-levels or equivalent	
Completed A Levels or equivalent	
Completed further education but not a degree	
Completed a Bachelor's degree / Masters degree / PhD	
Other, please specify:	

Copyright property of the International Benchmarking Partnership (ICBP) and Module 4 collaborators. Usage must be authorised

ICBP
International Cancer Benchmarking Partnership

17. **Have you ever smoked cigarettes, including hand-rolled ones, pipes or cigars?**

☐ Yes ☐ No

18. **Are you a current smoker, smoking either cigarettes, including hand-rolled ones, pipes or cigars?**

☐ Yes ☐ No

19. **If you are a current smoker or have smoked in the past, how many cigarettes, including hand-rolled ones, pipes or cigars on average do you smoke/have you smoked per day?**

Number per day:	

Sample

Copyright property of the International Benchmarking Partnership (ICBP)
and Module 4 collaborators. Usage must be authorised

ICBP International Cancer Benchmarking Partnership

20. Further comments

Please add anything else that you would like to tell us about your cancer diagnosis or treatment.

Sample

Thank you very much for taking the time to complete this questionnaire.

Copyright property of the International Benchmarking Partnership (ICBP) and Module 4 collaborators. Usage must be authorised

ICBP
International Cancer
Benchmarking Partnership

International Cancer Benchmarking Partnership Module 4

Primary Care Audit
Colorectal Cancer

Sample

For peer review only

Thank you very much for agreeing to fill in this questionnaire. As part of an international study examining differences in cancer survival, we are sending the questionnaire to health care providers of a sample of consented patients with cancer.

Our aim is to gain a better understanding of the process by which people have their cancer diagnosed – the symptoms they experience, and the pathway they follow from onset of symptoms to treatment of their cancer. This will help in identifying ways in which cancers can be diagnosed and treated quickly and effectively. Thank you once again for your time.

Please can you refer to your patient's notes in completing the questionnaire as this will help in obtaining accurate data on time points.

If you would prefer to return this questionnaire without the patient details, please tear off along the dotted line

ID-number: Jurisdiction-ID + Patient-ID:

Copyright property of the International Benchmarking Partnership (ICBP) and Module 4 collaborators. Usage must be authorised

ICBP
International Cancer
Benchmarking Partnership

Sample

For peer review only

Patient information

ID-number: Jurisdiction-ID + Patient-ID:

Full name:

Address:

Postcode:

Date of birth: | D | D | M | M | Y | Y | Y | Y |

Copyright property of the International Benchmarking Partnership (ICBP)
and Module 4 collaborators. Usage must be authorised

ICBP
International Cancer
Benchmarking Partnership

1. Duration of symptoms

Please estimate how long your patient had symptom(s), attributable to colorectal cancer, before attending your practice (or other health service).

We appreciate that identifying a 'date of first symptom' is not always straightforward – particularly when there are multiple and/or chronic symptoms. Nevertheless, we hope you can provide a 'best estimate'.

**Estimate of symptom duration** (please tick **one**):	✓	**What were the symptoms?** Please describe:
Less than 1 week		
1 to 4 weeks		
5 to 7 weeks		
2-5 months		
6-12 months		
More than 12 months		
Not possible to estimate		
No symptoms (e.g. screen detected cancers)		

Sample

For peer review only

Copyright property of the International Benchmarking Partnership (ICBP) and Module 4 collaborators. Usage must be authorised

ICBP
International Cancer
Benchmarking Partnership

2. Pathway of presentation

2.1 Through what route did the patient first present? Please tick **ONE**.

	✓	
Your patient first presented to primary care (either in-hours or out-of-hours)		Please can you provide your best approximation of the date of this primary care visit<br>D D M M Y Y Y Y
Your patient presented straight to A&E (with or without your involvement)		
Your patient first presented to primary care, but then at a later date presented to A&E as an emergency (with or without your involvement)		Please can you provide your best approximation of the date of this primary care visit<br>D D M M Y Y Y Y
Your patient's colorectal cancer was diagnosed through an organised screening programme (e.g. not as a result of investigation of symptoms)		
Other – please describe:		

Sample

For peer review only

Copyright property of the International Benchmarking Partnership (ICBP) and Module 4 collaborators. Usage must be authorised

ICBP
International Cancer
Benchmarking Partnership

3. Date you ordered any tests/investigations in response to symptom(s).

We are interested in any kind of tests/investigations (e.g. imaging etc) that you may have ordered. Please only consider the tests/investigations that you ordered yourself. Please tick **ALL** that apply and put in the date that the test/investigation was ordered:

	✓	
Blood test		D D M M Y Y Y Y
Faecal occult blood test (FOBT)		D D M M Y Y Y Y
Colonoscopy		D D M M Y Y Y Y
Sigmoidoscopy		D D M M Y Y Y Y
Double contrast barium enema (DCBE)		D D M M Y Y Y Y
Digital Rectal Exam (DRE)		D D M M Y Y Y Y
Virtual colonoscopy (computerised tomographic colonography)		D D M M Y Y Y Y
Other (please specify):		D D M M Y Y Y Y

Sample

4. Date of referral to specialist medical services

At what date did you **first** refer the patient to hospital or another specialist transferring the responsibility for on-going investigation/treatment to other medical services?

D D M M Y Y Y Y

Copyright property of the International Benchmarking Partnership (ICBP)
and Module 4 collaborators. Usage must be authorised

ICBP
International Cancer
Benchmarking Partnership

5. Nature of this referral

5.1 Do you know the date that the patient was seen for this referral?

☐ **Yes**, please provide the date: D D M M Y Y Y Y

☐ **No**

5.2 If you did make a referral to specialist services, which of the following best describes the nature/characteristics of this referral? Please tick **one**.

	✓
Emergency admission: a referral to A&E (or equivalent) for immediate admission	
An urgent referral for assessment of cancer symptoms/signs/test results (Note this will be within 2 weeks for England/Wales)	
A less urgent referral in which cancer is raised as a possibility (Note this will be greater than 2 weeks for England/Wales)	
A more general referral for investigation and assessment without cancer mentioned	
No referral was made	
Other – please describe	

5.3 Would you say this patient's diagnostic pathway was conducted predominantly in the public or private system? Please tick **one**.

	✓
Public healthcare system	
Private healthcare system	

Sample

For peer review only

Copyright property of the International Benchmarking Partnership (ICBP)
and Module 4 collaborators. Usage must be authorised

ICBP
International Cancer
Benchmarking Partnership

6. Date of colorectal cancer diagnosis

This can be decided in different ways. Please provide whichever of the following dates you have to hand. Please tick **all** that apply.

	✓	
Date of histological confirmation [ideal]		D D M M Y Y Y Y
Date results of investigation (histological or other) confirming cancer received		D D M M Y Y Y Y
Date patient was told		D D M M Y Y Y Y
Date biopsy undertaken		D D M M Y Y Y Y
Date patient was first admitted to hospital because of the malignancy		D D M M Y Y Y Y
Other (please specify)		D D M M Y Y Y Y

Sample

For peer review only

Copyright property of the International Benchmarking Partnership (ICBP) and Module 4 collaborators. Usage must be authorised

ICBP
International Cancer
Benchmarking Partnership

7. Additional information

Finally, we are interested to know what other conditions your patient has, and the severity/impact of these conditions

Have you and/or any of your partners treated this patient (or has the patient been to hospital) for any of the following conditions? Please tick **all** that apply:

Cardiovascular disease	☐ Yes	☐ No
Stroke	☐ Yes	☐ No
Lung disease	☐ Yes	☐ No
Diabetes	☐ Yes	☐ No

Sample

For peer review only

Are there any other comments you would like to make about this patient?

Name (and title):

Signature:

Date:

Thank you very much for taking the time to complete this questionnaire.

Copyright property of the International Benchmarking Partnership (ICBP)
and Module 4 collaborators. Usage must be authorised

ICBP
International Cancer Benchmarking Partnership

International Cancer Benchmarking Partnership Module 4

Specialist Care Audit

Colorectal Cancer

Thank you very much for agreeing to fill in this questionnaire – it should take about 10 minutes to complete. As part of an international study examining differences in cancer survival, we are sending the questionnaire to health care providers of a sample of patients with cancer.

Our aim is to gain a better understanding of the process by which people have their cancer diagnosed – the symptoms they experience, and the pathway they follow from onset of symptoms to treatment of their cancer. We hope you can help us with information on this patient's cancer journey once they were referred to specialist cancer services. This will help in identifying ways in which cancers can be diagnosed and treated quickly and effectively.

Thank you once again for your time

Please can you refer to your patient's notes in completing the questionnaire, as this will help in obtaining accurate data on time points.

Sample

For peer review only

If you would prefer to return this questionnaire without the patient details, please tear off along the dotted line.

Your patient

____

is participating in the study.

Copyright property of the International Benchmarking Partnership (ICBP) and Module 4 collaborators. Usage must be authorised

ICBP
International Cancer
Benchmarking Partnership

Sample

For peer review only

Patient information

ID-number: Jurisdiction-ID + Patient-ID:

Full name:

Address:

Postcode:

Date of birth: D D M M Y Y Y Y

Copyright property of the International Benchmarking Partnership (ICBP)
and Module 4 collaborators. Usage must be authorised

ICBP
International Cancer
Benchmarking Partnership

1. Date patient first attended hospital/specialist services related to their cancer diagnosis. We appreciate this date can at times be difficult to identify, particularly when there have been multiple visits in the lead up to a definitive diagnosis. Put another way, it's the date that the hospital/specialist service assumed responsibility for on-going investigation/treatment for your patient.

Day (optional), month, year

D	D	M	M	Y	Y	Y	Y

2. How was the patient referred to the hospital/specialist services related to their cancer diagnosis? Please tick.

Was it through a:

	✓		✓
GP referral		Screening	
Referral from general surgery clinic		Medical specialist/ Consultant referral	
Other referral – please specify:			

3. Where did this first contact/appointment happen? Please tick.
Which of the following best describes where this first contact/appointment took place?

	✓		✓
Emergency department ('A&E')		Medical outpatient department, please specify which department	
Oncology general outpatient department		Surgical outpatient department, please specify which department	
Other – please specify:			

Sample

For peer review only

Copyright property of the International Benchmarking Partnership (ICBP) and Module 4 collaborators. Usage must be authorised

ICBP
International Cancer
Benchmarking Partnership

4. Date of diagnosis

This can be decided in different ways.
Please tick and complete as many of the following dates as possible.

	✓	
Date of histological confirmation (ideal)		Day (optional), month, year D D M M Y Y Y Y
Date results of investigation confirming cancer received		Day (optional), month, year D D M M Y Y Y Y
Date patient was told		Day (optional), month, year D D M M Y Y Y Y
Date of biopsy		Day (optional), month, year D D M M Y Y Y Y
Date patient was first admitted to hospital because of the malignancy		Day (optional), month, year D D M M Y Y Y Y
Date of MDT confirmation of diagnosis		Day (optional), month, year D D M M Y Y Y Y
Other (please specify):		Day (optional), month, year D D M M Y Y Y Y

Sample

Copyright property of the International Benchmarking Partnership (ICBP)
and Module 4 collaborators. Usage must be authorised

ICBP
International Cancer
Benchmarking Partnership

5. Date treatment for the cancer commenced

Based on your records, when would you say that any treatment specifically targeting the patient's cancer started?

Day (optional), month, year

D	D	M	M	Y	Y	Y	Y

6. Additional information

Please can you provide any further information on the patient's cancer:

**TNM**, please tick as appropriate:		**Duke's**, please tick as appropriate:	
0		A	
I		B	
IIA		C	
IIB		D	
IIC			
IIIA			
IIIB			
IIIC			
IV			
Not able to stage			

Sample

For peer review only

Copyright property of the International Benchmarking Partnership (ICBP)
and Module 4 collaborators. Usage must be authorised

ICBP
International Cancer
Benchmarking Partnership

6.1 Histological subtype:

	✓
Adenocarcinoma	
Mucinous (colloid) adenocarcinoma	
Signet-ring cell carcinoma	
Other (please specify):	

Sample
For peer review only

Copyright property of the International Benchmarking Partnership (ICBP)
and Module 4 collaborators. Usage must be authorised

ICBP
International Cancer
Benchmarking Partnership

Further comments

Sample
For peer review only

Copyright property of the International Benchmarking Partnership (ICBP)
and Module 4 collaborators. Usage must be authorised

ICBP
International Cancer
Benchmarking Partnership

Name (and title): ______________________

Signature: ______________________

Date: ______________________

Sample

For peer review only

Are you a ... (please tick below): ✓

Surgeon	
Medical Oncologist	
Clinical Oncologist	
Clinical Nurse Specialist	
Other (please specify):	

Thank you very much for taking the time to complete this questionnaire.

Copyright property of the International Benchmarking Partnership (ICBP)
and Module 4 collaborators. Usage must be authorised

Supplementary File 5 – Ethical approvals, recruitment practices, ICBP M4 working group and ARG

Section 1 – Ethical and other approvals obtained in each Module 4 participating jurisdiction

	Date of Ethics Approval	Approvals obtained	Reference
**Victoria**	4 September 2012	Cancer Council Victoria Human Research Ethics Committee	HREC 1125
**Manitoba**	7 March 2013<br><br>15 April 2013	Health Research Ethics Board, University of Manitoba<br><br>Research Resource Ethics Committee, CancerCare Manitoba	HS15227 (H2012:105)<br><br>RRIC#28-2012
**Ontario**	7 November 2013<br>28 January 2014	University of Toronto Research Ethics Board	27881
**Denmark**	6 August 2013<br><br>19 June 2013	The Danish Data Protection Agency<br><br>According to Danish law and the Central Denmark Region Committees on Health Research Ethics, approval by the National Committee on Health Research Ethics was not required as no biomedical intervention was performed.	2013-41-2030<br><br>1-10-72-20-13
**Sweden**	23 October 2013	Ethics Review Board, Uppsala	2013/306
**Norway**	04 April 2013	Regional committees for medical and health research ethics	2013/136/REK nord
**Wales**	16 November 2012	NRES Committee East Midlands – Derby 2, local R&D for each health board	11/EM/0420
**Scotland**	16 November 2012	NRES Committee East Midlands – Derby 2,<br>R&D for each health board,<br>Privacy Advisory Committee,<br>CHI Advisory Group	11/EM/0420
**N Ireland**	1 June 2012	ORECNI Ethical approval, local governance for each health Trust	12/NI/0053
**England**	16 November 2012	NRES Committee East Midlands – Derby 2<br>R&D for each Clinical Research Network	11/EM/0420

For peer review only - http://bmjopen.bmj.com/site/about/guidelines.xhtml

Section 2 – Local recruitment practice in each Module 4 participating jurisdiction

	Recruitment practice variation
**Victoria**	The relevant healthcare professional confirmed eligibility prior to questionnaire mail-out to patients. Additional patients were recruited (above the required 200 symptomatic CRC patients) to meet the needs of a local study.
**Manitoba**	The data from cancer treatment specialists was not available.
**Ontario**	Additional patients were recruited (above the required 200 symptomatic CRC patients) to meet the needs of a local study.
**Denmark**	The cancer treatment specialist data were completed using clinical databases instead of through a survey.
**Sweden**	Only patients answered the survey – no primary care or cancer treatment specialist data available.
**Norway**	Some patients received and completed their surveys up to 9 months post diagnosis; their data were included (although flagged for subsequent analysis of any resulting sampling bias).
**Wales**	No variation.
**Scotland**	No variation.
**N Ireland**	The cancer treatment specialist data were collected directly from registries instead of through a survey. Some screen-detected cancer patients were excluded in the identification process.
**England**	No variation.

For peer review only

Section 3 – ICBP Module 4 Working Group

Alina Zalounina Falborg, Research Unit for General Practice, Department of Public Health, Aarhus University, Bartholins Allé 2, 8000 Aarhus C, Denmark

Andriana Barisic, Department of Prevention and Cancer Control, Cancer Care Ontario, 620 University Avenue, Toronto, Ontario, M5G 2L7, Canada

Anna Gavin, Northern Ireland Cancer Registry, Centre for Public Health, Queen's University Belfast, Mulhouse Building, Mulhouse Road, Belfast, BT12 6DP, United Kingdom

Anne Kari Knudsen, European Palliative Care Research Centre (PRC), Department of Oncology, Oslo University Hospital and Institute of Clinical Medicine, University of Oslo, Oslo, N-0424 Oslo, Norway

Breann Hawryluk, Department of Patient Navigation, Cancer Care Manitoba, 675 McDermot Street, Winnipeg, Manitoba, MB R3E 0V9, Canada

Chantelle Anandan, Centre for Population Health Sciences, University of Edinburgh, Doorway 1, Medical Quad Teviot Place, Edinburgh, EH8 9DX, United Kingdom

Conan Donnelly, Centre for Public Health, Queen's University Belfast, Mulhouse Building, Mulhouse Road, Belfast, BT12 6DP, United Kingdom

David H Brewster, Scottish Cancer Registry, Information Services Division, NHS National Services Scotland, Gyle Square, 1 South Gyle Crescent, Edinburgh, EH12 9EB, United Kingdom; Centre for Population Health Sciences, University of Edinburgh, Teviot Place, Edinburgh, EH8 9DX, United Kingdom

David Weller, Centre for Population Health Sciences, University of Edinburgh, Doorway 1, Medical Quad Teviot Place, Edinburgh, EH8 9DX, United Kingdom

Donna Turner, Population Oncology, Cancer Care Manitoba, 675 McDermot Street, Winnipeg, Manitoba, MB R3E 0V9, Canada

Elizabeth Harland, Department of Epidemiology and Cancer Registry, CancerCare Manitoba, 675 McDermot Street, Winnipeg, MB R3E 0V9, Manitoba

Eva Grunfeld, Knowledge Translation Research Network Health Services Research Program, Ontario Institute for Cancer Research; Professor and Vice Chair Research Department of Family and Community Medicine, University of Toronto, 500 University Avenue, Toronto, Ontario, M5G 1V7, Canada

Evangelia Ourania Fourkala, Gynaecological Cancer Research Centre, Women's Cancer, Institute for Women's Health, University College London, London, WC1E 6BT, United Kingdom

Henry Jensen, Research Unit for General Practice, Department of Public Health, Aarhus University, Bartholins Allé 2, 8000 Aarhus C, Denmark

For peer review only

Irene Reguilon, International Cancer Benchmarking Partnership, Cancer Research UK, London, EC1V 4AD, United Kingdom

Jackie Boylan, Centre for Public Health, Queen's University Belfast, Mulhouse Building, Mulhouse Road, Belfast, BT12 6DP, United Kingdom

Jacqueline Kelly, Northern Ireland Cancer Registry, Centre for Public Health, Queen's University Belfast, Mulhouse Building, Mulhouse Road, Belfast, BT12 6DP, United Kingdom

Jatinderpal Kalsi, Gynaecological Cancer Research Centre, Women's Cancer, Institute for Women's Health, University College London, London, WC1E 6BT, United Kingdom

John Butler, The Royal Marsden, Fulham Road, London, SW3 6JJ, United Kingdom

Kerry Moore, Centre for Public Health, Queen's University Belfast, Mulhouse Building, Mulhouse Road, Belfast, BT12 6DP, United Kingdom

Maria Rejmyr Davis, Southern Sweden Regional Cancer Center, Medicon Village, Scheelevägen 8, building 404, 223 81 Lund, Sweden

Martin Malmberg, Department of Oncology, Lund University Hospital, SE-221 85 Lund, Sweden

Mats Lambe, Regional Cancer Center Uppsala and Department of Medical Epidemiology and Biostatics, Karolinska Institutet, SE-171 77 Stockholm, Sweden

Oliver Bucher, Department of Epidemiology and Cancer Registry, CancerCare Manitoba, 675 McDermot Street, Winnipeg, MB R3E 0V9, Manitoba

Peter Vedsted, Research Unit for General Practice, Department of Public Health, Aarhus University, Bartholins Allé 2, 8000 Aarhus C, Denmark

Rebecca-Jane Law, North Wales Centre for Primary Care Research, Bangor University, Cambrian House 2, Wrexham Technology Park, Wrexham, LL13 7YP, United Kingdom

Rebecca Bergin, Centre for Behavioural Research in Cancer, 615 St Kilda Rd, Melbourne, Victoria, 3004, Australia; Department of General Practice, University of Melbourne, 200 Berkeley St, Victoria, 3053, Australia

Richard D Neal, North Wales Centre for Primary Care Research, Bangor University, Cambrian House 2, Wrexham Technology Park, Wrexham, LL13 7YP, United Kingdom; Academic Unit of Primary Care, Leeds Institute of Health Sciences, University of Leeds, Leeds, LS2 9NL, United Kingdom

Samantha Harrison, Early Diagnosis and International Cancer Benchmarking Partnership, Policy and Information, Cancer Research UK, London, EC1V 4AD, United Kingdom

For peer review only - http://bmjopen.bmj.com/site/about/guidelines.xhtml

Sigrun Saur Almberg, Department of Cancer Research and Molecular Medicine, Faculty of Medicine, Norwegian University of Science and Technology (NTNU), N-7491 Trondheim, Norway

Therese Kearney, Northern Ireland Cancer Registry, Centre for Public Health, Queen's University Belfast, Mulhouse Building, Mulhouse Road, Belfast, BT12 6DP, United Kingdom

Victoria Cairnduff, Northern Ireland Cancer Registry, Centre for Public Health, Queen's University Belfast, Mulhouse Building, Mulhouse Road, Belfast, BT12 6DP, United Kingdom

Victoria Hammersley, Centre for Population Health Sciences, University of Edinburgh, Doorway 1, Medical Quad Teviot Place, Edinburgh, EH8 9DX, United Kingdom

Victoria White, Centre for Behavioural Research in Cancer, Cancer Council Victoria, 615 St Kilda Road, Melbourne, Victoria, 3004, Australia; School of Psychology Deakin University, Geelong, Victoria, 3217, Australia

Usha Menon, Gynaecological Cancer Research Centre, Women's Cancer, Institute for Women's Health, University College London, London, WC1E 6BT, United Kingdom

Yulan Lin, European Palliative Care Research Centre (PRC), Department of Oncology, Oslo University Hospital and Institute of Clinical Medicine, University of Oslo, Oslo, N-0424 Oslo, Norway

For peer review only

Section 4 – ICBP Module 4 Academic Reference Group:

Prof Jan Willem Coebergh, Professor of Cancer Surveillance, Department of Public Health, Erasmus Universiteit Rotterdam, Rotterdam, the Netherlands

Jon Emery, Professor of Primary Care Cancer Research, University of Melbourne and Clinical Professor of General Practice, University of Western Australia, Australia

Dr Stefan Bergström, Senior consultant oncologist, Department of Oncology, Gävle, Sweden

Dr Monique E van Leerdam, Erasmus MC University Medical Centre, the Netherlands

Prof Marie-Louise Essink-Bot, Academic Medical Centre, Amsterdam University, the Netherlands

Prof Una MacLeod, Senior Lecturer in General Practice and Primary Care, Hull-York Medical School, United Kingdom

For peer review only

For peer review only - http://bmjopen.bmj.com/site/about/guidelines.xhtml

Supplementary File 6 – graphs of regression analysis for symptomatic patients (based on Table 6). The difference in the length of jurisdiction's intervals are shown compared to the reference Wales (days).

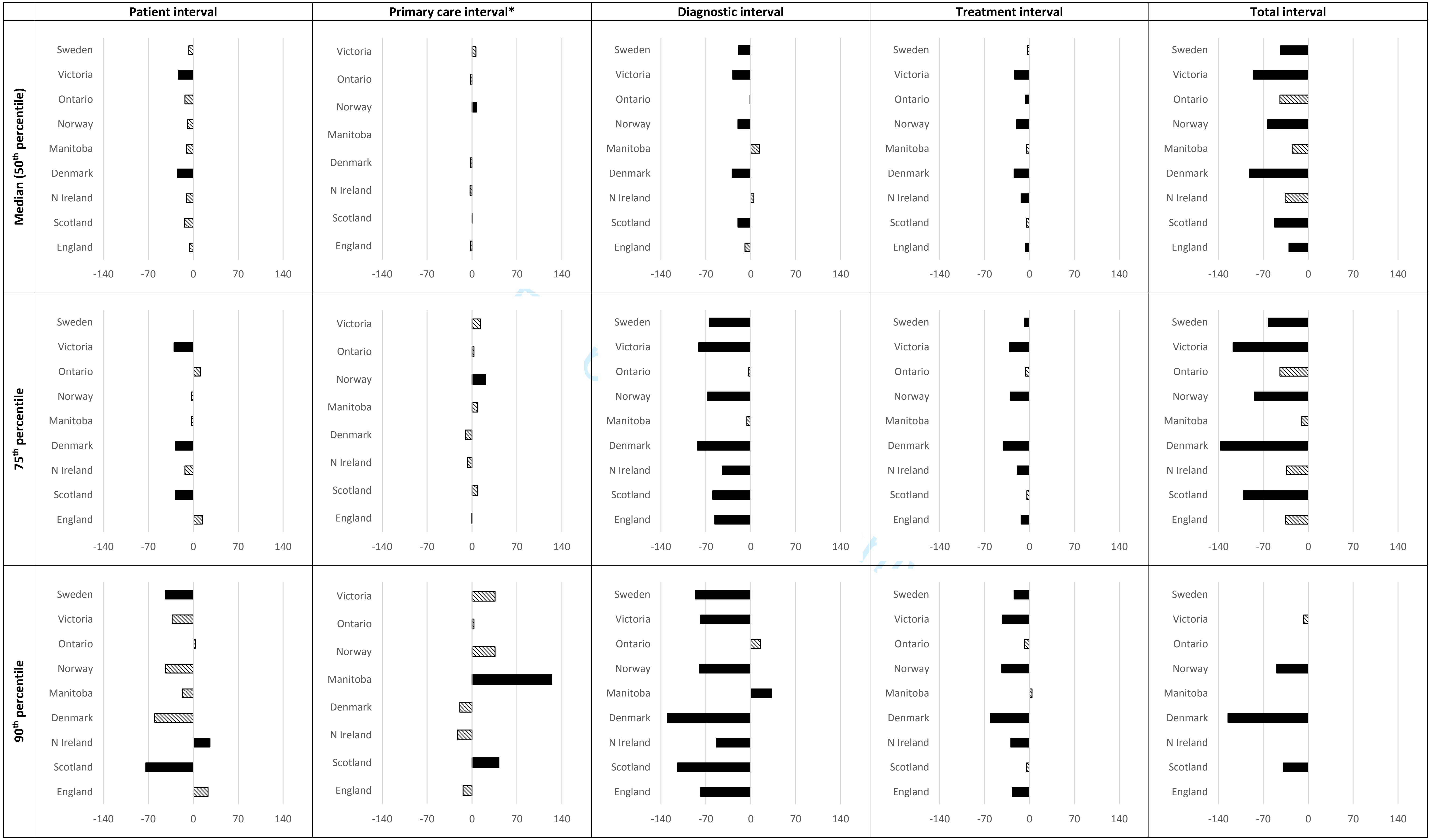

* Sweden did not provide any data for the primary care interval, and so has not been included in these graphs.

Differences in interval lengths (in days) are shown for the median, 75th and 90th percentiles compared to the reference used for the regression analyses, Wales. Wales is represented by the axis, with jurisdictions with shorter intervals shown to the left of the axis, and jurisdictions with longer intervals shown to the right of the axis for each graph. Statistically significant results are shown in solid bars, whilst non-significant results are shown with a pattern fill.

The RECORD statement – checklist of items, extended from the STROBE statement, that should be reported in observational studies using routinely collected health data.

	Item No.	STROBE items	Location in manuscript where items are reported	RECORD items	Location in manuscript where items are reported
**Title and abstract**					
	1	(a) Indicate the study's design with a commonly used term in the title or the abstract (b) Provide in the abstract an informative and balanced summary of what was done and what was found	Title and abstract – p 1 and 3	RECORD 1.1: The type of data used should be specified in the title or abstract. When possible, the name of the databases used should be included.<br><br>RECORD 1.2: If applicable, the geographic region and timeframe within which the study took place should be reported in the title or abstract.<br><br>RECORD 1.3: If linkage between databases was conducted for the study, this should be clearly stated in the title or abstract.	Abstract – p 3<br><br>Abstract – p 3<br><br>N/A
**Introduction**					
Background rationale	2	Explain the scientific background and rationale for the investigation being reported	Background – p 4		
Objectives	3	State specific objectives, including any prespecified hypotheses	Background – p 4		
**Methods**					
Study Design	4	Present key elements of study design early in the paper	Methods – p 5		
Setting	5	Describe the setting, locations, and relevant dates, including periods of recruitment, exposure, follow-up, and data collection	Methods – p 5-7		
Participants	6	*(a) Cohort study* - Give the eligibility criteria, and the	Methods – p 6 – and as reference to	RECORD 6.1: The methods of study population selection (such as codes or	Provided as reference to

For peer review only

		sources and methods of selection of participants. Describe methods of follow-up<br>*Case-control study* - Give the eligibility criteria, and the sources and methods of case ascertainment and control selection. Give the rationale for the choice of cases and controls<br>*Cross-sectional study* - Give the eligibility criteria, and the sources and methods of selection of participants<br><br>*(b) Cohort study* - For matched studies, give matching criteria and number of exposed and unexposed<br>*Case-control study* - For matched studies, give matching criteria and the number of controls per case	previous paper.<br><br>N/A	algorithms used to identify subjects) should be listed in detail. If this is not possible, an explanation should be provided.<br><br>RECORD 6.2: Any validation studies of the codes or algorithms used to select the population should be referenced. If validation was conducted for this study and not published elsewhere, detailed methods and results should be provided.<br><br>RECORD 6.3: If the study involved linkage of databases, consider use of a flow diagram or other graphical display to demonstrate the data linkage process, including the number of individuals with linked data at each stage.	previous paper.<br><br>Provided as appendix and in reference to previous paper.<br><br>N/A
Variables	7	Clearly define all outcomes, exposures, predictors, potential confounders, and effect modifiers. Give diagnostic criteria, if applicable.	Methods – p 7-8 – and as reference to previous paper.	RECORD 7.1: A complete list of codes and algorithms used to classify exposures, outcomes, confounders, and effect modifiers should be provided. If these cannot be reported, an explanation should be provided.	N/A
Data sources/ measurement	8	For each variable of interest, give sources of data and details of methods of assessment (measurement).<br>Describe comparability of assessment methods if there is more than one group	Methods – p 6-9 – and as reference to previous paper.		
Bias	9	Describe any efforts to address potential sources of bias	Methods – p 8-9, discussion – p 21-22 – and as reference to		

For peer review only - http://bmjopen.bmj.com/site/about/guidelines.xhtml

			previous paper.		
Study size	10	Explain how the study size was arrived at	Methods – p 5 – and as reference to previous paper.		
Quantitative variables	11	Explain how quantitative variables were handled in the analyses. If applicable, describe which groupings were chosen, and why	Methods – p 8-9 – and as reference to previous paper.		
Statistical methods	12	(a) Describe all statistical methods, including those used to control for confounding<br>(b) Describe any methods used to examine subgroups and interactions<br>(c) Explain how missing data were addressed<br>(d) *Cohort study* - If applicable, explain how loss to follow-up was addressed<br>*Case-control study* - If applicable, explain how matching of cases and controls was addressed<br>*Cross-sectional study* - If applicable, describe analytical methods taking account of sampling strategy<br>(e) Describe any sensitivity analyses	Methods – p 8-9 – and as reference to previous paper.		
Data access and cleaning methods		..		RECORD 12.1: Authors should describe the extent to which the investigators had access to the database population used to create the study population.	N/A

For peer review only

				RECORD 12.2: Authors should provide information on the data cleaning methods used in the study.	Methods – p 6
Linkage		..		RECORD 12.3: State whether the study included person-level, institutional-level, or other data linkage across two or more databases. The methods of linkage and methods of linkage quality evaluation should be provided.	N/A
**Results**					
Participants	13	(a) Report the numbers of individuals at each stage of the study (*e.g.*, numbers potentially eligible, examined for eligibility, confirmed eligible, included in the study, completing follow-up, and analysed)<br>(b) Give reasons for non-participation at each stage.<br>(c) Consider use of a flow diagram	Results and as table – p 9-12. Flow diagram in previous paper, referenced.	RECORD 13.1: Describe in detail the selection of the persons included in the study (*i.e.,* study population selection) including filtering based on data quality, data availability and linkage. The selection of included persons can be described in the text and/or by means of the study flow diagram.	Results and as table. Flow diagram in previous paper, referenced.
Descriptive data	14	(a) Give characteristics of study participants (*e.g.*, demographic, clinical, social) and information on exposures and potential confounders<br>(b) Indicate the number of participants with missing data for each variable of interest<br>(c) *Cohort study* - summarise follow-up time (*e.g.*, average and total amount)	Results and as table – p 9-12.		
Outcome data	15	*Cohort study* - Report numbers of outcome events or summary measures over time<br>*Case-control study* - Report numbers in each exposure category, or summary measures	Results and as table – p 12-19.		

For peer review only

		of exposure<br>*Cross-sectional study* - Report numbers of outcome events or summary measures			
Main results	16	(a) Give unadjusted estimates and, if applicable, confounder-adjusted estimates and their precision (e.g., 95% confidence interval). Make clear which confounders were adjusted for and why they were included<br>(b) Report category boundaries when continuous variables were categorized<br>(c) If relevant, consider translating estimates of relative risk into absolute risk for a meaningful time period	Results and as table – p 12-19.		
Other analyses	17	Report other analyses done—e.g., analyses of subgroups and interactions, and sensitivity analyses	Results – p 19.		
**Discussion**					
Key results	18	Summarise key results with reference to study objectives	Discussion – p 20		
Limitations	19	Discuss limitations of the study, taking into account sources of potential bias or imprecision. Discuss both direction and magnitude of any potential bias	Discussion – p21-22	RECORD 19.1: Discuss the implications of using data that were not created or collected to answer the specific research question(s). Include discussion of misclassification bias, unmeasured confounding, missing data, and changing eligibility over time, as they pertain to the study being reported.	Discussion – p21-22
Interpretation	20	Give a cautious overall interpretation of results considering objectives, limitations, multiplicity of analyses, results from similar	Discussion – p22-23		

For peer review only

		studies, and other relevant evidence			
Generalisability	21	Discuss the generalisability (external validity) of the study results	Discussion, conclusion – p22-23.		
**Other Information**					
Funding	22	Give the source of funding and the role of the funders for the present study and, if applicable, for the original study on which the present article is based	Funding statement – p 24		
Accessibility of protocol, raw data, and programming code		..		RECORD 22.1: Authors should provide information on how to access any supplemental information such as the study protocol, raw data, or programming code.	Availability of data and material statement – p 25

*Reference: Benchimol EI, Smeeth L, Guttmann A, Harron K, Moher D, Petersen I, Sørensen HT, von Elm E, Langan SM, the RECORD Working Committee. The REporting of studies Conducted using Observational Routinely-collected health Data (RECORD) Statement. *PLoS Medicine* 2015; in press.

*Checklist is protected under Creative Commons Attribution (CC BY) license.

For peer review only
